# Colonization of different biomes drove the diversification of the Neotropical *Eidmanacris* crickets (Insecta: Orthoptera: Grylloidea: Phalangopsidae)

**DOI:** 10.1371/journal.pone.0245325

**Published:** 2021-01-15

**Authors:** Lucas Denadai de Campos, Pedro Guilherme Barrios de Souza-Dias, Laure Desutter-Grandcolas, Silvio Shigueo Nihei

**Affiliations:** 1 Departamento de Zoologia, Instituto de Biociências, Universidade de São Paulo, São Paulo, Brazil; 2 Institut de Systématique, Évolution et Biodiversité, Muséum national d’Histoire naturelle, Sorbonne Université, CNRS, UPMC, EPHE, UA, Paris, France; 3 Departamento de Entomologia, Museu Nacional, Universidade Federal do Rio de Janeiro, Rio de Janeiro, Brazil; Nanjing Agricultural University, CHINA

## Abstract

The phylogeny of the cricket genus *Eidmanacris* is used to analyse its historical distribution and diversification in three South American biomes: Atlantic Forest, Cerrado, and Chiquitano Dry Forest. A morphological phylogeny with all the 29 species of *Eidmanacris* and the Geographically explicit Event Model (GEM) is used to explain their colonization and diversification through three different biomes and their ancestral habitats and distributional areas. We analysed ecologically-significant characters, such as body size and metanotal characters, to test whether if morphology, habitat, or behaviour are connected. The relations of these features with the colonisation of wetter or drier biomes based on the distributional area, phylogeny and diversity of the genus were also tested. The results show that the ancestral distribution of the genus was the Atlantic Forest, and that biome occupancy, habitat, size, and mating behaviour evolved congruently through the phylogeny, drawing a coherent pattern of changes through *Eidmanacris* evolution toward the colonisation of drier biomes. Our results indicate that gallery forests could play a key role in the distribution and diversification of *Eidmanacris* species.

## Introduction

The Neotropical Region is the most species-rich region of the World, and the origins, diversification, and evolution of Neotropical organisms have been main subjects of interest for the last 150 years [1]. Until recently, most studies have focused on the Amazon Rainforest [2, 3]. However, other biomes have played a central role in the diversification of Neotropical biotas, like the Atlantic Forest and the drier vegetations located south and west of the Amazon Rainforest, i.e. the dry forests, Cerrado, and Chaco [4].

With more 8000 endemic species, the Atlantic Forest is the second-largest tropical forest in South America after the Amazon and is considered the most threatened diversity hotspot in South America [5]. The Atlantic Forest covers a wide range of vegetation formations, from tropical to subtropical and with heterogeneous composition, and covers elevations from the sea level to 2900 m [6]. It is separated from the Amazon Rainforest by the diagonal open formations, composed by Caatinga, Chaco and Cerrado [7], all equally threatened. The deciduous and semideciduous Chiquitano Dry Forest is a transition zone between the moist Amazon Rainforest and the dry forest of the Chaco [8], located in the east of Santa Cruz, Bolivia, and extending into western of Mato Grosso, Brazil. That forest is characterized by a pronounced dry season lasting 5–6 months per year, with a sparse and lower vegetation than tropical rain forests [7, 9]. Furthermore, it is one of the most diverse dry forests in the world [10]. The Cerrado hotspot covers more than 2 million km^2^ of South America [5, 11], between the Amazon, the southern Atlantic Forest, Caatinga and Chaco [12–14]. It is a savannah vegetation with nutrient-poor, deep and well-drained soils [15], which physiognomies vary from an open field (“Campo limpo”) to a relatively tall and closed forest (“Cerradão”) [16]. It is crossed by gallery forests mainly formed by headwaters of rivers located in the plateaus of the Cerrado region [17].

The diversity of landscapes and environments of these biomes and their ecotone transitions may have represented evolutionary opportunities or an obstacle for the diversifications and/or dispersion of forest biotas. Several studies support the hypothesis that the Atlantic Forest was connected to the Amazon Rainforest in the past through gallery forests and/or by forests expansions [18–23], which would have allowed the colonisation of the Cerrado by forest-dependent organisms, e.g. [24–27], as well as faunal exchange between the two large South American forests through mesic corridors. Some groups of organisms are common to both forests, i.e. wood trees [7, 23], small mammals like rodents and marsupials [28, 29] and butterflies [21]. However, some vertebrate groups are exclusively present in the Amazon Rainforest, e.g. [30, 31], or in the Atlantic Forest, e.g. [32, 33], due to specific sensitivity to different climates, vicariance (geographical or ecological), specific niches and/or many other biotic or abiotic variables [1].

The colonisation of different biomes by a given evolutionary lineage, or its limitation to one biome, may be related to the biotic properties of the lineage, which must cope with environmental variables [34, 35]. This not only relates to climatic adaptation, but also resource use, and behavioural differences, in particular, reproduction.

Crickets are highly diversified in the Neotropical Region [36–40], but they have been little used to test distributional hypotheses, due to the limited taxonomic knowledge, with many taxa still awaiting description, and the lack of phylogenetic analyses of well-supported monophyletic taxa [41]. In other hotspot areas, crickets are important model groups for studying modifications of insect communities through forest succession or the influence of invasive species [42, 43].

Here we analyse the diversification of the cricket genus *Eidmanacris* Chopard (Insecta, Orthoptera, Grylloidea, Phalangopsidae), which is distributed in the Atlantic Forest, Cerrado, and Chiquitano Dry Forest, but is absent from the Amazon Rainforest. Being flightless, the species of *Eidmanacris*, like other micropterous or apterous orthopterans, are potential models for studying migration corridors [44, 45]. *Eidmanacris* was recently revised, based on a large sampling effort through forested and open biomes [46–48]. In the present paper, we perform an event-based biogeographical analysis (Geographically explicit Event Model–GEM) [49] using a morphology-based phylogenetic analysis including all the 29 species of *Eidmanacris*, to answer how did *Eidmanacris* colonise the three different biomes where it occurs today and what are the ancestral habitats and distributional areas. We also performed an ancestral state reconstruction analysis to examine ecologically-significant characters for the diversity of the genus, such as body size (resource use, climatic adaptation) and genital characters (mating behaviour), in order to verify if morphology, habitat, or behaviour are connected, and if these features are related to the colonisation of wetter or drier biomes. Furthermore, we discuss the conservation of the biomes in which *Eidmanacris* is present.

## Materials and methods

### Taxon sampling

The ingroup includes all the 29 species of *Eidmanacris* described to date (S1 Table) [48]. For *E*. *paramarmorata* Desutter-Grandcolas and *E*. *longa* Gorochov, we used data from the original descriptions and previous studies [50, 51]. High-resolution images of the holotypes of *E*. *marmorata*, E. *paramarmorata* and *E*. *speluncae* (Mello-Leitão) were accessed from the online catalog *Orthoptera Species File* [52].

The specimens examined belong to the following institutions: Laboratório de Insetos do Departamento de Zoologia da UNESP de Botucatu, São Paulo (UBTU); Laboratório de Orthopterologia da Universidade de Viçosa, Minas Gerais (UFV); Muséum national d’Histoire naturelle, Paris (MNHN); Museu de Zoologia da Universidade de São Paulo, São Paulo (MZSP); Museu de Zoologia da Universidade Estadual de Feira de Santana, Bahia (MZUEFS); The Academy of Natural Sciences of Drexel University, Philadelphia (ANSP). Additional material was collected in the field by LDC and PGBS and deposited in MZSP. Natural history observations were made during fieldworks. Outgroups were used to root the tree and polarize the characters [53]. Outgroups were chosen based on the phylogeny of Souza-Dias [54], which included four species of *Eidmanacris*. Nine outgroup taxa were selected (S2 Table): *Adenopygus heikoi* Bolfarini & de Mello; *Bambuina bambui* de Mello, Horta & Bolfarini; *Guabamima lordelloi* de Mello; *Guabamima saiva* de Mello; *Melanotes ornata* Desutter-Grandcolas; *Ottedana cercalis* de Mello & de Andrade; *Strinatia brevipennis* Chopard; *Strinatia teresopolis* Mesa, and an undescribed species of *Modestozara* Gorochov. *Melanotes ornata* was used to root the trees.

### Characters and data matrix

This study was based on morphological characters of adult males and females, including genitalia. The specimens were examined under stereomicroscope Leica EZ4. The morphological terminology adopted, including the male phallic complex, follows Desutter [36, 55], Desutter-Grandcolas [56], and Souza-Dias [54]. Male phallic complexes were dissected and cleared in an aqueous solution 10% KOH for 24 hours and stored in vial with 80% ethanol together with the respective specimen. The female copulatory papillae were also dissected, but not cleared in KOH, and stored in small vials with ethanol 80% together with the respective specimen. Drawings of the genitalia were made under a Leica MZ9.5 stereomicroscope coupled with camera lucida. The photographs were taken through a Leica MZ16 stereomicroscope with a Leica DFC-420 camera, using the Leica Application Suite LAS V4.0 software, with specimens, male genitalia, and female copulatory papilla immersed in ethanol 80%. Fresh samples were not available in order to complement the phylogeny with molecular data.

The metanotal and forewing glands are important sources of characters in *Eidmanacris* [47, 48, 50, 57]. We used scanning electron microscopy (SEM) to examine the metanotum of 21 species of *Eidmanacris* and six outgroup species. Due to the reduced number of specimens for SEM analysis, the glands of five additional species of *Eidmanacris* and three of outgroup were examined using light microscopy to avoid damaging the specimens. The species studied using SEM are indicated in S1 and S2 Tables. For the SEM analysis, we dissected a male specimen and removed its forewing and thorax. The samples were dehydrated in a graded ethanol series until 100%, critical point dried using CO_2_ as intermediate, mounted on stubs, and coated with gold. The samples were examined using a Scanning Electron Microscope Zeiss SIGMA VP at the Instituto de Biociências da Universidade de São Paulo (Biosciences Institute of the University of São Paulo).

The description of the characters follows Sereno [58] and the data matrix (S3 Table) was constructed in *Mesquite* 3.6 [59]. Inapplicable characters were coded as “-” and unobserved characters as “?”.

Ninety-eight (98) characters (83 binary and 15 multistate) from external morphology were scored, six from the head, 22 from the thorax (including forewings and metanotal gland), six from the abdomen, four from the legs, 54 from male genitalia, and six female exclusive characters (including copulatory papilla). Some of the proposed characters were based on previous studies [54, 60, 61]. However, most characters are proposed here for the first time after a detailed morphological study of *Eidmanacris* following the taxonomic revision of the genus [48]. The list of characters with states, indexes (*ci* and *ri*), comments, optimisations, and illustrations are presented in S1-S18 Figs in the S1 File.

### Phylogenetic analysis

Phylogenetic analyses were carried out in *TNT* 1.5 [62], using TBR as branch swapping with 1000 replications, holding 100 trees per replicate. All characters were treated as unordered with equal weights. A strict consensus tree was constructed in *TNT* whenever the analyses found more than one most parsimonious tree. The resulting trees and patterns of character evolution were visualised in *Winclada* [63], where consistency index (*ci*) [64] and retention index (*ri*) [65] were calculated. Ambiguous characters were optimised using both ACCTRAN [66] and DELTRAN [67]. These optimisations are used to describe the preferences of secondary gains or losses, even though ACCTRAN does not necessarily result in secondary losses and DELTRAN in secondary gains [68, 69]. Sometimes, ACCTRAN may result in illogical optimisations for inapplicable states [68], which happened in some characters in our analysis. Therefore, in this study, both optimisations were applied individually for each character and are indicated when applied. Bremer support values [70] were calculated using the TNT script “BREMER.RUN” to assess branch support.

Two analyses were performed: one with all the 38 terminals studied (analysis 1) and one with 37 terminals (analysis 2), excluding *E*. *paramarmorata*. Known only from females and containing 94% of missing data, this species acts as a “wildcard”, potentially generating big polytomies in consensus trees [71].

### Biogeographical analysis

A total of 61 localities data were obtained from literature [46–48, 50, 51, 72–75], labels of the studied specimens, the catalog *Orthoptera Species File* [52], and from additional material deposited at MZSP and MNHN. All collected data were plotted on a South America map and edited in *QGIS* 3.4 [76]. Biomes (*sensu* Dinerstein et al. [14]) were used for discussion of species distribution patterns and construction of the distribution map. The biomes are: Atlantic Forest (Tropical and Subtropical Moist Broadleaf Forests); Cerrado (Tropical and Subtropical Grasslands, Savannas, and Shrublands); and Chiquitano Dry Forest (Tropical and Subtropical Dry Broadleaf Forests).

Characterization of habitats was based on the nocturnal activity of the species and modified from Desutter-Grandcolas [38]: 1-straminicolous (str), species active in the leaf litter; 2-straminicolous/cavicolous (str/cav), species active in the leaf litter and cavities, including the entrance of caves; 3-cavicolous (cav), species foraging only in cavities at ground level and in the entrance of caves. No troglobitic species (i.e. species that inhabit only the interior of caves) is known so far in *Eidmanacris*. The data were obtained from the same literature of localities [46–48, 50, 51, 72–75], labels of specimens and personal observation in the field (Table 1).

**Table 1:** Distributional, ecological, and morphological characteristics of *Eidmanacris* species.

Clade	Taxa	Biome	Habitat	Size	Metanotum bristles
A	*E*. *fusca*	Atl	str	3	Anterior region
	*E*. *melloi*	Atl	str	1	Anterior region
	*E*. *endophallica*	Atl	str	2	Anterior region
	*E*. *minuta*	Atl	str	0	Anterior region
C	*E*. *bidentata*	Atl	str	1	Anterior region
E	*E*. *putuhra*	Atl	str	1	Anterior region
G	*E*. *tridentata*	Atl	str	1	Anterior region
	*E*. *simoesi*	Atl	str	1	Anterior region
	*E*. *eliethae*	Atl	str	2	Anterior region
I	*E*. *papaveroi*	Atl	str	2	Anterior region
K	*E*. *fontanettiae*	Atl	str	1	Anterior region
M	*E*. *larvaeformis*	Atl	str/cav	4	Anterior region
	*E*. *septentrionalis*	Atl	str/cav	2	Anterior region
	*E*. *speluncae*	Atl	cav	3	Anterior region
	*E*. *multispinosa*	Atl	str	3	Anterior region
N1	*E*. *dissimilis*	Atl/Cer	str/cav	2	Entire metanotum
	*E*. *meridionalis*	Atl	str/cav	2	Entire metanotum
	*E*. *alboannulata*	Atl/Cer	str/cav	3	Entire metanotum
	*E*. *suassunai*	Atl	str/cav	3	Entire metanotum
N2	*E*. *scopula*	Cer	cav	3	Entire metanotum
	*E*. *desutterae*	Cer	str/cav	3	Entire metanotum
	*E*. *corumbatai*	Cer	str/cav	3	Entire metanotum
	*E*. *gigas*	Cer	str/cav	3	Entire metanotum
	*E*. *caipira*	Atl/Cer	str/cav	5	Entire metanotum
	*E*. *bernadii*	Cer	str/cav	4	Entire metanotum
	*E*. *neomarmorata*	Cer	cav	3	Entire metanotum
	*E*. *marmorata*	Chi	str/cav	4	Entire metanotum
	*E*. *longa*	Chi	str/cav	4	Entire metanotum

(Atl) Atlantic Forest; (Cer) Cerrado; (Chi) Chiquitano Dry Forest; (str) straminicolous; (cav) cavicolous; (0) less than 12mm; (1) 12-15mm; (2) 15-18mm; (3) 18–2.

An event-based analysis was performed using the Geographically-explicit Event Model (GEM) method [49]. This method attributes different kinds of events (vicariance, sympatry, founder event, and point sympatry) to internal nodes of the phylogenetic tree applying most of the spatial diversification models implemented in BioGeoBEARS [77], such as DIVA, DEC and DEC+J. However, instead of using pre-defined areas as operational units, GEM considers the geographical coordinates directly, as advocated by Hovenkamp [78], therefore, spurious biogeographical results due to inadequately defined areas may be avoided. The reconstruction cost is calculated as the event cost plus the number of distribution changes along the branches, and the distributions are based on the presence of taxa in distribution grids. The best reconstruction is the reconstruction with the lowest cost. The method was implemented using the software package *EVS* [49].

**Fig 1:**
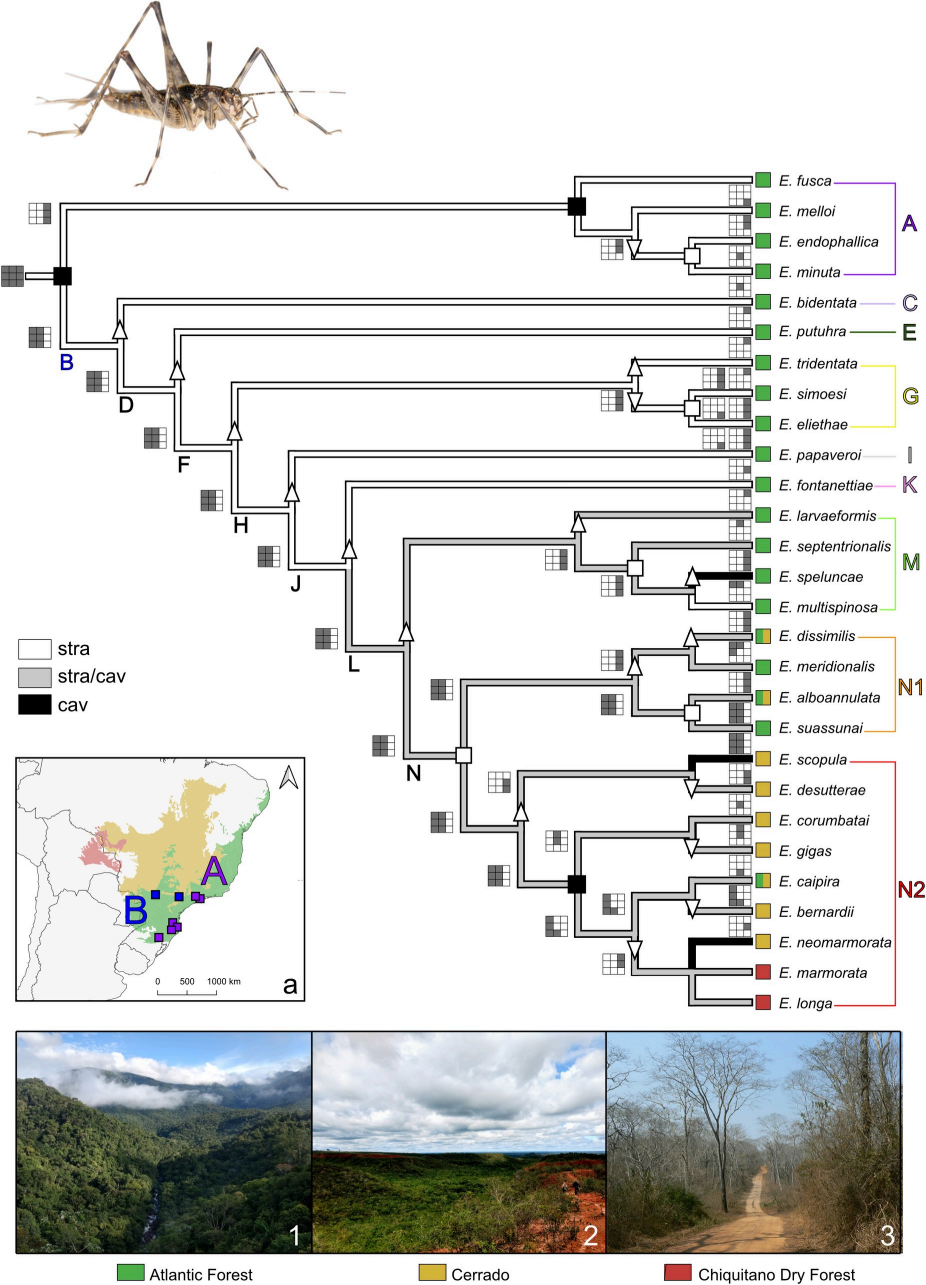
Habitats ancestral state reconstruction of *Eidmanacris* species based on the analysis without *E*. *paramarmorata*. White branches, straminicolous; black branches, straminicolous/cavicolous; grey branches, cavicolous. Phylogeny reconstructed with 98 morphological characters and 28 species of *Eidmanacris*. Symbols on the nodes indicate the events of GEM method: black square, vicariance; white square, sympatry; white triangle, founder event. Squares 3x3 represent the total distribution of *Eidmanacris* in order to illustrate and interpret the sequence of events: grey sub-squares represent the presence and whit sub-squares the absence of species distribution. Colored terminal squares indicate the biomes where species came from: (1) Atlantic Forest, Parque Nacional de Itatiaia; (2) Cerrado, Parque Nacional Grande Sertão Veredas; (3) Chiquitano Dry Forest, Concepción, department of Santa Cruz (Reprinted from Mendivelso et al., 2013 under a CC BY license, with permission from PLOS One, original copyright 2013); (a) ancestral distribution of the genus *Eidmanacris*.

*EVS* was set according to the author’s recommendations: a raster grid with pixels of 1x1 degree was used, with a filling of 1. The cost of all four events (vicariance, sympatry, founder event, and point sympatry) was set to 1. In order to penalize ancestral distributions and avoid widespread ancestors, we used Z = 10 (Z defines the size of the ancestral ranges) as proposed by Arias [49]. The searches were performed using a flipping algorithm with 10 independent runs, each one with 10,000 replicates. The input files are provided in S2 and S3 Files. Permutation tests (10) were done to check the results, as recommended by Arias [49]. After the analysis, 3x3 squares were used in the cladogram to represent hypothetical distributions of the nodes and terminals in order to illustrate and interpret the sequence of events (Fig 1): vicariance events fragment the former ancestral wide distribution into two non-overlapping descendant branches; in sympatry events the two descendant branches have both the same distribution and equal to ancestral node; and in founder event one descendant branch is distributed equal to ancestral node while the other descendant branch has a restricted and non-overlapping distribution (grey sub-squares, distribution present; white sub-squares, distribution absent).

According to Arias [49], vicariance assignment is based on the sum of overlap of each descendant plus the cost of the vicariance, penalizing the overlap to indicate the difficulty of crossing barriers. The cost of assignment of sympatry is the cost of gained and lost pixels of both descendants in relation to the ancestral distribution plus the cost of the event. Point of sympatry assignment is based on the size of the range of overlapped pixels of descendants minus one, plus the event cost, the pixels outside the ancestral range being considered an extra cost. Founder event is based on the size of the range of overlapped pixels of descendants minus one, plus the cost of the event, the overlapped pixels with ancestral ranges being an extra cost (differently of point of sympatry). For detailed information about the method and its application, see Arias [49] and Ruiz et al. [79].

### Morphology and ancestral state reconstruction

Two characters were selected to test the influence of habitats and biomes on *Eidmanacris* biology: body size and the metanotal glands in males. The body size is measured as the length between the fastigium and supra anal plate tip in dorsal view [46, 48]. Both characters were optimised on the phylogeny without *E*. *paramarmorata*.

The size is indicative of resource use [80]. Six non overlapping size categories separated by 3mm each were considered here. The arbitrary division has been chosen to better show size trends onto the topology (Table 1).

The male metanotal glands produce secretions and the females bite the metanotal structure during copulation. These secretions are considered as a male nuptial gift, but they also allow the male to increase mating duration, thus delaying the removal and chewing of the spermatophore by the female [81]. In *Eidmanacris*, the male glandular surface structure is delimited by an anterior median crest, two lateral projections, and a more or less extended area covered with bristles, where the secretions accumulate [48]. To take into account variation in male gland structure (= male investment in mating, or nuptial gift), we consider the surface of the bristle area, with two states: anterior portion of area covered by bristles, and entire area covered by bristles.

Ancestral state reconstructions were performed in *Mesquite 3*.*6* using the parsimony criteria. The reconstructed characters were habitat (3 states), size (6 states) and bristle area in metanotal gland (2 states). The states of reconstructions are in Table 1.

## Results

### Phylogenetic analysis

The cladograms resulting from the analyses with character states and support values are presented in S19 and S20 Figs. The analysis with 37 terminals (including all the species of *Eidmanacris* except *E*. *paramarmorata*, plus all the outgroups) resulted in one most parsimonious tree (S19 Fig), with 240 steps, *ci* = 0.47 and *ri* = 0.78. The analysis with all 38 terminals resulted in 11 most parsimonious trees, with 240 steps, *ci* = 0.47 and *ri* = 0.78. The consensus tree is similar to the topology with 37 terminals, except for a polytomy in clade N2 (S4 File and S20 Fig) as expected with the “wildcard” *E*. *paramarmorata*. The discussion is based on analysis without *E*. *paramarmorata* due the absence of the polytomy of clade N2.

*Eidmanacris* is monophyletic, including the combinations *Eidmanacris endophallica* (de Mello) and *E*. *minuta* (de Mello), as proposed by Campos et al. [48]. All the clades are well supported, including clade N2 (S19 and S20 Figs).

*Eidmanacris* is supported by seven apomorphic and nine homoplastic changes (Table 2 and S4 Table) and the clade receives a high support value (Bremer support = 11). It is divided into two main clades: clade A with four species (*E*. *fusca*, *E*. *melloi*, *E*. *endophallica*, and *E*. *minuta*) and clade B with all the 25 remaining species. Clade B includes eight clades, labelled C to N (composed by N1 and N2) (S19 and S20 Figs), which are all characterised by distinct apomorphies (S5 Table). The clades C, E, I and K each include only one taxon, respectively *E*. *bidentata*, *E*. *putuhra*, *E*. *papaveroi* and *E*. *fontanettiae*.

**Table 2:** Exclusive synapomorphies for the clade *Eidmanacris*.

Character/State	State of character description
32(1)	Presence of latero-posterior projections of the supra anal plate of male
64(1)	Presence of anterior projection of the pseudepiphallic sclerite
67(1)	No sclerotized connection between the PsP1 and PsP2
84(1)	Ectophallic fold membranous
90(1)	Endophallic apodeme crest-shaped
95(1)	Medio-posterior projection of the endophallic sclerite elongate, through all the ectophallic fold
98(1)	Presence of latero-posterior lobes of endophallic sclerite

### Ancestral state reconstructions

Our analyses show that, with 5 steps, the ancestral habitat of *Eidmanacris* is straminicolous, with the species only foraging in the leaf litter. This pattern was modified in clade L becoming straminicolous/cavicolous, *i*.*e*. in addition to being active in the litter, species are also active in cavities and in the entrance of caves. The species *E*. *speluncae* (clade M), *E*. *scopula* and *E*. *neomarmorata* (clade N2) became cavicolous and a subsequent reversal to straminicoly occurs in clade M (*E*. *multispinosa*) (Table 1 and Fig 1).

**Fig 2:**
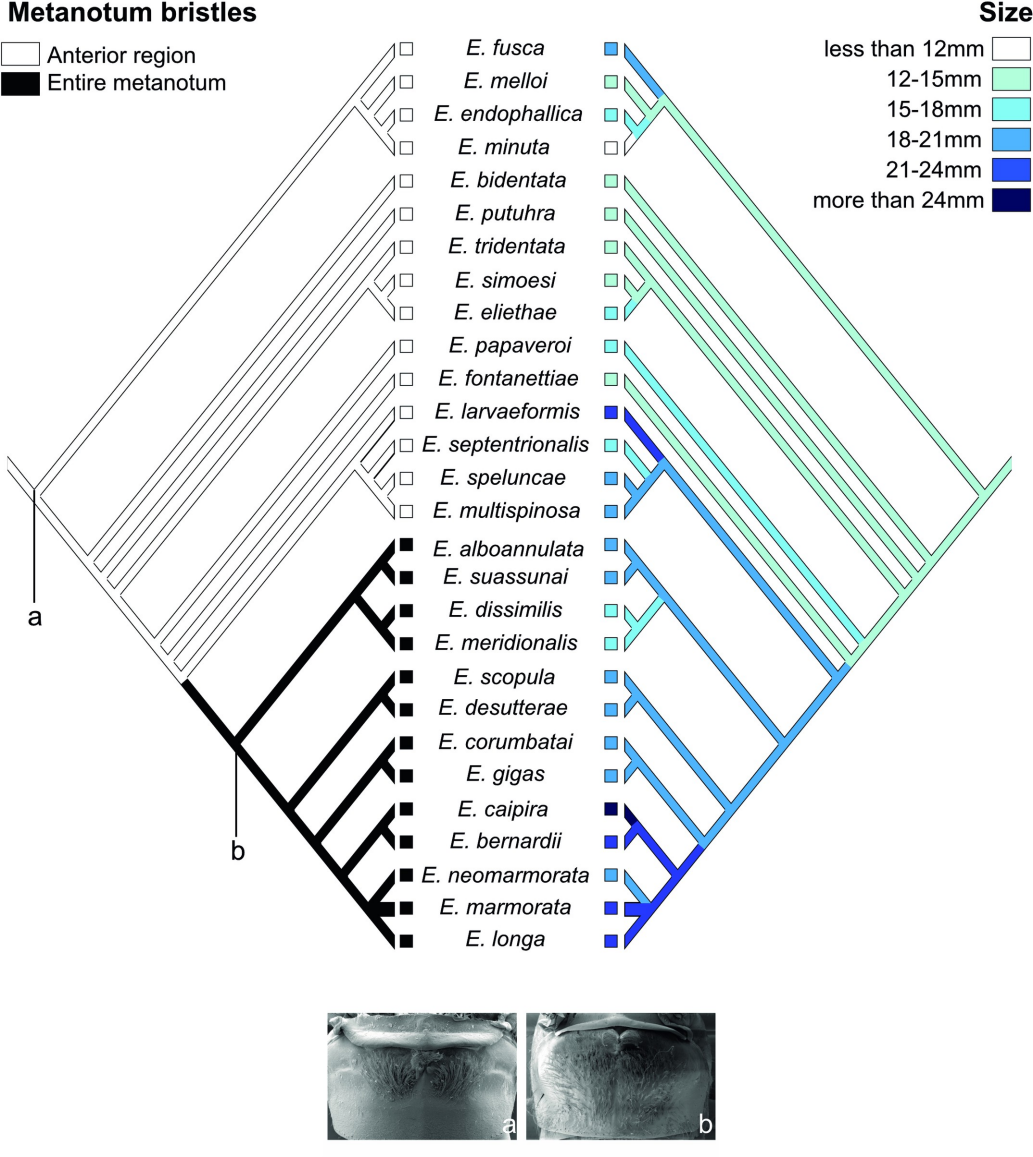
Ancestral state reconstruction of *Eidmanacris* species for bristles on the metanotal gland (left) and size (right).

The reconstruction of size, with 12 steps, displayed a general tendency of size increase along the phylogeny. The ancestral size of the genus is 12-15mm. The size increased in clade L (18-21mm) and the largest species are found in the clade N2 (larger than 21mm) following the same pattern of habitat modification (Table 1 and Fig 2).

A metanotal gland entirely covered by bristles is present in all the species in clade N (Fig 2B), while in the ancestral state of the genus bristles cover only the anterior region of metanotal gland (1 step) (Fig 2A). Species that are found in Cerrado are bigger, and their metanotum is entirely covered by bristles, while species that are only present in the Atlantic Forest, are smaller and have fewer bristles.

### Biogeographical analysis

Applying the GEM method, four reconstructions were returned, two with a cost of 44 and two with a cost of 45. The 45 cost-reconstructions are similar to the 44 cost-reconstructions, except for the direction of a founder event between *E*. *dissimilis* and *E*. *meridionalis* (clade N1). We will discuss here only the 44 cost-reconstructions, which differ only in the node of clade G (a different direction in the founder event). The biogeographic scenario obtained implies three vicariances, five sympatries, and 17 founder events, but no point of sympatry (Fig 1). From the point of view of biome occupancy, the ancestral distribution of *Eidmanacris* was found to be the Atlantic Forest (Fig 1A). Species in clade N2 dispersed to Cerrado but two species, *E*. *marmorata* and *E*. *longa*, colonised the Bolivian Dry forest, and one species, *E*. *caipira*, returned to the Atlantic Forest. *E*. *alboannulata* and *E*. *dissimilis* (clade N1) are found both in Cerrado and Atlantic Forest. The optimisations of size and metanotal glands parallel the changes in the biome occupancy, with species becoming larger and the males having a more extended glandular structure as they colonise drier biomes (Fig 1).

The first vicariance event in the cladogram, in the basal node of *Eidmanacris*, divides the ancestral distribution of the genus (Fig 1A), between clade A and all the remaining species (clade B). A second vicariance event separates *E*. *fusca* of *E*. *melloi* + *E*. *endophallica* + *E*. *minuta* in clade A, with the sister species *E*. *endophallica* and *E*. *minuta* being sympatric. According to the event reconstruction, clade A, together with the species *E*. *alboannulata* and *E*. *suassunai* in clade N1, are present in the ancestral distribution area of the genus (Fig 1A).

As mentioned above, the two 44 cost-reconstructions only differ in the base of clade G. Clade G shows a founder event at its base, between *E*. *tridentata* and two sympatric species, *E*. *simoesi + E*. *eliethae*, but either *E*. *tridentata* or the sympatric *E*. *simoesi* and *E*. *eliethae* could occur in the ancestral distribution of the clade.

According to our results, the ancestral distribution of clade M is different from that of clade B because of the direction of the founder event that occurred in clade L. Clade M and N are separated by a founder event which defines the ancestral distribution of clade N as similar to that of clade B. Clade N is divided by a sympatry, *i*.*e*. clades N1 and N2 have the same ancestral distribution.

**Fig 3:**
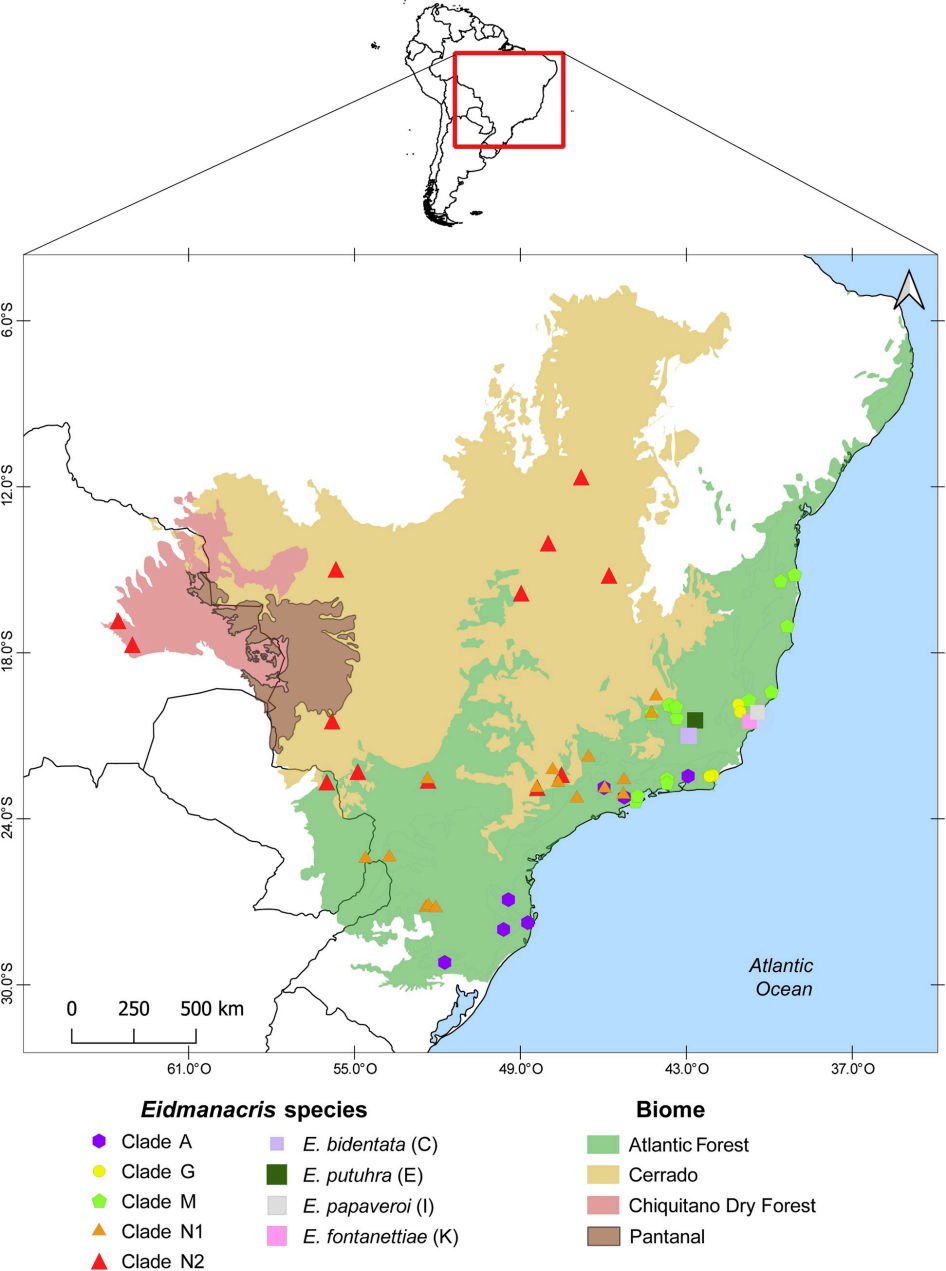
Distribution map of *Eidmanacris* clades and species.

Clade N2 requires five founder events in seven nodes, and a vicariance event in the node separating *E*. *corumbatai + E*. *gigas* and *E*. *caipira + E*. *bernardii + E*. *marmorata + E*. *longa + E*. *neomarmorata* (Fig 3). All the extant distributions of clade N2 species differ from the ancestral distribution of the genus, but *E*. *caipira* is distributed in Atlantic Forest and Cerrado, like species in the clade N1. The other taxa of clade N2 have a western distribution, either in the Cerrado of central Brazil or in the Chiquitano Dry Forest from Bolivia (*E*. *longa* and *E*. *marmorata*).

Through the whole clade, sympatries are observed, either between species belonging to different clades (e.g., *E*. *tridentata*, *E*. *papaveroi*, and *E*. *multispinosa*) or between sister species (e.g., *E*. *minuta* and *E*. *endophallica*).

## Discussion

*Eidmanacris*, with 29 species, is one of the most species rich genera of crickets from the Neotropical region [52], and the first in which a well-supported phylogeny has been constructed for all known species, following intensive field surveys. Our phylogenetic study confirms the monophyly of the genus, provided it includes *Endophallusia* de Mello, as proposed earlier [48]. The genus is subdivided into nine clades with strong support for all the nodes in the resultant cladogram (S19 and S20 Figs).

### Ancestral distribution and diversification

The distribution of the *Eidmanacris* species covers the south, southeast, and center-west Brazil, as well as eastern Paraguay and eastern Bolivia, which corresponds to the Atlantic Forest, Cerrado, and the Chiquitano Dry Forest regions. Further cricket surveys in South America show that *Eidmanacris* is absent in the entire Amazonian region, as well as the southeastern and southern Atlantic Forest and southern South America. Using GEM, the ancestral distribution of the genus is hypothesised to have been the Atlantic Forest region, where *Eidmanacris* had its ancient diversification (Fig 1A). Only the more posterior clades have expanded the distribution of the genus further west, within the Brazilian central region to Bolivia.

This distribution pattern documents several biome changes, from the Atlantic Forest to the dryer biomes of Cerrado and the Chiquitano Dry Forest. Within Cerrado, *Eidmanacris* has been found only in gallery forests and never in other physiognomies, e.g. Campo limpo, Campo sujo, and Cerradão, although several collecting trips have been conducted to find them (LDC, pers. obs.). As mentioned above, gallery forests are known to act as remnant humid forests in dry and open regions and as dispersal corridors, allowing forest-dwelling species to reach the savanna areas [82]. This pattern of distribution is also documented for plants [18, 20, 21], butterflies [21, 83, 84], anurans [85], lizards [86], mammals [24, 87] and birds [26, 88].

Studies on mammals from Cerrado also suggest that gallery forests provide habitats for distinct communities throughout the Cerrado, and that these environments favor Cerrado colonisation by both Atlantic and Amazon forests species [24, 89].

According to our phylogenetic and biogeographic results, *Eidmanacris* originated in the Atlantic Forest, dispersed to Cerrado gallery forests, and from there colonised the Chiquitano Dry Forests in eastern Bolivia; some lineages returned to the Atlantic Forest, again through Cerrado gallery forests. However, *Eidmanacris* did not reach the Amazon Rainforest through Cerrado gallery forests. Even though *Eidmanacris* species from clade N are found in regions with environmental conditions different from Atlantic Forest, they are active in places that retain humidity, such as caves, rock crevices, and cavities near rivers that are mainly associated with gallery forests [47, 48, 50].

Two non-excludent hypotheses may explain how these species may have colonised the Cerrado and Bolivian Dry Forests:

1. *Eidmanacris* was present in Cerrado and forced into a refuge in gallery forests by climatic variations: The climatic changes that favored vegetations with dryer characteristics during the Miocene and Pliocene could have contributed to a distributional retraction of *Eidmanacris* species to the gallery forests (and any forest refuges) of the Cerrado and Chiquitano Dry Forest [90–92].
2. *Eidmanacris* species were not present in the region of Cerrado and Chiquitano Dry Forest prior to the expansion of the xeric vegetation in the Quaternary period but may have colonised these biomes by dispersal through the gallery forests in more recent times. Palynological records showed that after the humidity increased again, an expansion of humid forests occurred in the Cerrado [93] allowing species of Atlantic Forest to expand into the Cerrado through gallery forests and stational forests [94].

Without a dated phylogeny, it is not possible to support any alternative. However, after xeric vegetations were established, the species may have used the gallery forests as dispersal corridors as well as refuges, which supports the idea that speciation and diversification in the Neotropics cannot be explained by only a single model of vicariance or climatic changes [29, 95]. Thus, the species would have colonised other regions in Cerrado and had the possibility to return to the Atlantic Forest, as did *E*. *caipira*. This is supported by the observations that two species in clade N1, *E*. *dissimilis* and *E*. *alboannulata*, are present both in the Atlantic Forest and in Cerrado, in geographically very approximate localities (Fig 3). In Cerrado, however, both species were collected only in cave entrances. The microclimate inside cavities and caves could maintain conditions of humidity and temperature close to that found in humid forests, and so allow the permanent presence of *Eidmanacris* in these dryer biomes.

The non-occurrence of *Eidmanacris* species in the Amazon Rainforest (LDC and LDG, pers. obs.; [52]) could fit the hypothesis of altitudinal segregation of fauna and flora species between Amazon and Atlantic Forests [26]. Amazonian elements occur at lower altitudes than the Atlantic elements, and more Atlantic Forest species occur in higher altitudes of Cerrado than in the Amazon area [26]. According to Oliveira-Filho & Fontes [96], Cerrado is more influenced by Atlantic Forest than by the Amazon Rainforest, mainly because the Atlantic Forest climate is more similar to that of Cerrado, with lower temperatures and pronounced dry seasons in the winter [97]. Furthermore, in the Amazon we found *Phalangopsis* Serville, which occupies the same habitat as *Eidmanacris* and does not occur in the Atlantic Forest, Cerrado, and Bolivian Dry Forest [52]. Similarly, *Strinatia* Chopard occurs in southern and southeastern Atlantic Forest areas where *Eidmanacris* does not occur, while both occupy the same habitat. These mutually exclusive distributional areas between closely-related phalangopsid genera with similar habits, *Eidmanacris* and *Phalangopsis* on one hand, *Eidmanacris* and *Strinatia* on the other, may represent cases of ecological vicariance [98], a question which will have to be further investigated to increase our understanding of cricket diversification patterns in the Neotropics.

A founder event occurs when a new population is established from a few individuals of an ancestral population [99, 100], leading to a founder effect, often documented in population genetics on speciation and island colonization, e.g. [101–103]. Here, the number of founder events in the phylogeny suggests that *Eidmanacris* colonised new regions over time based on dispersals especially in clade N in which seven founder events occur in ten nodes. This scenario may have been supported by gallery forests within the Cerrado.

Among the three vicariance events recorded on *Eidmanacris* phylogeny, two are related to the presence of clade A species in mountain ranges and one is related to gallery forests. The species in clade A occur in forests at the slopes of Serra do Mar, Serra dos Orgãos and Serra Geral. According to Haffer [104], refuges in the Atlantic Forest region were probably located on mountain range slopes during dry periods maintained by orographic rainfalls. A basal vicariance event occurred between *E*. *fusca*, which is restricted to Serra Geral mountain range (state of Santa Catarina and northeastern of state of Rio Grande do Sul, Brazil) and its sister group *E*. *melloi + E*. *endophallica + E*. *minuta* that occurs in Serra do Mar, northeastern of São Paulo State and Serra dos Orgãos, in state of Rio de Janeiro state respectively.

The sympatric distributions observed in *Eidmanacris* occur either between species belonging to different clades or between sister species. Sympatry is usually accompanied by differences between species so that interspecific hybridization or overlap in resource use are avoided. Among the characters we studied, metanotal structures (related to mating behaviour) and size (related to resource use) can influence species interactions. The sister species *E*. *endophallica* and *E*. *minuta* (clade A), which occur together in the litter at the Parque Nacional da Serra dos Orgãos, state of Rio de Janeiro, are distinct morphologically: *E*. *endophallica* has no metanotal structures and a mean size of 16mm, while *E*. *minuta* is only 11mm in length (the smallest species of the genus) and has glandular structures in the metanotum. The morphological divergences of both species likely interfere directly on their mating behavior and resource use.

A similar situation exists between the species *E*. *tridentata*, *E*. *papaveroi* and *E*. *multispinosa*, which belong to clades G, I and M of *Eidmanacris* (Fig 1) and are sympatric in an Atlantic Forest fragment in Santa Teresa, state of Espírito Santo: the three species differ in size (see measurements in [46, 48]; Table 1) and in metanotal morphology (glandular structures absent in *E*. *tridentata*; vestigial in *E*. *papaveroi*; complete in *E*. *multispinosa*).

### Habitat, resources, and behaviour changes

As a whole, the phylogeny illustrates a very coherent pattern of evolution for biome occupancy, habitat, resource use and mating behaviour in *Eidmanacris*. During the diversification of the genus, species became more straminicolous/cavicolous rather than only straminicolous, thus shifting toward more humid habitats, and species size increased significantly as they conquered dryer biomes; male nuptial gifts also increased, consequently allowing longer mating duration.

The changes in habitat and biome occupancy co-occur. Straminicolous species are present in the Atlantic Forest, while straminicolous/cavicolous or cavicolous species are found in the Atlantic Forest, but also Cerrado and the Chiquitano Dry Forest. For example, *E*. *endophallica* (clade A) is straminicolous in the Atlantic Forest; *E*. *larvaeformis* (clade M), *E*. *bernardii* (clade N2), and *E*. *marmorata* (clade N2) are straminicolous/cavicolous in the Atlantic Forest, Cerrado and the Chiquitano Dry Forest respectively; *E*. *speluncae* (clade M) and *E*. *neomarmorata* (clade N2) are cavicolous in the Atlantic Forest and Cerrado (Fig 1).

The changes in preferred habitats also follows the tendency of size increase within the genus (Table 1, Fig 2). Different resources are available in plain forest *versus* gallery forest, and in leaf litter *versus* cavities. Larger size could mean different/more available resources for the straminicolous/cavicolous species inside gallery forests or caves in Cerrado and the Chiquitano Dry Forest, compared to straminicolous species in humid forests. No data on *Eidmanacris* diet is available to explain these observations. Furthermore, larger size could also mean better resistance to desiccation [105, 106], through a greater capacity to store water [107] and reduced water loss with a smaller surface area/volume ratio [108].

With respect to mating behaviour, the modification in the metanotal gland is the most evident change in clade N, with bristles occupying the whole metanotum surface (Fig 2). While the female feeds on the nuptial gift, the male couples the genitalia in the female copulatory papilla and attaches the spermatophore tube [109]. More secretions in the bristles entertain the female for a longer time, increasing the male success to transfer its sperm [47, 110]. Clade N2 is also the most divergent in the genus: it is defined by seven apomorphies, six of which are related to reproduction, i.e. five related to male genitalia, and one related to aperture of the female copulatory papilla (see S5 Table). These modifications could interfere with the attachment of the male phallic complex in the female copulatory papilla, also modifying how the spermatophore tube is coupled in the female genitalia. The modifications of the metanotal gland, and male and female genitalia indicate that the mating behaviours of clade N2 species probably differ from the other species of the genus present in the Atlantic Forest.

Thus, *Eidmanacris* managed to occupy drier places by shifting habitats, becoming larger and changing mating behaviour.

### Conservation issues

Conservation issues in the Atlantic Forests are becoming of crucial importance, as stated by several authors [33, 96, 111, 112]. The Atlantic Forest is one of 34 hotspots for conservation globally, and one of the most threatened [113]. According to Ribeiro et al. [114], only 11.73% of the original vegetation in the Atlantic Forest remains preserved, while more than 50% of animals and plants are endemic [113].

For harvestmen (Opiliones), twelve areas of endemism have been identified in the Atlantic Forest [115]. *Eidmanacris*, which has similar affinities to moist forests and humid microhabitats, occurs in seven of these areas, namely Bahia, Espírito Santo, Serra da Mantiqueira, Serra dos Orgãos, Southern Rio de Janeiro coast, Serra do Mar of São Paulo and Santa Catarina. It is absent in five endemism areas, three areas poorly sampled for crickets (Pernambuco, Serra do Espinhaço, Serra da Bocaina), and two areas further south, which are occupied by the genus *Strinatia* (Southern of São Paulo, Parana, see above).

The similar patterns of occurrence of *Eidmanacris* and harvestmen in the Atlantic Forest, highlight the need for biogeographical studies using non model organisms and consequently further enhancing the conservation policies of this biome. The pattern of distribution of *Eidmanacris* species, although documented by few localities, increases the originality of the fauna in the Atlantic Forest and supports the same core areas as for harvestmen.

Similarly, gallery forests play an important role for species from the Atlantic Forest in Cerrado, as illustrated by *Eidmanacris* species in clade N, but also for other species of invertebrates [21, 83], vertebrates [24, 26, 29] and plants [20, 23, 116]. These forests increase the biodiversity of the Cerrado biome, acting as a secondary habitat for organisms [29, 82]. Nevertheless, Cerrado and its gallery forests are threatened by human activities, such as cattle raising and agricultural expansion in the central region of Brazil [117–119]. In addition to their function as dispersal corridors and their general ecological role, the gallery forests play a key role in the preservation of the environment, stabilising soils and protecting rivers and its headwaters [120].

## Conclusion

The phylogenetic patterns of *Eidmanacris* recovered here, with congruent changes in distribution, habitat, resource use and mating behaviour, shows the fragile balance that may allow species survival and dispersal. This case study clearly highlights the need to protect what remains of these Neotropical biomes, and their interactions in terms of taxa survival and evolution. In the future, additional geographical and ecological data per species will allow niche reconstructions [121, 122], complementing direct observations on habitats and nychthemeral activity, in order to further document the criteria of distribution of *Eidmanacris* species. Also, additional behavioural observations, the relations between biome occupancy, habitat, resource use to further analyses could reinforce the hypotheses on the motor of the evolution of *Eidmanacris* and future conservation issues.

## Supporting information

S1 Fig **A**- Shape of median ocellus in frontal view: **a**- elliptical, **b-** spherical, **c-** inferiorly truncated; **B**- Frontal head: **a**-*Strinatia teresopolis*, **b**-*Guabamima lordelloi*, **c**-*Eidmanacris larvaeformis*; **C**- Palpus, 5^th^ article: **a**-*Eidmanacris meridionalis*, **b-***Adenopygus heikoi*, **c**-*Eidmanacris endophallica*, **d-***Strinatia teresopolis*; **D**- Apex of forewings, in dorsal view: **a**-rounded, **b**-squared, **c**-triangular; **E**- Male metanotum, dorsal: **a-***Eidmanacris larvaeformis*, **b-***Eidmanacris simoesi*.
(TIF)

S2 Fig **A**- Lateral projections of males metanotal gland: **a**-*Eidmanacris meridionalis*, **b**-*Eidmanacris septentrionalis*; **B**- Metanotum, dorsal **a**-*Eidmanacris caipira*, **b**-*Adenopygus heikoi*; **C**-*Eidmanacris meridionalis*: **a**-antero-median crest and lateral projections, **b-**median projection; **D**- Metanotum, dorsal: **a**-*Strinatia brevipennis*, **b**-*Eidmanacris melloi*.
(TIF)

S3 Fig **A**- *Eidmanacris scopula*, males metanotum dorsal; **B-** *Eidmanacris endophallica*, abdomen dorsal; **C**- Supra anal plate: **a-***Eidmanacris dissimilis*, **b-***Eidmanacris alboannulata;* **D-** Subgenital plate, posterior border: **a-***Eidmanacris fusca*, **b-***Eidmanacris larvaeformis*; **E-** Apical spurs of tibia II; **F-** Apical spurs of tibia III: **a-** dorsal longer than median, **b-**median longer than median, **c-** dorsal and median sub-equals.
(TIF)

S4 Fig **A**- Female, tegmina: **a-***Melanotes ornata*, **b-***Eidmanacris suassunai*; **B**- Female, median invagination of subgenital plate: **a**-only at the posterior border, **b**-close or reaching the median part; **C**-Apex of ovipositor: **a**- pointed, **b**- straight, **c**- curved; **D-**Copulatory papilla, dorsal: **a-***Eidmanacris septentrionalis*, **b-***Eidmanacris gigas*; **E-** *Strinatia teresopolis*, copulatory papilla, dorsal. **F-** Male, forewing, lateral view: **a-***Melanotes ornata*, **b-***Eidmanacris dissimilis*; **G-** *Eidmanacris meridionalis*, apex of forewing, ventral view; **H-** Forewings, dorsal view: **a-** *Melanotes ornata*, **b-** *Eidmanacris desutterae*, **c-** *Eidmanacris simoesi*.
(TIFF)

S5 Fig **A-** *Melanotes ornata*, phallic complex dorsal; **B**- Phallic complex, dorsal: **a**-*Eidmanacris multispinosa*, **b**-*Eidmanacris tridentata*; **C-** Phallic complex, lateral: **a**- *Eidmanacris bernardii*, **b**-*Ottedana cercalis*, **c**-*Eidmanacris corumbatai*; **D-** Pseudepiphallic sclerite, dorsal: **a**-*Eidmanacris simoesi*, **b**-*Eidmanacris fusca*; **E-***Adenopygus heikoi*, dorsal phallic complex; **F**-Pseudepiphallic sclerite, lateral: **a**- *Eidmanacris larvaeformis*, **b-***Eidmanacris simoesi*.
(TIFF)

S6 Fig **A-** *Eidmanacris corumbatai*, apex of pseudepiphallic arm: **a**- inner side, **b**-outer side; **B-** Phallic complex dorsal: **a**-*Eidmanacris dissimilis*, **b-***Eidmanacris larvaeformis*; **C-** PsP2 and pseudepiphallic arms, dorsal: **a-***Guabamima saiva*, **b**-*Eidmanacris papaveroi*; **D**- Phallic complex, ventral: **a-***Eidmanacris dissimilis*, **b-** *Bambuina bambui*.
(TIF)

S7 Fig **A-** Phallic complex ventral: **a**-*Eidmanacris fontanettiae*, **b**-*Eidmanacris alboannulata*; **B-** Phallic complex, lateral: a-*Eidmanacris alboannulata*, **b-***Eidmanacris desutterae*; **C-** Phallic complex, dorsal: **a-***Eidmanacris multispinosa*, **b**-*Eidmanacris eliethae*, **c-***Eidmanacris bernardii*; **D-** Phallic complex, dorsal: **a**-*Strinatia brevipennis*, **b**-*Eidmanacris septentrionalis*.
(TIFF)

S8 Fig **A-** Phallic complex, dorsal: **a**-*Eidmanacris suassunai*, **b**-*Eidmanacris scopula*.; **B-***Eidmanacris melloi*, subapical margins of ectophallic fold; **C-** Phallic complex, ventral: **a**-*Eidmanacris larvaeformis*, **b**-*Eidmanacris multispinosa*, **c**-*Eidmanacris caipira*; **D-** Dorsal projection of ectophallic invagination, posterior border: **a**- only on posterior border, **b-**reaching median region, **c**-no concavity.
(TIF)

S9 Fig **A-** *Eidmanacris gigas*, endophallus: **a**-dorsal, **b**-ventral, **c**-lateral; **B-** *Guabamima lordelloi*, phallic complex ventral; **C-** *Eidmanacris minuta*, endophallic apodeme lateral; **D-** Endophallus, ventral: **a**-*Eidmanacris speluncae*, **b**-*Eidmanacris alboannulata*, **c**-*Eidmanacris meridionalis*.
(TIF)

S10 Fig Projections of apex of pseudepiphallic arm. *E*. *larvaeformis*: **A**- dorsal, **B**- inner side, **C**- outer side, **D**- ventral; *E*. *alboannulata*: **E**- dorsal, **F**- inner side, **G**- outer side, **H**- ventral; *E*. *septentrionalis*: **I**- dorsal, **J**- inner side, **K**- outer side, **L**- ventral; *E*. *tridentata*: **M**- dorsal, **N**- inner side, **O**- outer side, **P**- ventral. Projections: 1- superior, 2- supero-internal, 3- infero-internal, 4- inferior. Scale bar: 1 mm.
(TIF)

S11 Fig Projections of apex of pseudepiphallic arm. *E*. *multispinosa*: **A**- dorsal, **B**- inner side, **C**- outer side, **D**- ventral; *E*. *dissimilis*: **E**- dorsal, **F**- inner side, **G**- outer side, **H**- ventral; *E*. *meridionalis*: **I**- dorsal, **J**- inner side, **K**- outer side, **L**- ventral; *E*. *fusca*: **M**- dorsal, **N**- inner side, **O**- outer side, **P**- ventral. Projections: 1- superior, 2- supero-internal, 3- infero-internal, 4- inferior. Scale bar: 1 mm.
(TIF)

S12 Fig Projections of apex of pseudepiphallic arm. *E*. *bidentata*: **A**- dorsal, **B**- inner side, **C**- outer side, **D**- ventral; *E*. *corumbatai*: **E**- dorsal, **F**- inner side, **G**- outer side, **H**- ventral; *E*. *suassunai*: **I**- dorsal, **J**- inner side, **K**- outer side, **L**- ventral. Projections: 1- superior, 2- supero-internal, 3- infero-internal, 4- inferior, 5- ventral. Scale bar: 1 mm.
(TIF)

S13 Fig Projections of apex of pseudepiphallic arm. *E*. *caipira*: **A**- dorsal, **B**- inner side, **C**- outer side, **D**- ventral; *E*. *bernardii*: **E**- dorsal, **F**- inner side, **G**- outer side, **H**- ventral; *E*. *papaveroi*: **I**- dorsal, **J**- inner side, **K**- outer side, **L**- ventral. Projections: 1- superior, 2- supero-internal, 3- infero-internal, 4- inferior. Scale bar: 1 mm.
(TIF)

S14 Fig Projections of apex of pseudepiphallic arm. *E*. *simoesi*: **A**- dorsal, **B**- inner side, **C**- outer side, **D**- ventral; *E*. *eliethae*: **E**- dorsal, **F**- inner side, **G**- outer side, **H**- ventral; *E*. *scopula*: **I**- dorsal, **J**- inner side, **K**- outer side, **L**- ventral. Projections: 1- superior, 2- supero-internal, 3- infero-internal, 4- inferior. Scale bar: 1 mm.
(TIF)

S15 Fig Projections of apex of pseudepiphallic arm. *E*. *desutterae*.: **A**- dorsal, **B**- inner side, **C**- outer side, **D**- ventral; *E*. *gigas*: **E**- dorsal, **F**- inner side, **G**- outer side, **H**- ventral; *E*. *putuhra*: **I**- dorsal, **J**- inner side, **K**- outer side, **L**- ventral. Projections: 1- superior, 2- supero-internal, 3- infero-internal, 4- inferior, 5- ventral. Scale bar: 1 mm.
(TIF)

S16 Fig Projections of apex of pseudepiphallic arm. *E*. *neomarmorata*: **A**- dorsal, **B**- inner side, **C**- outer side, **D**- ventral; *E*. *fontanettiae*: **E**- dorsal, **F**- inner side, **G**- outer side, **H**- ventral; *E*. *melloi*: **I**- dorsal, **J**- inner side, **K**- outer side, **L**- ventral; *E*. *speluncae*: **M**- dorsal, **N**- inner side, **O**- outer side, **P**- ventral. Projections: 1- superior, 2- supero-internal, 3- infero-internal, 4- inferior. Scale bar: 1 mm.
(TIF)

S17 Fig Projections of apex of pseudepiphallic arm. *E*. *endophallica*: **A**- dorsal, **B**- inner side, **C**- outer side, **D**- ventral; *E*. *minuta*: **E**- dorsal, **F**- inner side, **G**- outer side, **H**- ventral; *Strinatia brevipennis*: **I**- dorsal, **J**- inner side, **K**- outer side, **L**- ventral; *Strinatia teresopolis*: **M**- dorsal, **N**- inner side, **O**- outer side, **P**- ventral. Projections: 1- superior, 2- supero-internal, 3- infero-internal, 4- inferior. Scale bar: 1 mm.
(TIF)

S18 Fig Projections of apex of pseudepiphallic arm. *Ottedana cercalis*: **A**- dorsal, **B**- inner side, **C**- outer side, **D**- ventral; *Bambuina bambui*: **E**- dorsal, **F**- inner side, **G**- outer side, **H**- ventral; *Adenopygus heikoi*: **I**- dorsal, **J**- inner side, **K**- outer side, **L**- ventral; *Guabamima lordelloi*: **M**- dorsal, **N**- ventral; *Guabamima saiva*: **O**- dorsal, **P**- ventral; *Melanotes ornata*: **Q**- dorsal, **R-** Projections: 1- superior, 2- supero-internal, 3- infero-internal, 4- inferior. Scale bar: 1 mm.
(TIF)

S19 Fig Most parsimonious tree without *E. paramarmorata* (L, 240; *ci*, 0.47; *ri*, 78) from the cladistic analysis of morphological characters, under equal weights, characters optimised, and branch support values. White circles indicate homoplastic synapomorphies, black circles exclusive synapomorphies. Number above circle indicates the characters, above the states. Bremer support are between brackets.
(TIF)

S20 Fig Strict consensus tree of 11 most parsimonious trees including *E*. *paramarmorata* (L, 240; *ci*, 0.47; *ri*, 78) from the cladistic analysis of morphological characters, under equal weights, characters optimised, and branch support values. White circles indicate homoplastic synapomorphies, black circles exclusive synapomorphies. Number above circle indicates the characters, above the states. Bremer support are between brackets.
(TIF)

S1 File List of characters. (DOCX)

S2 File GEM input file: Trees. (TAB)

S3 File GEM input file: Records. (TAB)

S4 File Analysis with *E*. *paramarmorata*, results. (DOCX)

S1 Table Material examined for the ingroup taxa included in the phylogenetic analysis. (DOCX)

S2 Table Material examined for outgroup taxa included in the phylogenetic analysis. (DOCX)

S3 Table Data matrix: Terminals and characters. (DOCX)

S4 Table Homoplastic synapomorphies of *Eidmanacris*. (DOCX)

S5 Table Exclusive synapomorphies of the clades A to N of *Eidmanacris*. (DOCX)

S1 Original figure (TIF)
