# Supplementary figures and images for: Colonization of different biomes drove the diversification of the Neotropical Eidmanacris crickets (Insecta: Orthoptera: Grylloidea: Phalangopsidae)
Source: PLoS One. 2021 Jan 15;16(1):e0245325. doi: 10.1371/journal.pone.0245325 (PMC7810296; doi:10.1371/journal.pone.0245325)

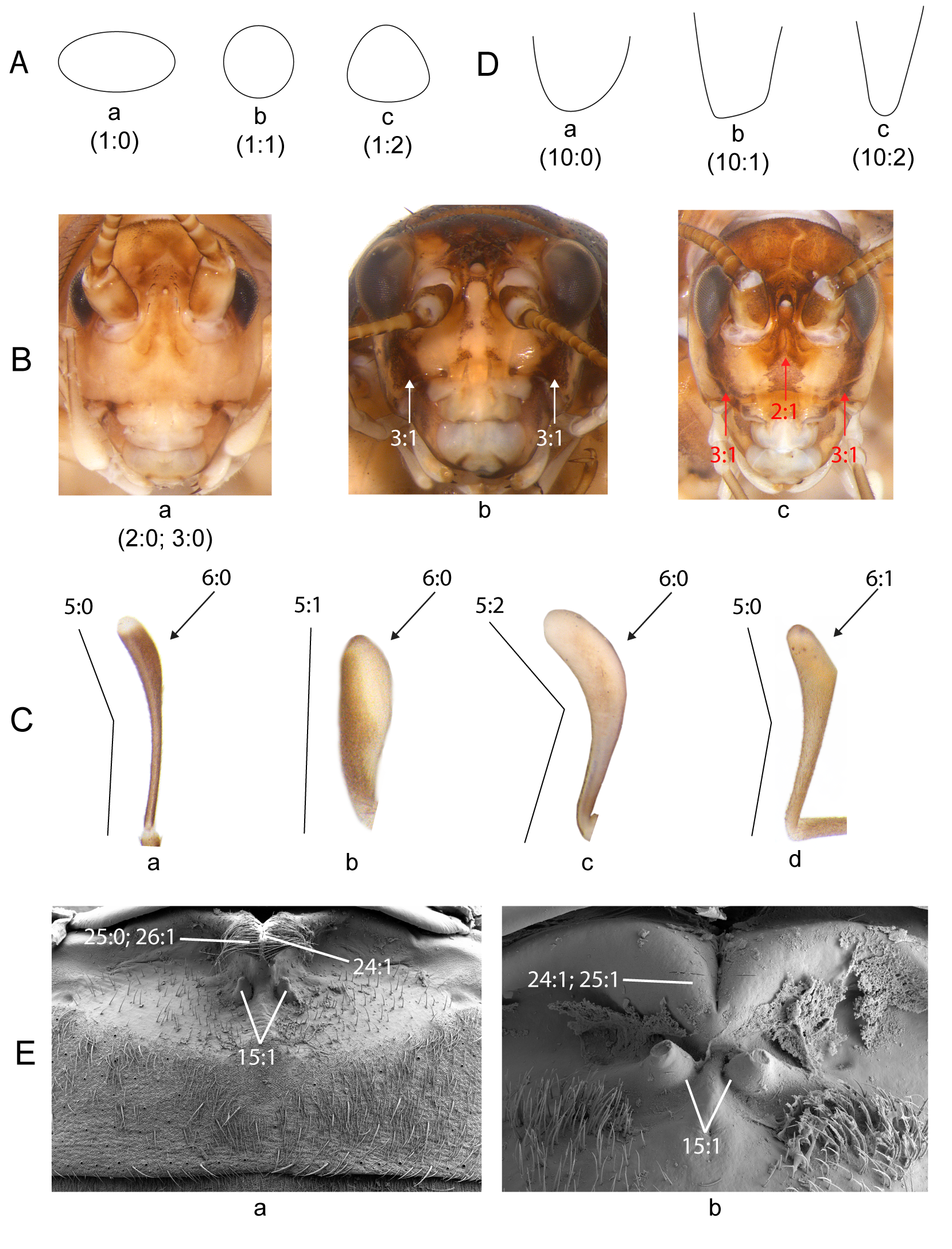

Supplement: S1 Fig — A- Shape of median ocellus in frontal view: a- elliptical, b- spherical, c- inferiorly truncated; B- Frontal head: a-Strinatia teresopolis, b-Guabamima lordelloi, c-Eidmanacris larvaeformis; C- Palpus, 5th article: a-Eidmanacris meridionalis, b-Adenopygus heikoi, c-Eidmanacris endophallica, d-Strinatia teresopolis; D- Apex of forewings, in dorsal view: a-rounded, b-squared, c-triangular; E- Male metanotum, dorsal: a-Eidmanacris larvaeformis, b-Eidmanacris simoesi. (TIF) [file pone.0245325.s001.tif]

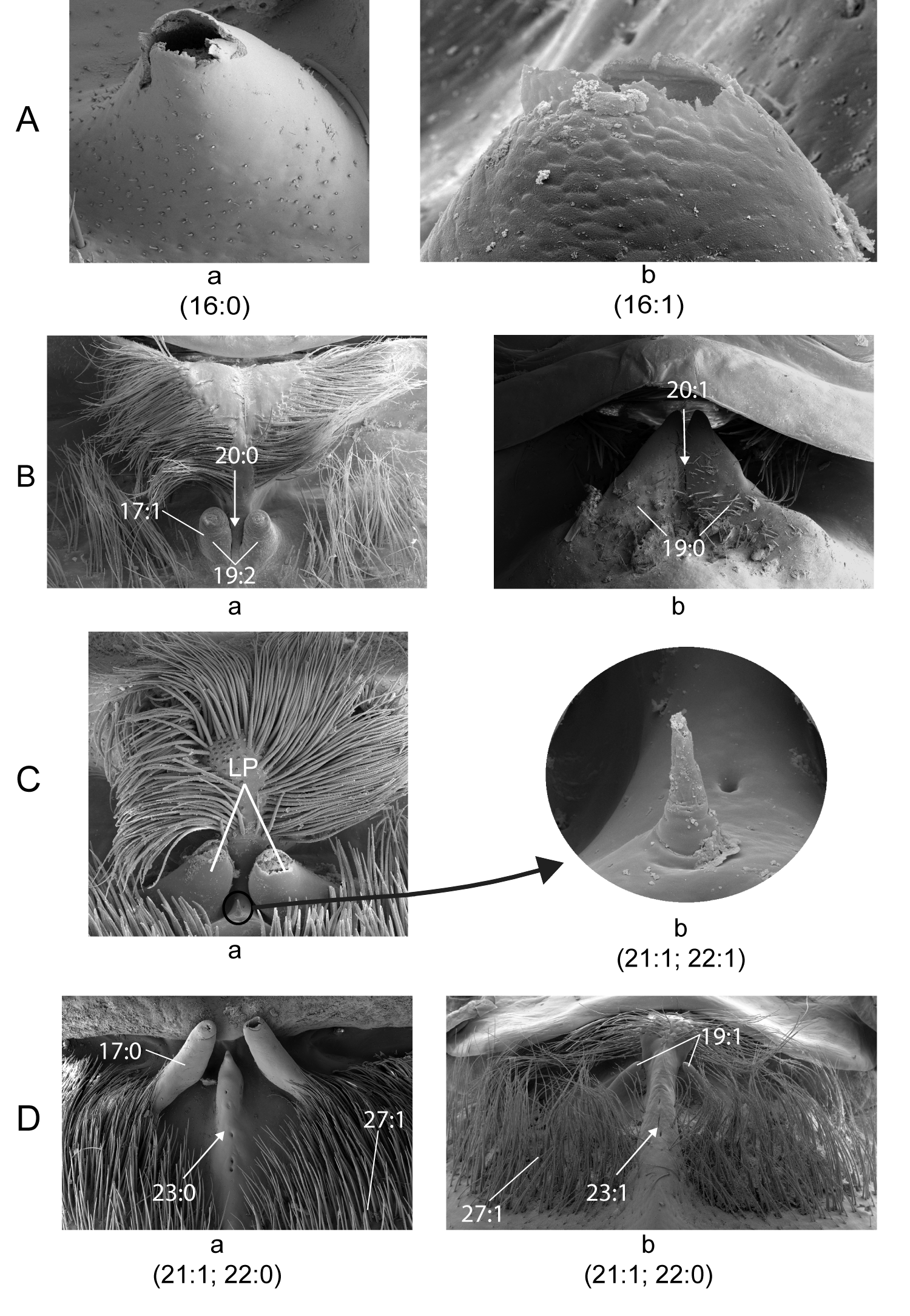

Supplement: S2 Fig — A- Lateral projections of males metanotal gland: a-Eidmanacris meridionalis, b-Eidmanacris septentrionalis; B- Metanotum, dorsal a-Eidmanacris caipira, b-Adenopygus heikoi; C-Eidmanacris meridionalis: a-antero-median crest and lateral projections, b-median projection; D- Metanotum, dorsal: a-Strinatia brevipennis, b-Eidmanacris melloi. (TIF) [file pone.0245325.s002.tif]

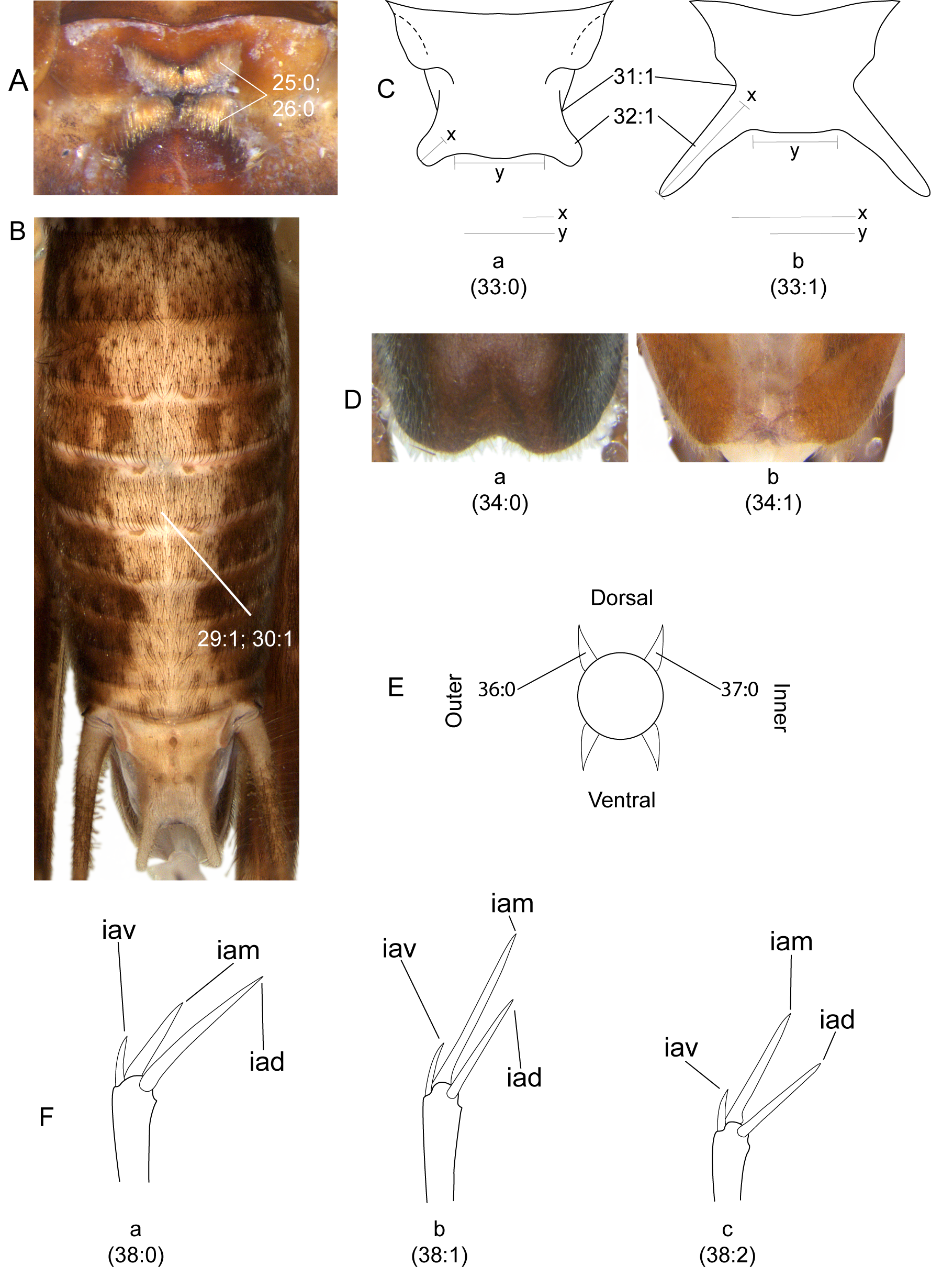

Supplement: S3 Fig — A- Eidmanacris scopula, males metanotum dorsal; B- Eidmanacris endophallica, abdomen dorsal; C- Supra anal plate: a-Eidmanacris dissimilis, b-Eidmanacris alboannulata; D- Subgenital plate, posterior border: a-Eidmanacris fusca, b-Eidmanacris larvaeformis; E- Apical spurs of tibia II; F- Apical spurs of tibia III: a- dorsal longer than median, b-median longer than median, c- dorsal and median sub-equals. (TIF) [file pone.0245325.s003.tif]

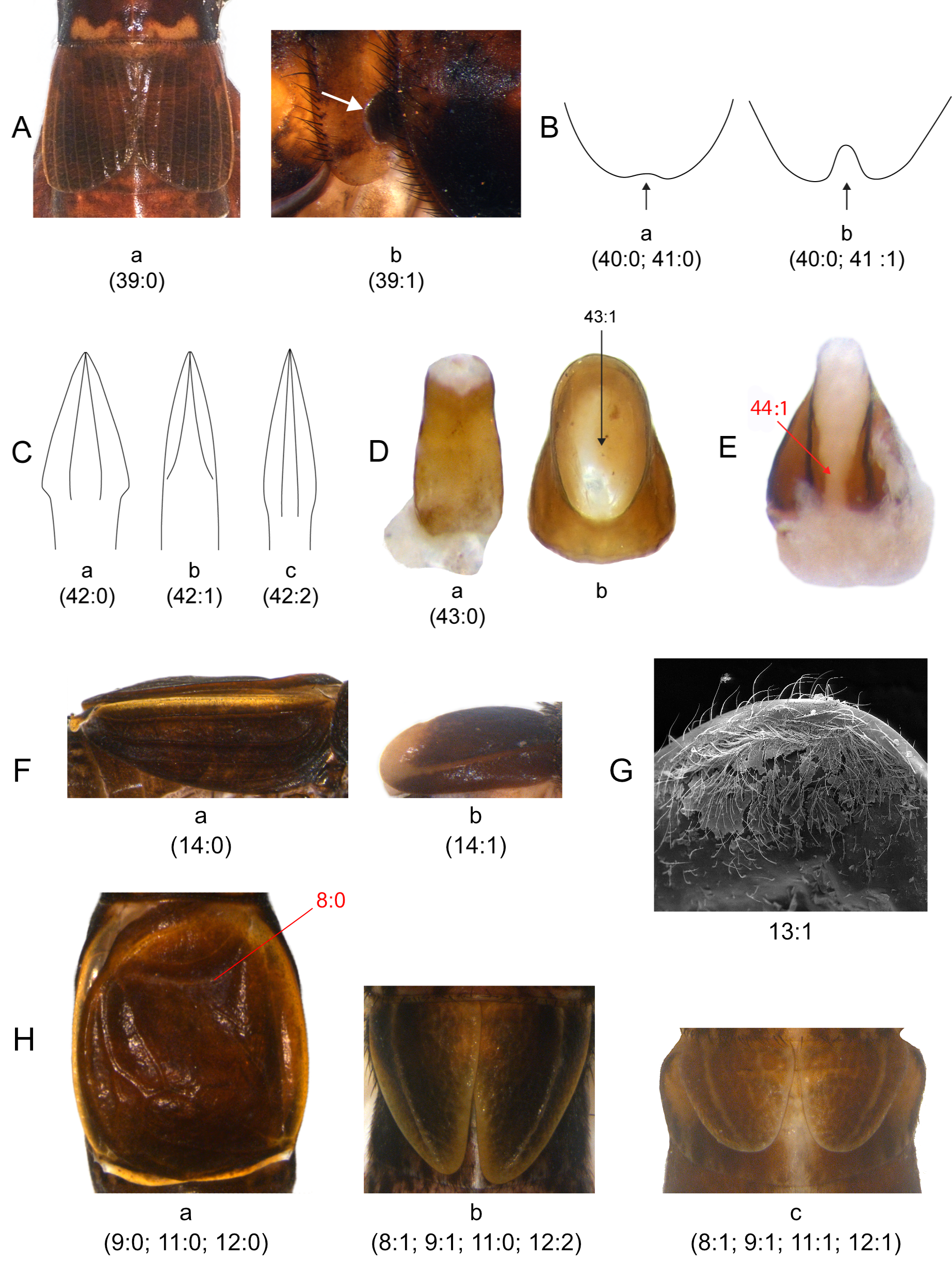

Supplement: S4 Fig — A- Female, tegmina: a-Melanotes ornata, b-Eidmanacris suassunai; B- Female, median invagination of subgenital plate: a-only at the posterior border, b-close or reaching the median part; C-Apex of ovipositor: a- pointed, b- straight, c- curved; D-Copulatory papilla, dorsal: a-Eidmanacris septentrionalis, b-Eidmanacris gigas; E- Strinatia teresopolis, copulatory papilla, dorsal. F- Male, forewing, lateral view: a-Melanotes ornata, b-Eidmanacris dissimilis; G- Eidmanacris meridionalis, apex of forewing, ventral view; H- Forewings, dorsal view: a- Melanotes ornata, b- Eidmanacris desutterae, c- Eidmanacris simoesi. (TIFF) [file pone.0245325.s004.tiff]

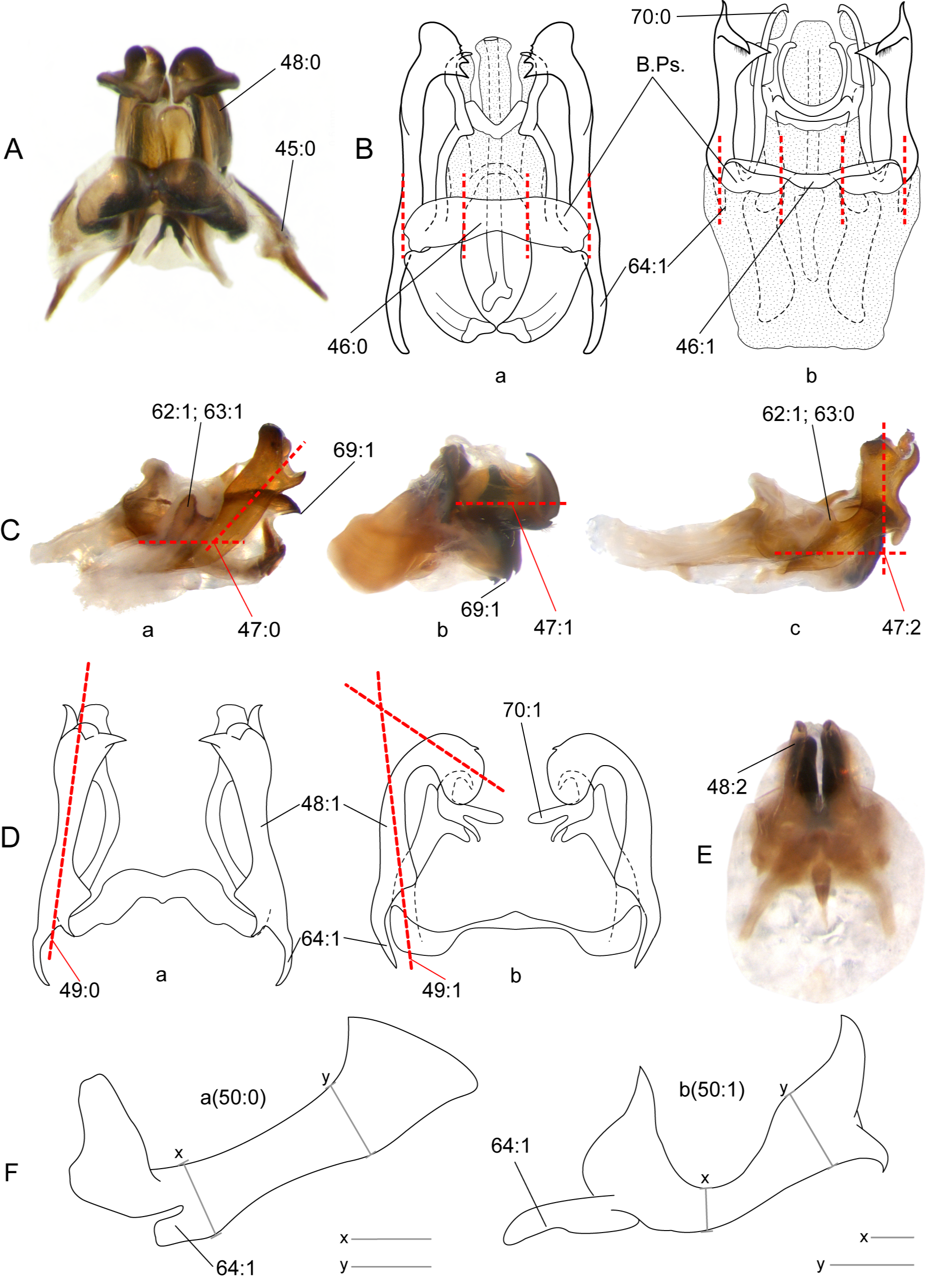

Supplement: S5 Fig — A- Melanotes ornata, phallic complex dorsal; B- Phallic complex, dorsal: a-Eidmanacris multispinosa, b-Eidmanacris tridentata; C- Phallic complex, lateral: a- Eidmanacris bernardii, b-Ottedana cercalis, c-Eidmanacris corumbatai; D- Pseudepiphallic sclerite, dorsal: a-Eidmanacris simoesi, b-Eidmanacris fusca; E-Adenopygus heikoi, dorsal phallic complex; F-Pseudepiphallic sclerite, lateral: a- Eidmanacris larvaeformis, b-Eidmanacris simoesi. (TIFF) [file pone.0245325.s005.tiff]

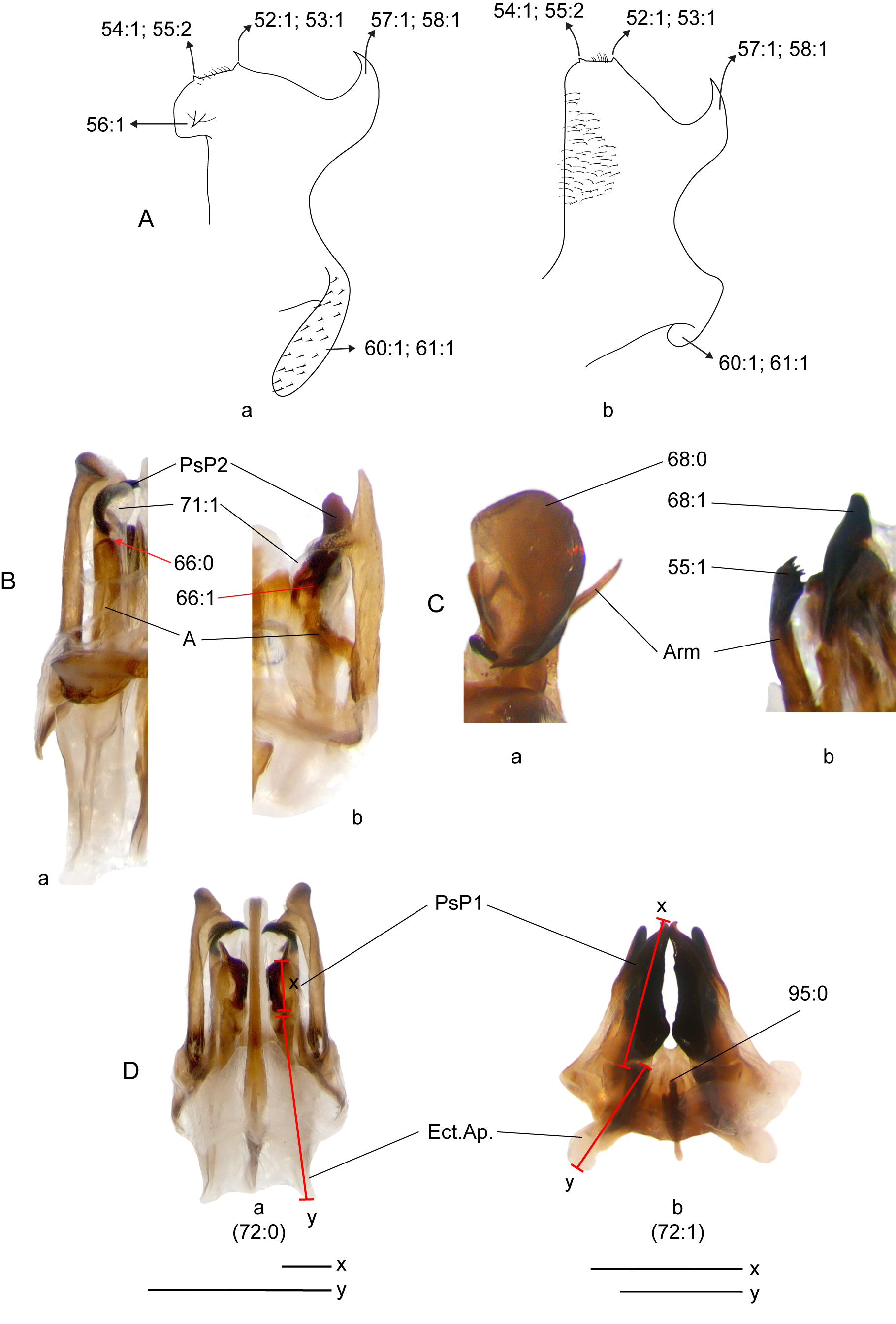

Supplement: S6 Fig — A- Eidmanacris corumbatai, apex of pseudepiphallic arm: a- inner side, b-outer side; B- Phallic complex dorsal: a-Eidmanacris dissimilis, b-Eidmanacris larvaeformis; C- PsP2 and pseudepiphallic arms, dorsal: a-Guabamima saiva, b-Eidmanacris papaveroi; D- Phallic complex, ventral: a-Eidmanacris dissimilis, b- Bambuina bambui. (TIF) [file pone.0245325.s006.tif]

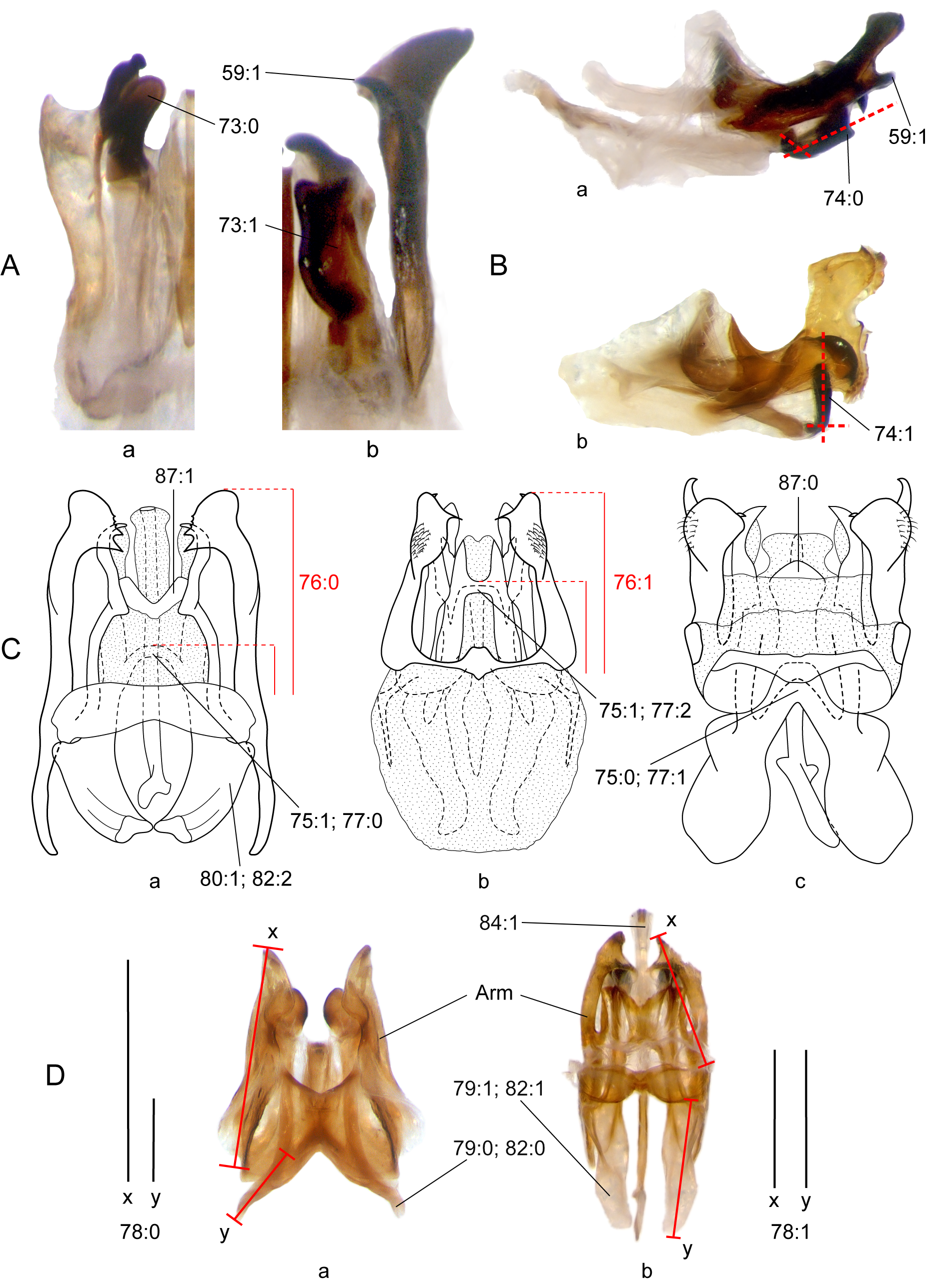

Supplement: S7 Fig — A- Phallic complex ventral: a-Eidmanacris fontanettiae, b-Eidmanacris alboannulata; B- Phallic complex, lateral: a-Eidmanacris alboannulata, b-Eidmanacris desutterae; C- Phallic complex, dorsal: a-Eidmanacris multispinosa, b-Eidmanacris eliethae, c-Eidmanacris bernardii; D- Phallic complex, dorsal: a-Strinatia brevipennis, b-Eidmanacris septentrionalis. (TIFF) [file pone.0245325.s007.tiff]

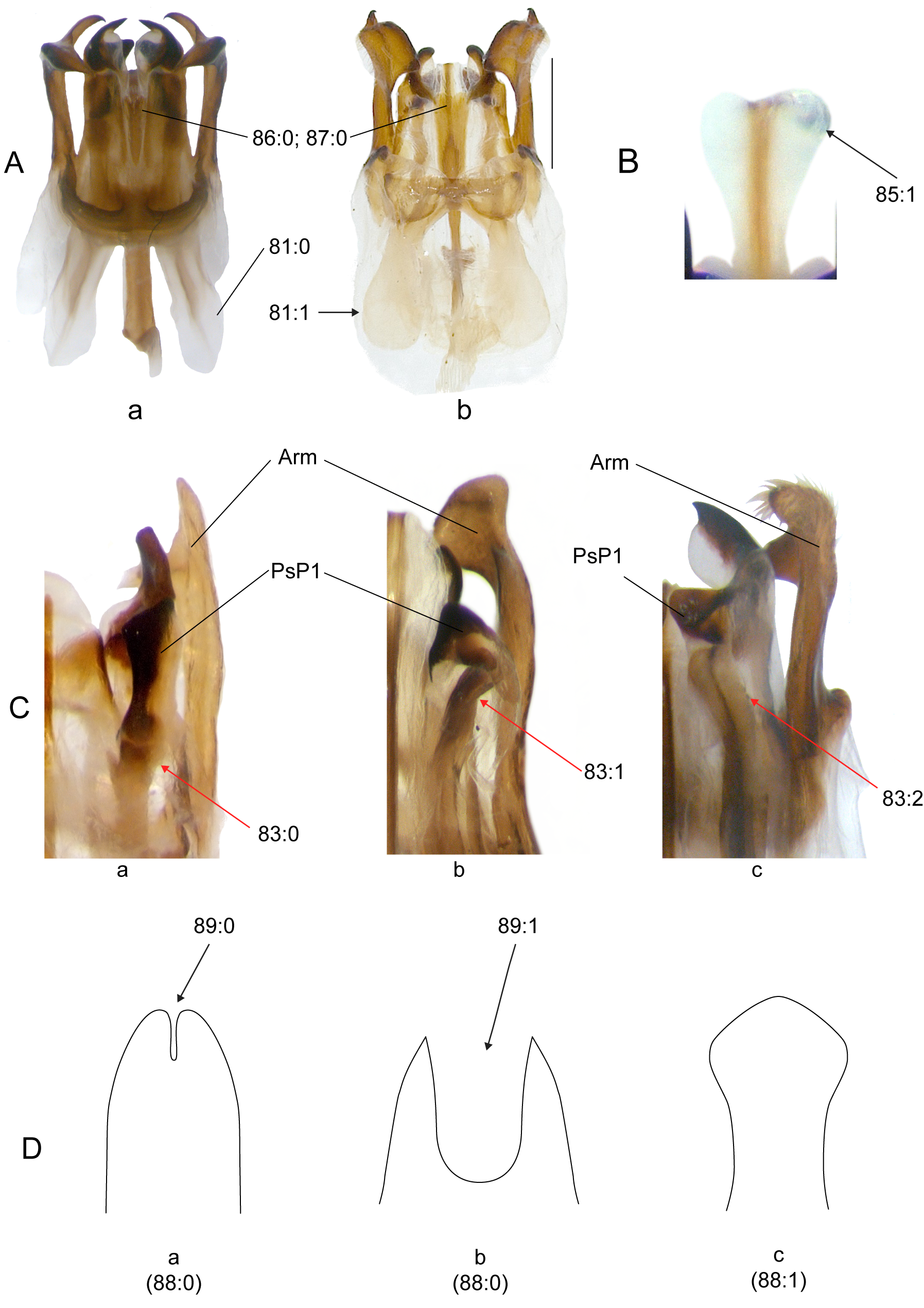

Supplement: S8 Fig — A- Phallic complex, dorsal: a-Eidmanacris suassunai, b-Eidmanacris scopula.; B-Eidmanacris melloi, subapical margins of ectophallic fold; C- Phallic complex, ventral: a-Eidmanacris larvaeformis, b-Eidmanacris multispinosa, c-Eidmanacris caipira; D- Dorsal projection of ectophallic invagination, posterior border: a- only on posterior border, b-reaching median region, c-no concavity. (TIF) [file pone.0245325.s008.tif]

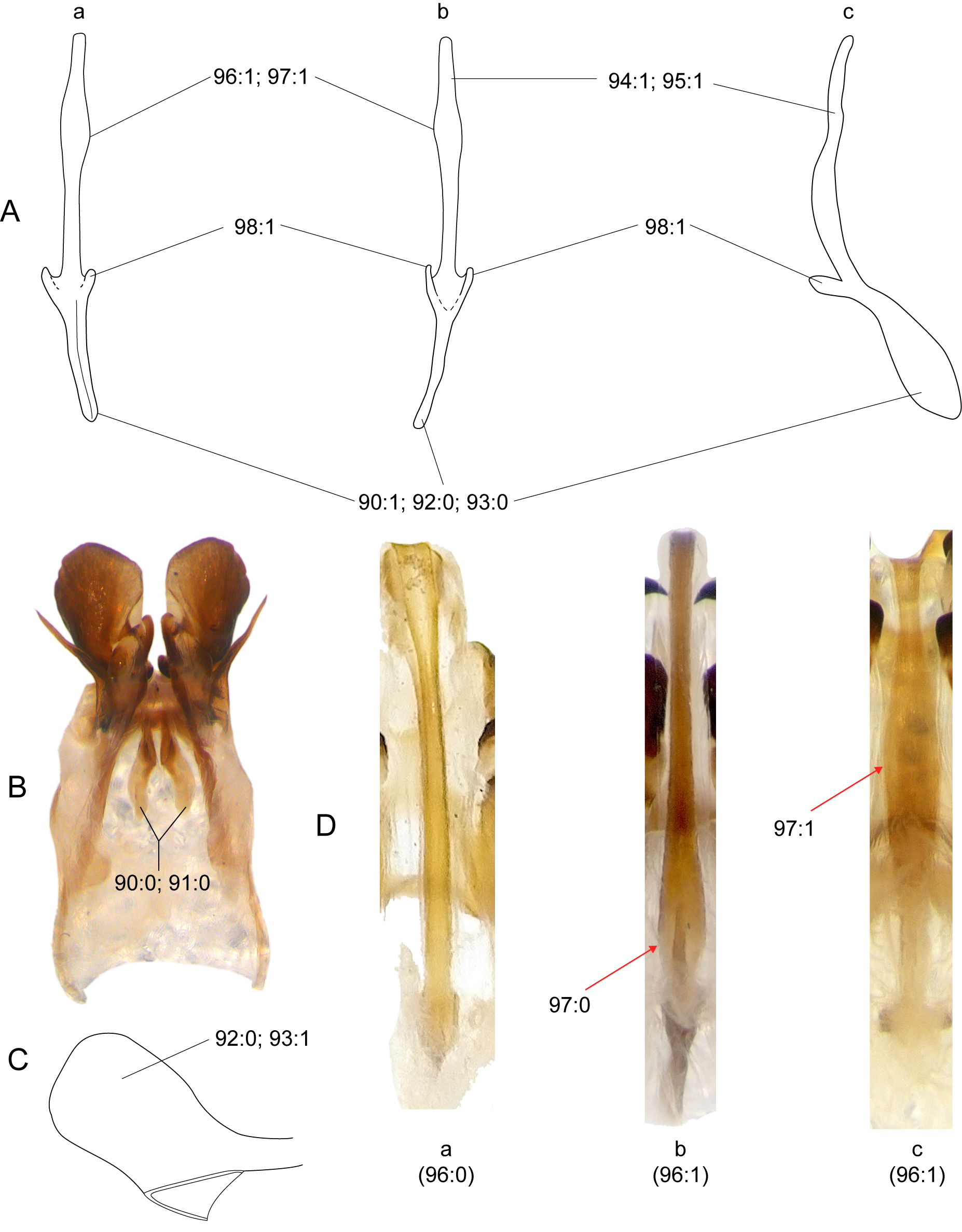

Supplement: S9 Fig — A- Eidmanacris gigas, endophallus: a-dorsal, b-ventral, c-lateral; B- Guabamima lordelloi, phallic complex ventral; C- Eidmanacris minuta, endophallic apodeme lateral; D- Endophallus, ventral: a-Eidmanacris speluncae, b-Eidmanacris alboannulata, c-Eidmanacris meridionalis. (TIF) [file pone.0245325.s009.tif]

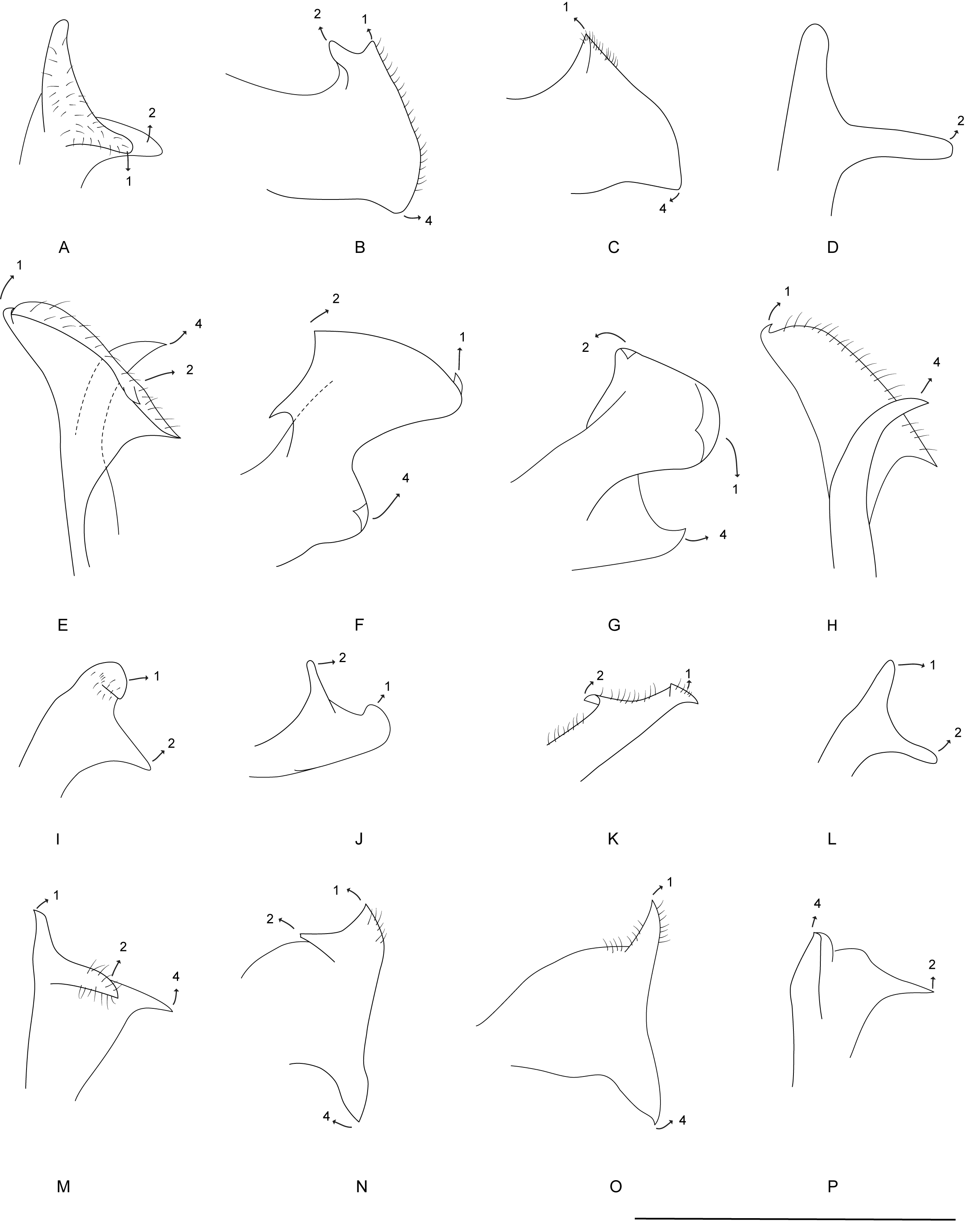

Supplement: S10 Fig — E. larvaeformis: A- dorsal, B- inner side, C- outer side, D- ventral; E. alboannulata: E- dorsal, F- inner side, G- outer side, H- ventral; E. septentrionalis: I- dorsal, J- inner side, K- outer side, L- ventral; E. tridentata: M- dorsal, N- inner side, O- outer side, P- ventral. Projections: 1- superior, 2- supero-internal, 3- infero-internal, 4- inferior. Scale bar: 1 mm. (TIF) [file pone.0245325.s010.tif]

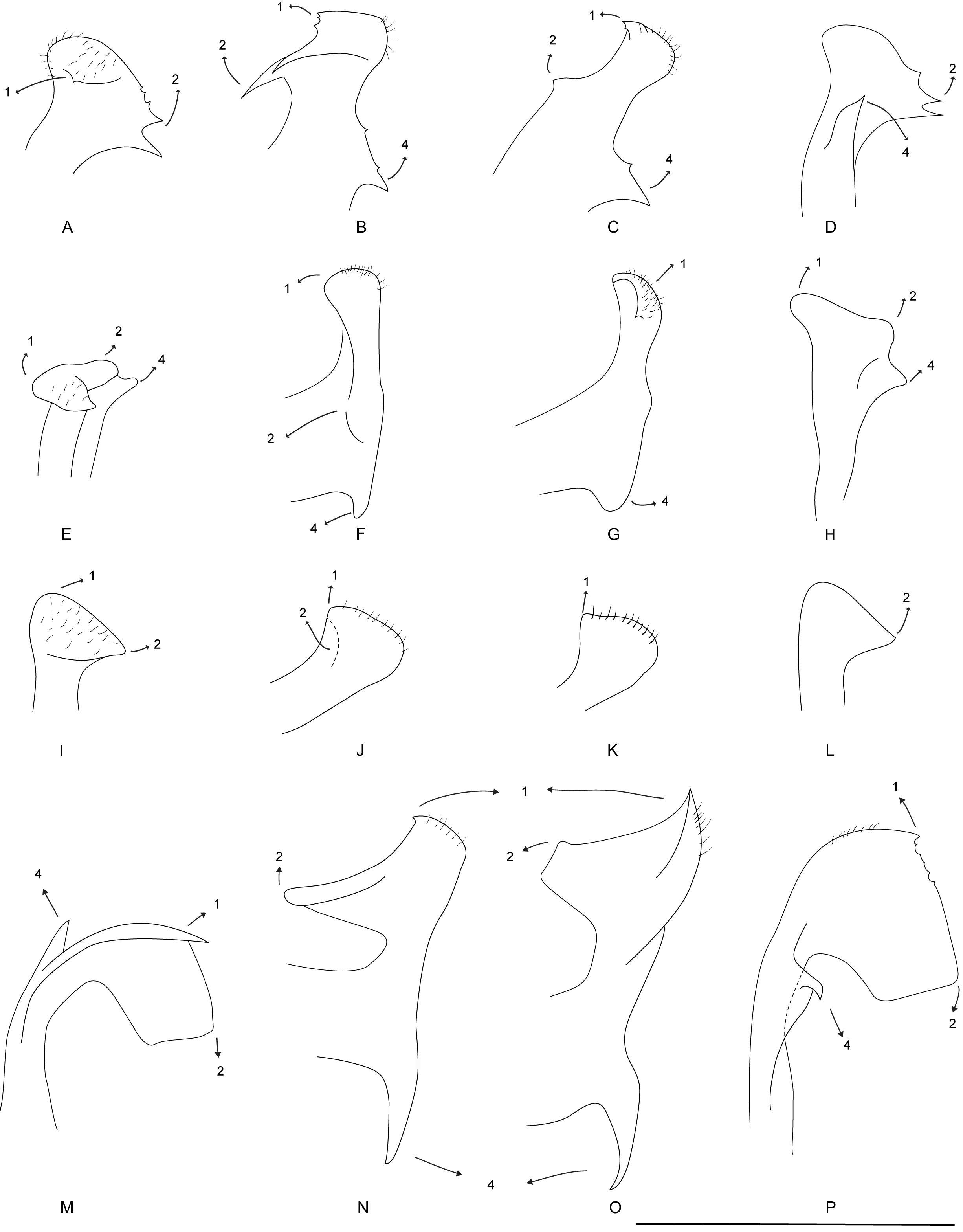

Supplement: S11 Fig — E. multispinosa: A- dorsal, B- inner side, C- outer side, D- ventral; E. dissimilis: E- dorsal, F- inner side, G- outer side, H- ventral; E. meridionalis: I- dorsal, J- inner side, K- outer side, L- ventral; E. fusca: M- dorsal, N- inner side, O- outer side, P- ventral. Projections: 1- superior, 2- supero-internal, 3- infero-internal, 4- inferior. Scale bar: 1 mm. (TIF) [file pone.0245325.s011.tif]

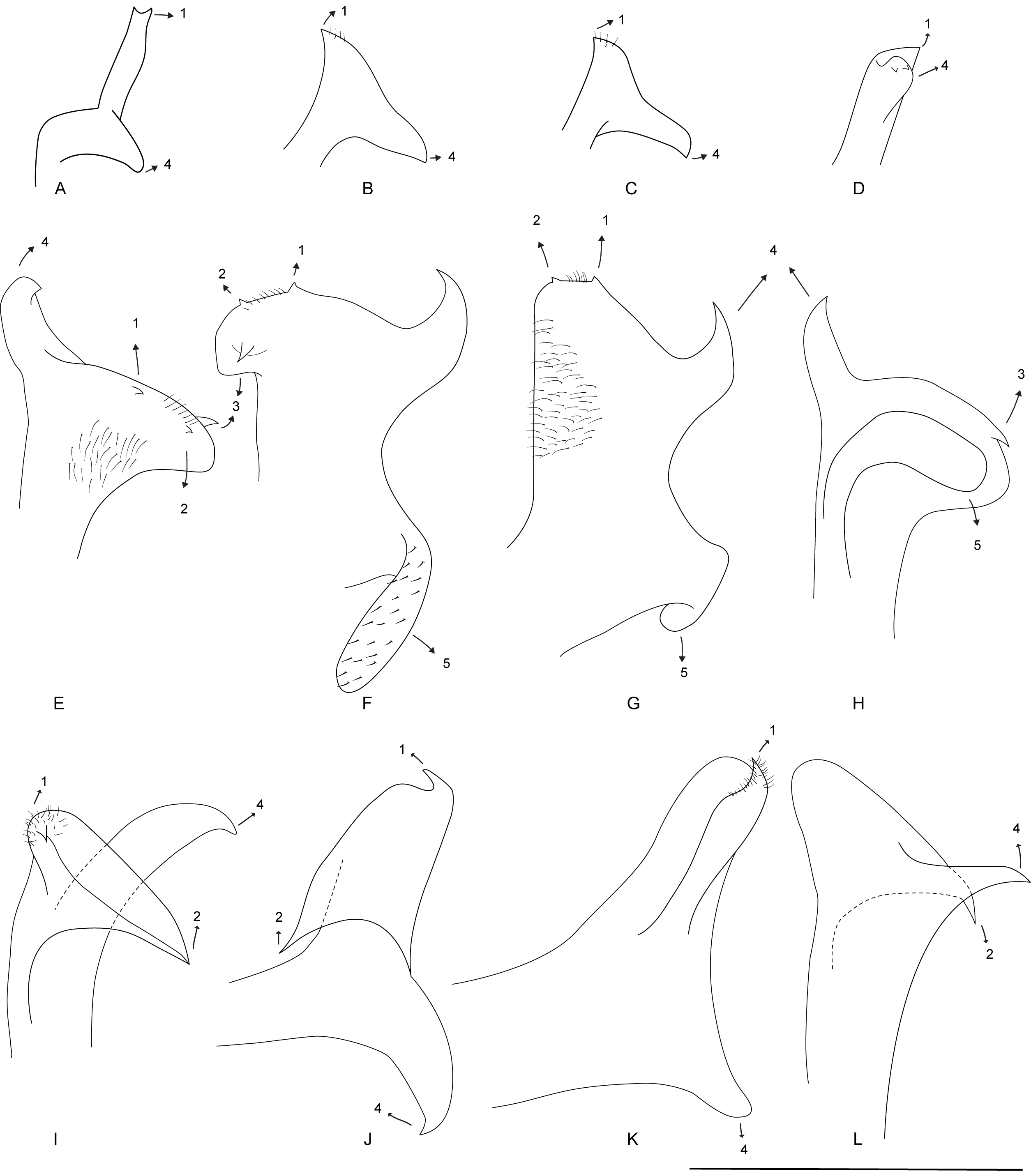

Supplement: S12 Fig — E. bidentata: A- dorsal, B- inner side, C- outer side, D- ventral; E. corumbatai: E- dorsal, F- inner side, G- outer side, H- ventral; E. suassunai: I- dorsal, J- inner side, K- outer side, L- ventral. Projections: 1- superior, 2- supero-internal, 3- infero-internal, 4- inferior, 5- ventral. Scale bar: 1 mm. (TIF) [file pone.0245325.s012.tif]

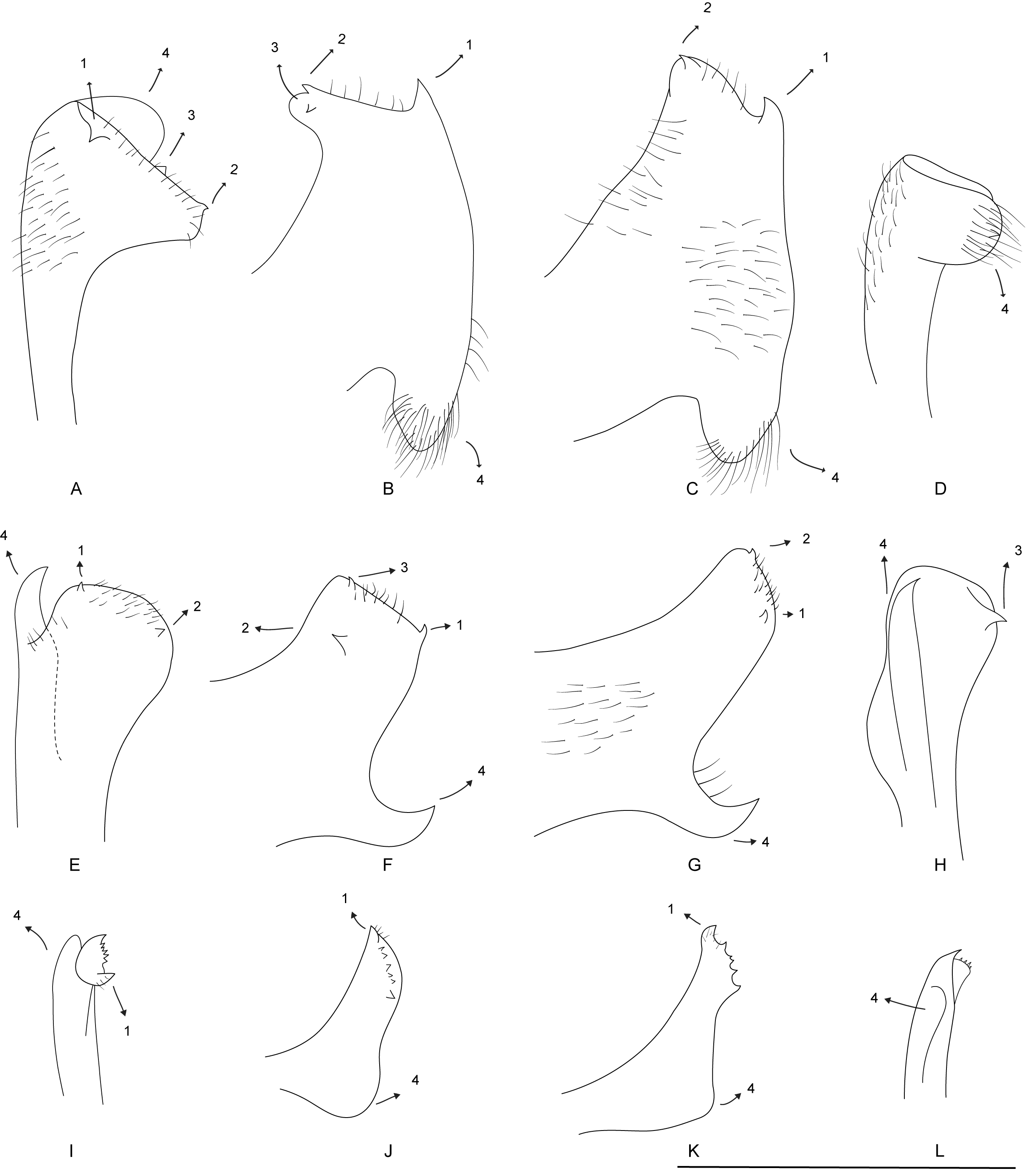

Supplement: S13 Fig — E. caipira: A- dorsal, B- inner side, C- outer side, D- ventral; E. bernardii: E- dorsal, F- inner side, G- outer side, H- ventral; E. papaveroi: I- dorsal, J- inner side, K- outer side, L- ventral. Projections: 1- superior, 2- supero-internal, 3- infero-internal, 4- inferior. Scale bar: 1 mm. (TIF) [file pone.0245325.s013.tif]

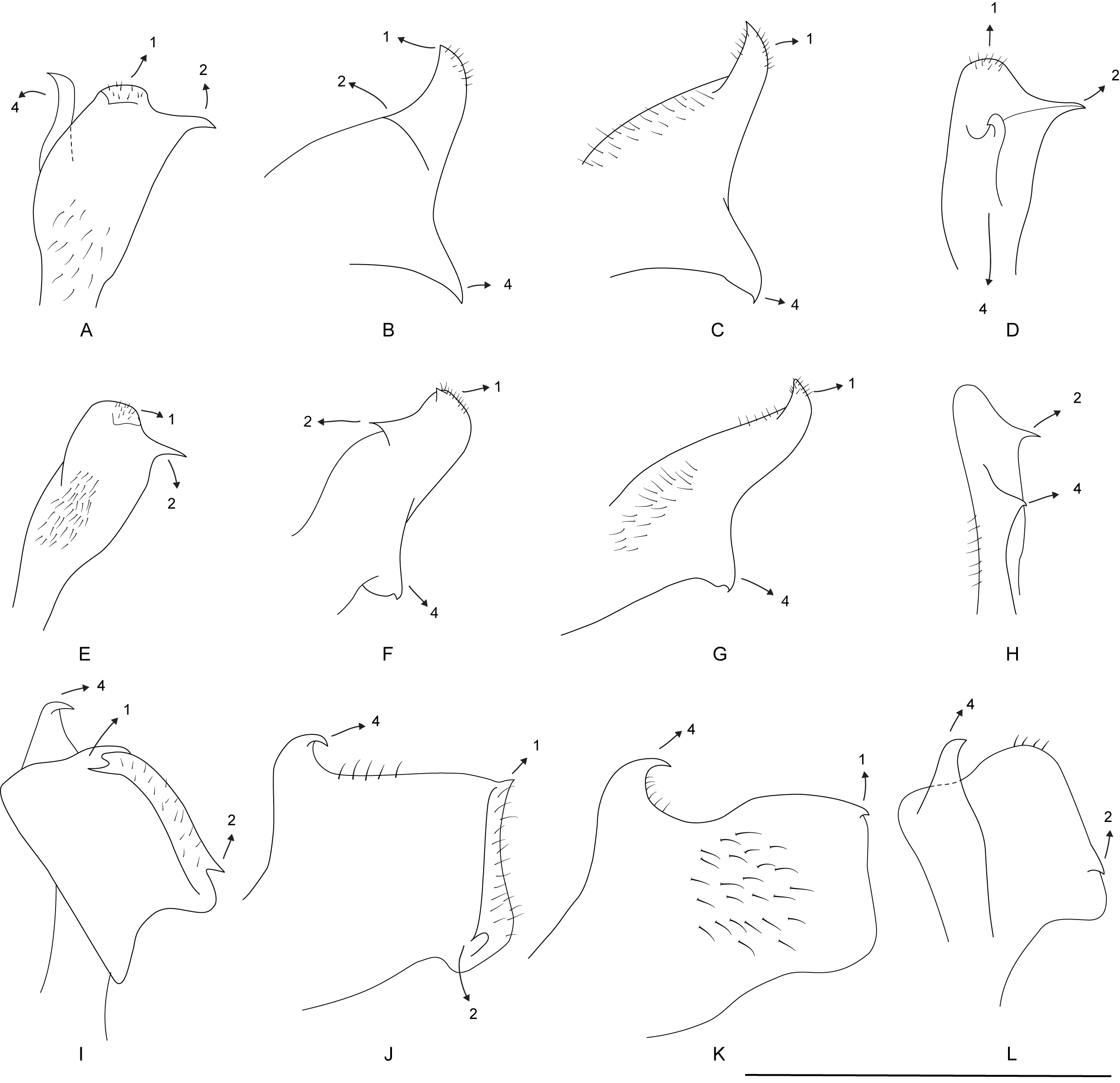

Supplement: S14 Fig — E. simoesi: A- dorsal, B- inner side, C- outer side, D- ventral; E. eliethae: E- dorsal, F- inner side, G- outer side, H- ventral; E. scopula: I- dorsal, J- inner side, K- outer side, L- ventral. Projections: 1- superior, 2- supero-internal, 3- infero-internal, 4- inferior. Scale bar: 1 mm. (TIF) [file pone.0245325.s014.tif]

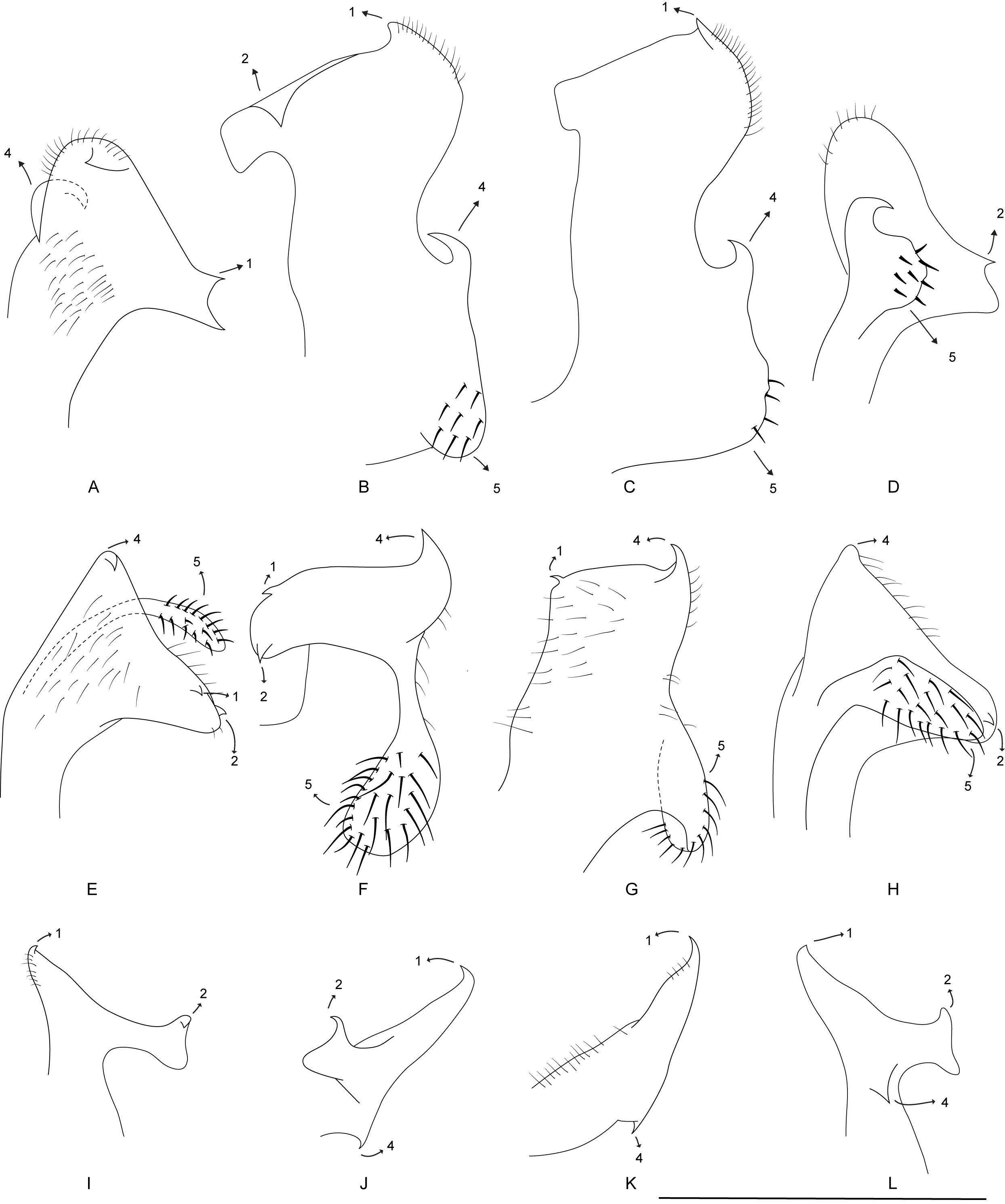

Supplement: S15 Fig — E. desutterae.: A- dorsal, B- inner side, C- outer side, D- ventral; E. gigas: E- dorsal, F- inner side, G- outer side, H- ventral; E. putuhra: I- dorsal, J- inner side, K- outer side, L- ventral. Projections: 1- superior, 2- supero-internal, 3- infero-internal, 4- inferior, 5- ventral. Scale bar: 1 mm. (TIF) [file pone.0245325.s015.tif]

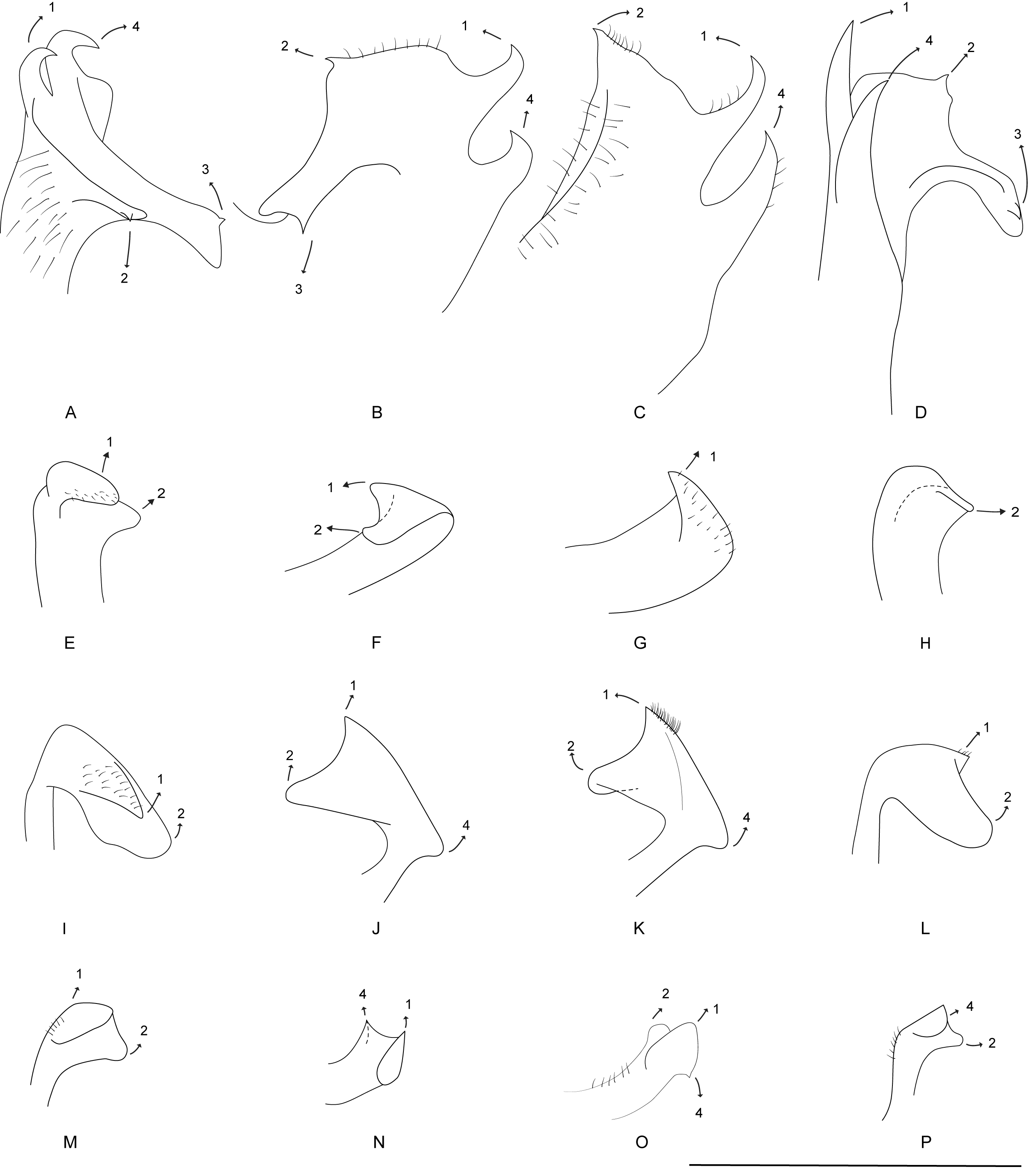

Supplement: S16 Fig — E. neomarmorata: A- dorsal, B- inner side, C- outer side, D- ventral; E. fontanettiae: E- dorsal, F- inner side, G- outer side, H- ventral; E. melloi: I- dorsal, J- inner side, K- outer side, L- ventral; E. speluncae: M- dorsal, N- inner side, O- outer side, P- ventral. Projections: 1- superior, 2- supero-internal, 3- infero-internal, 4- inferior. Scale bar: 1 mm. (TIF) [file pone.0245325.s016.tif]

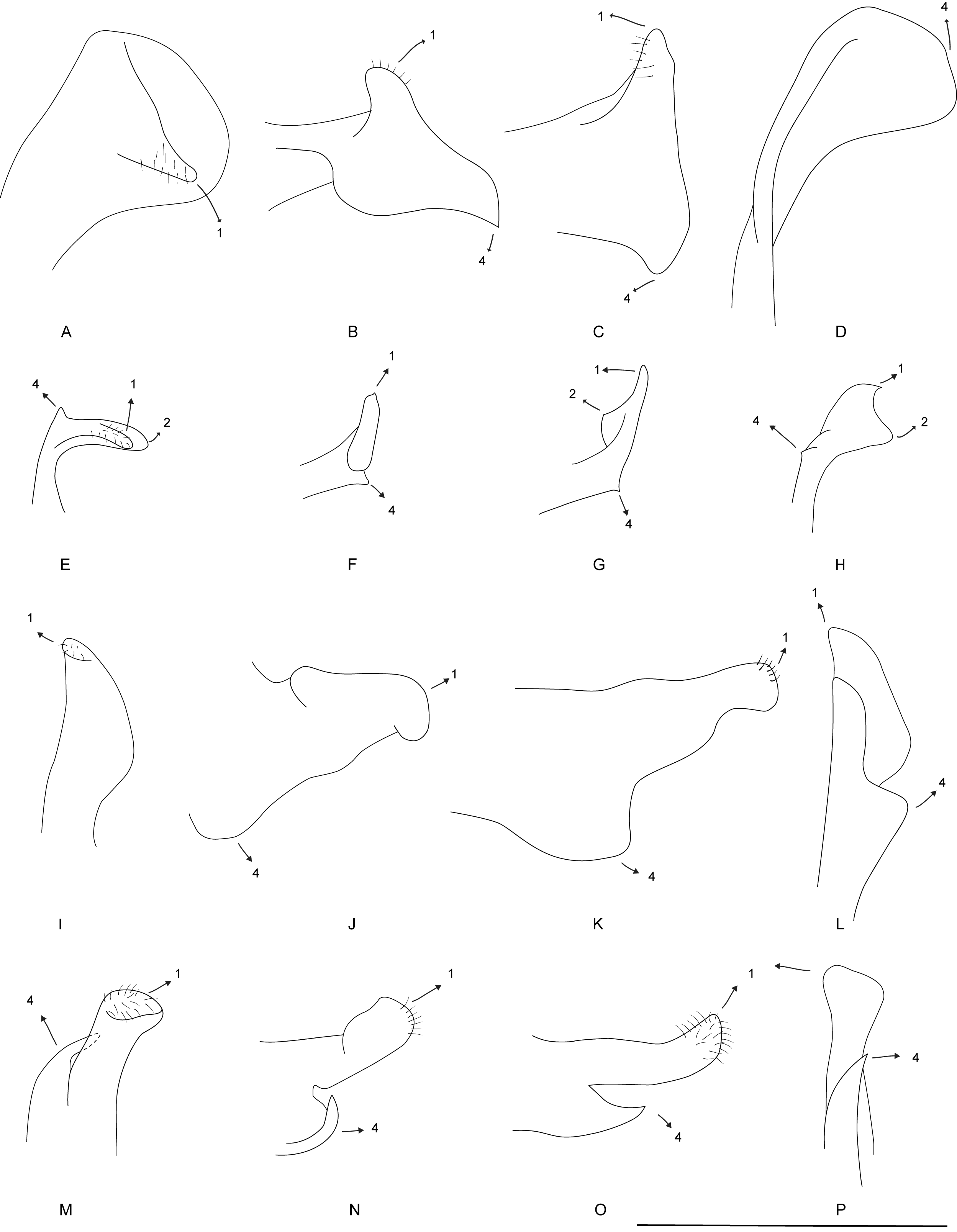

Supplement: S17 Fig — E. endophallica: A- dorsal, B- inner side, C- outer side, D- ventral; E. minuta: E- dorsal, F- inner side, G- outer side, H- ventral; Strinatia brevipennis: I- dorsal, J- inner side, K- outer side, L- ventral; Strinatia teresopolis: M- dorsal, N- inner side, O- outer side, P- ventral. Projections: 1- superior, 2- supero-internal, 3- infero-internal, 4- inferior. Scale bar: 1 mm. (TIF) [file pone.0245325.s017.tif]

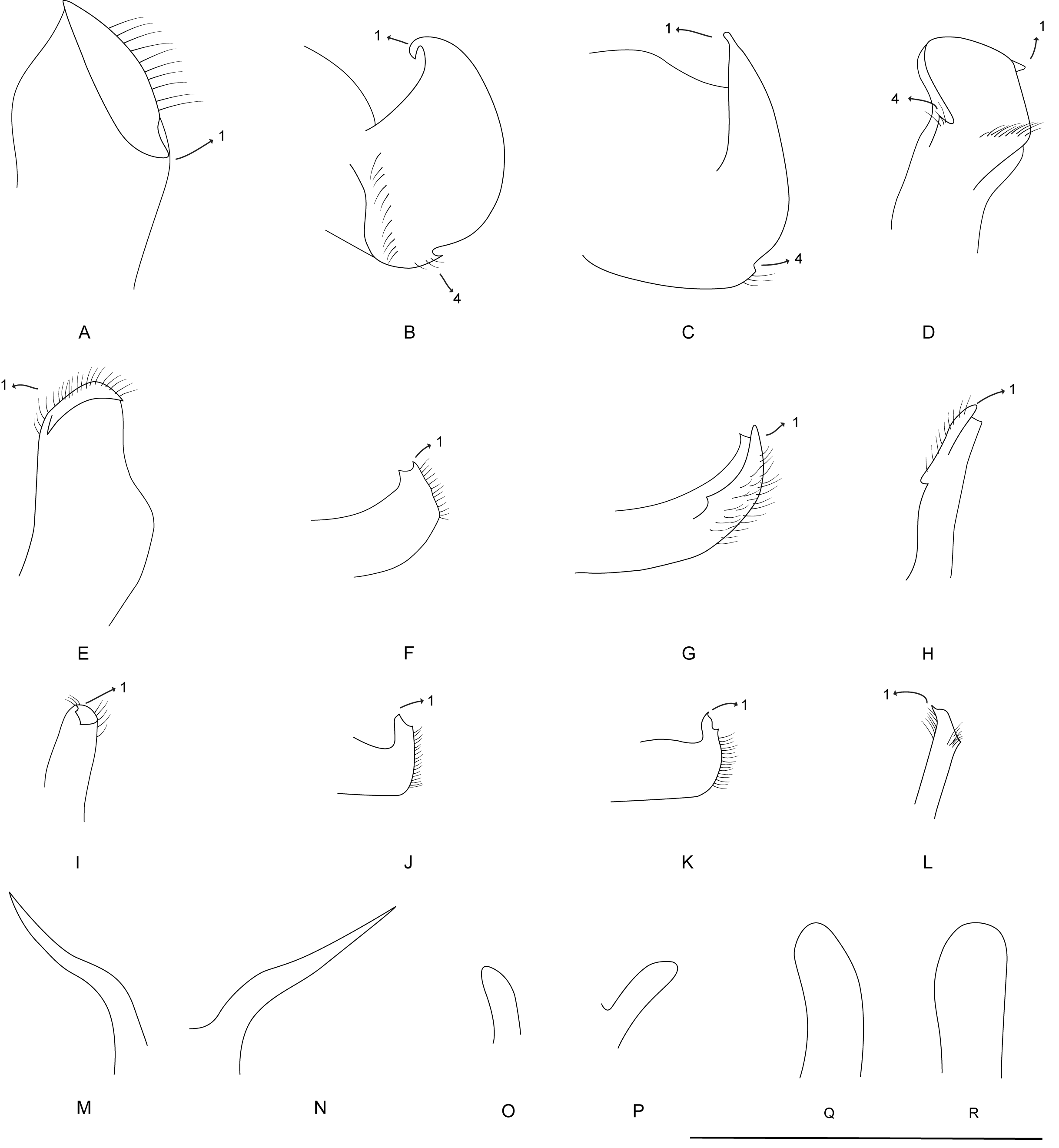

Supplement: S18 Fig — Ottedana cercalis: A- dorsal, B- inner side, C- outer side, D- ventral; Bambuina bambui: E- dorsal, F- inner side, G- outer side, H- ventral; Adenopygus heikoi: I- dorsal, J- inner side, K- outer side, L- ventral; Guabamima lordelloi: M- dorsal, N- ventral; Guabamima saiva: O- dorsal, P- ventral; Melanotes ornata: Q- dorsal, R- Projections: 1- superior, 2- supero-internal, 3- infero-internal, 4- inferior. Scale bar: 1 mm. (TIF) [file pone.0245325.s018.tif]

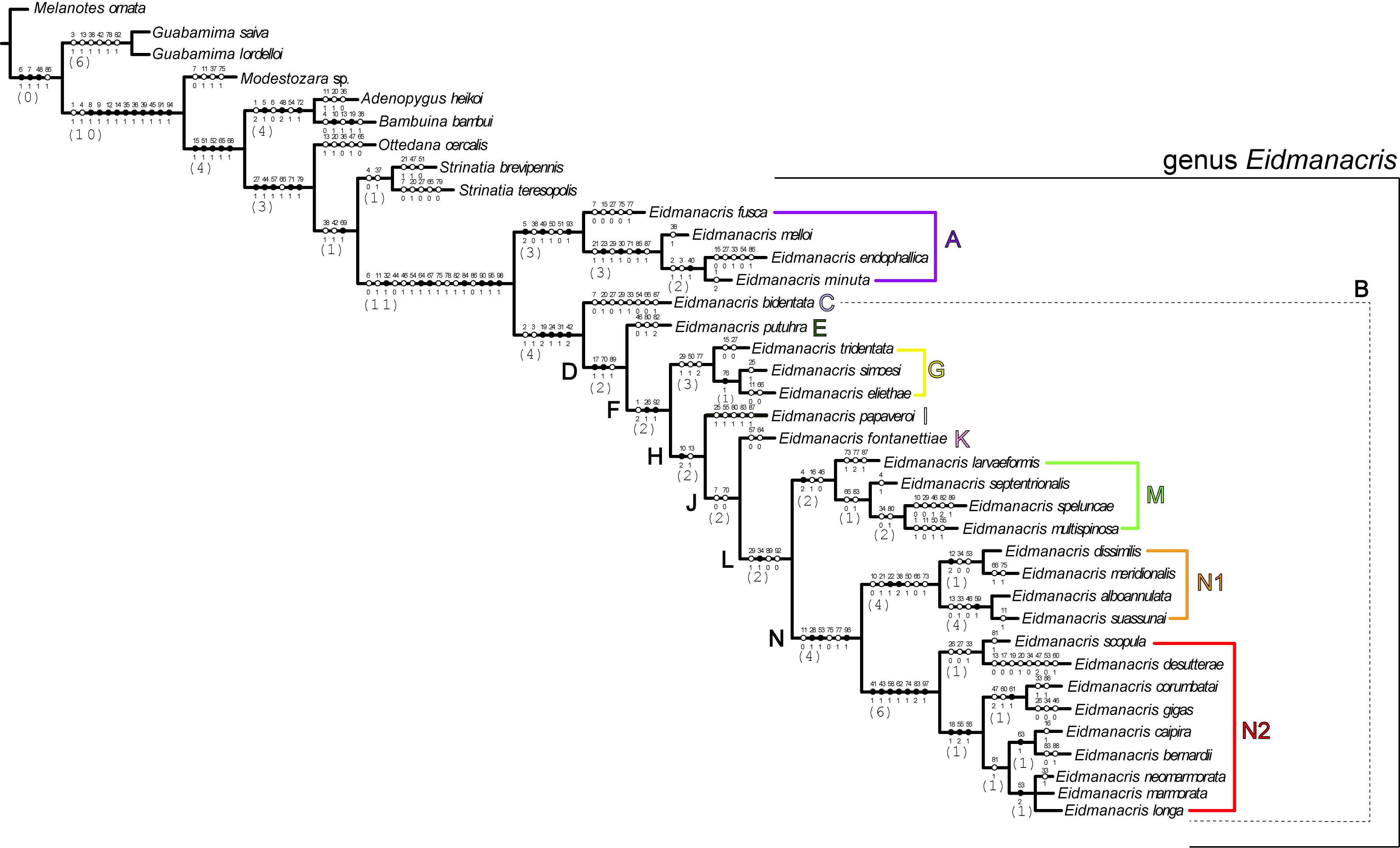

Supplement: S19 Fig — White circles indicate homoplastic synapomorphies, black circles exclusive synapomorphies. Number above circle indicates the characters, above the states. Bremer support are between brackets. (TIF) [file pone.0245325.s019.tif]

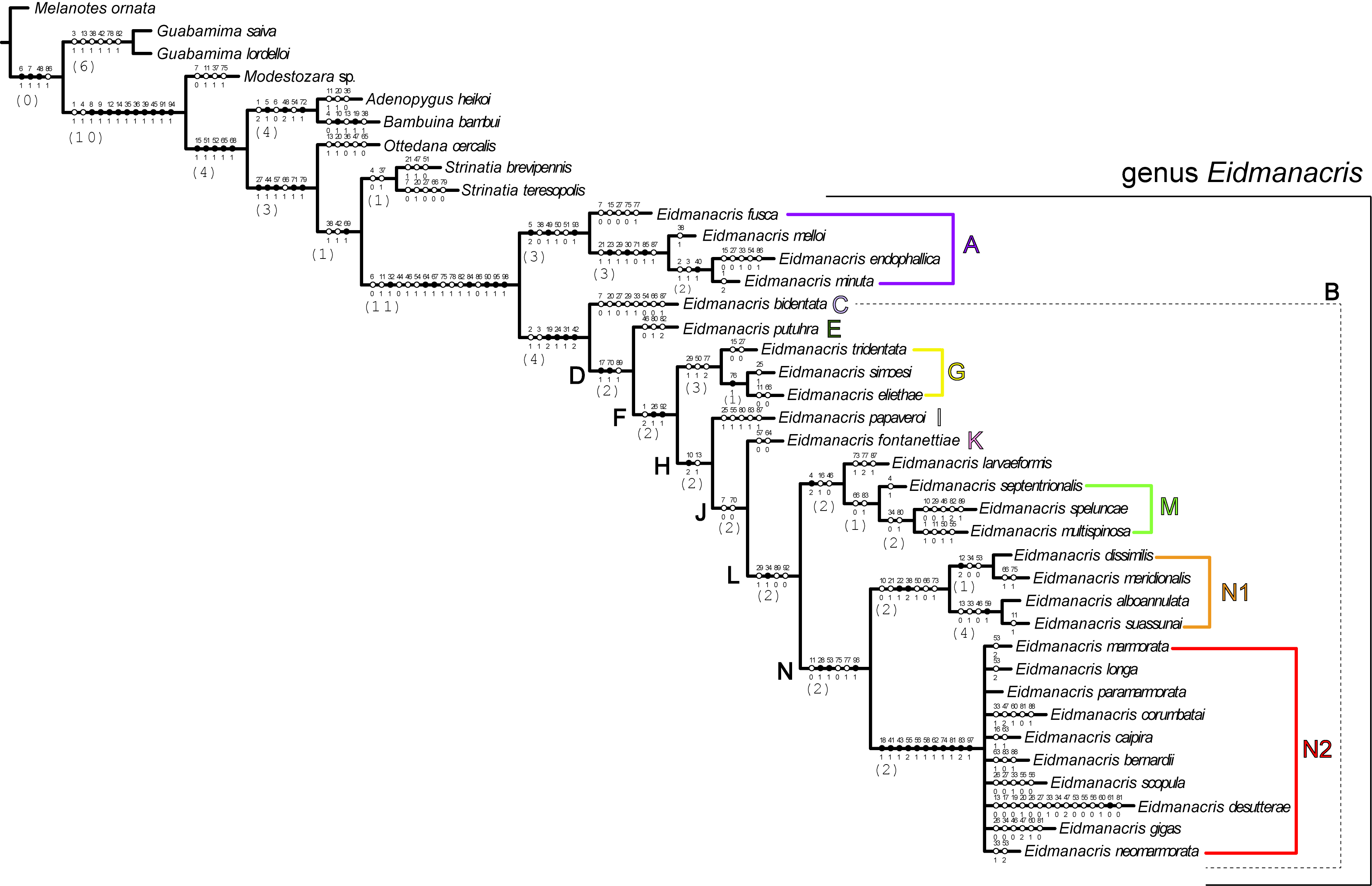

Supplement: S20 Fig — White circles indicate homoplastic synapomorphies, black circles exclusive synapomorphies. Number above circle indicates the characters, above the states. Bremer support are between brackets. (TIF) [file pone.0245325.s020.tif]

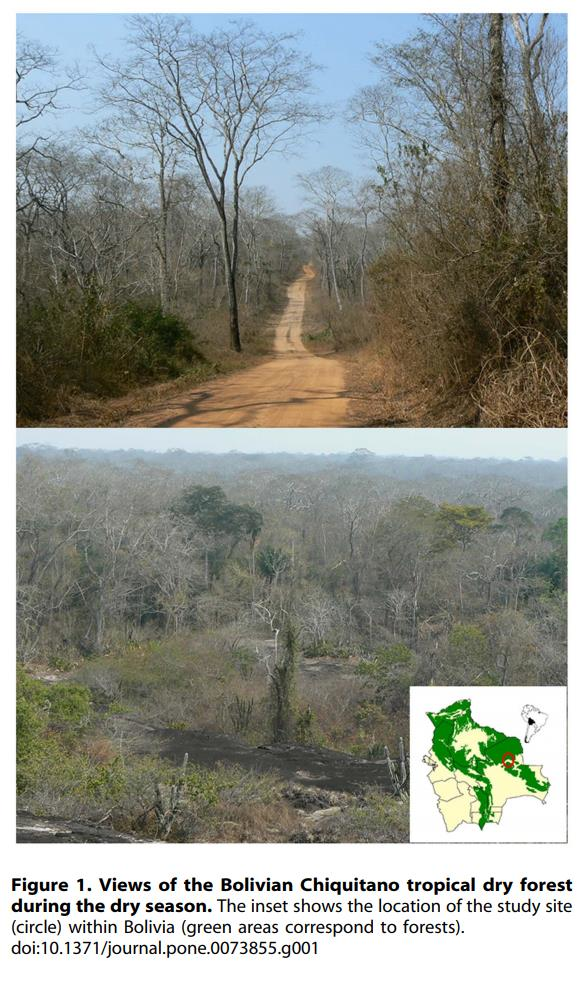

Supplement: S1 Original figure — (TIF) [file pone.0245325.s030.tif]
