# Supplementary material for: Colonization of different biomes drove the diversification of the Neotropical Eidmanacris crickets (Insecta: Orthoptera: Grylloidea: Phalangopsidae)
Source: PLoS One. 2021 Jan 15;16(1):e0245325. doi: 10.1371/journal.pone.0245325 (PMC7810296; doi:10.1371/journal.pone.0245325)
Supplement: S1 File — (DOCX) [file pone.0245325.s021.docx]

**List of characters** (figures S1-S18)

**Head**

1. Head, central ocellus, shape (Fig. S1A): (0) elliptical, (1) spherical, (2) truncated inferiorly; *ci*=40, *ri*=72.

Character proposed by Robillard & Desutter-Grandcolas [1] for the states “spherical” and “elliptical”. Herein, we propose the state “truncated inferiorly” for cases when the inferior border is broader than superior, resulting in a triangular ocellus.

2. Head, frons, median vertical band (Fig. S1B): (0) absent, (1) present; ci=50, ri=90.

Initially considered by Nihei [2] as a three-state character, we consider them different characters (characters 2 and 3).

3. Head, frons, vertical bands below the eyes (Fig. S1B): (0) absent, (1) present; *ci*=33, *ri*=75.

4. Head, antenna (ACCTRAN): (0) Not annulated, (1) annulated, (2) posterior half unpigmented (whitish); *ci*=40, *ri*=57.

5. Head, maxillary palpus, article 5, dorsal curvature (Fig. S1C): (0) slightly curved, (1) not curved, (2) sharply curved; *ci*=100, *ri*=100.

The curvature degree of the fifth article differentiates the states “slightly curved” and “sharply curved” (see Fig. S1C).

6. Head, maxillary palpus, segment 5, area of sensillas, shape (ACCTRAN) (Fig. S1C): (0) rounded, (1) truncated; *ci*=33, *ri*=60.

**Thorax**

7. Thorax, pronotum, dorsal disk, lateral lobes (ACCTRAN): (0) same color as dorsal face (Campos *el al*., 2017; Fig. 6B,C) , (1) different color than dorsal face([3]; Fig. 21B,C); *ci*=16, *ri*=61.

8. Male, thorax, forewings, stridulatory file: (0) present (Fig. S4Ha), (1) absent (Fig. S4Hb, c); *ci*=100, *ri*=100.

9. Male, thorax, forewings, constitution: (0) pergaminous (Fig. S4Ha), (1) coriaceous (Fig. S4Hb, c); *ci*=100; *ri*=100.

The state pergaminous forewings is identified when the veins are evident, like in the outgoup species *Guabamima lordelloi, G. saiva* and *Melanotes ornata*. Coriaceous forewings have a thicker constitution, with veins strongly reduced, usually with only one evident vein separating the dorsal field from a rudimentary lateral field. Moreover, the stridulatory file is absent. The state 1 is possible to observe in *Eidmanacris* species, and in the remain species of the outgroup. Souza-Dias [4] proposed this character.

10. Male, thorax, if forewings are coriaceous (9(1)), shape of apex in dorsal view (Fig. S1D): (0) round, (1) square, (2) triangular; *ci*=50, *ri*=84.

In dorsal view, the state “square” occurs with the ventral convolution of apex of forewing, as in *Bambuina bambui*.

11. Male, thorax, forewings, length related to posterior border of metanotum: (0) reaching or surpassing (Fig S4Ha, b), (1) not reaching (Fig. S4Hc); *ci*=14, *ri*=60.

These character states can be easily observed in dorsal view.

12. Male, thorax, forewings, inner margins in dorsal view: (0) overlapping internally (Fig. S4Ha), (1) not overlapping (Fig. S4Hc), (2) overlapping partially (Fig. S4Hb); *ci*=100, *ri*=100.

The state 0 occurs when the right forewing completely covers left forewing as in *Melanotes ornata*, *Guabamima saiva,* and *Guabamima lordelloi*.

13. Male, thorax, forewings, ventral, apex, bristles: (0) absent, (1) present (Fig. S4G); *ci*=16, *ri*=68.

These bristles are mostly related to the glandular thickening found at the apex of forewings.

14. Male, thorax, forewing, developed lateral field: (0) present (Fig. S4Fa), (1) absent (Fig. S4Fb); *ci*=100, *ri*=100.

The state 0 refers to forewings with a lateral field that covers the metanotum and/or the abdomen. State 1 are regressed lateral fields that do not cover the metanotum in lateral view.

15. Male, thorax, metanotum, glandular area, lateral projection: (0) absent, (1) present (Fig. S1E); *ci*=25, *ri*=50.

Prado & Fontanetti [5] initially proposed this character as “median projections”. However, in the SEM images obtained herein, we identified new projections located between them. We propose using the term “median projections” [5] as “lateral projections”. Therefore, this central projection can be named “median projection” (character 21).

16. Male, thorax, metanotum, glandular area, if lateral projection of metanotum is present (15(1)), surface (DELTRAN) (Fig. S2A): (0) smooth, (1) striated; *ci*=50, *ri*=66.

17. Male, thorax, metanotum, glandular area, if lateral projection of metanotum is present (15(1)), shape (DELTRAN): (0) longer than wide (Fig. S2Da), (1) as long as wide (Fig. S2Ba); *ci*=50, *ri*=87.

18. Male, thorax, metanotum, glandular area, if lateral projection of metanotum is present (15(1)) and they are as long as wide (17(1)), shape (DELTRAN): (0) cylindrical, (1) conical; *ci*=100, *ri*=100.

19. Male, thorax, metanotum, glandular area, if lateral projection of metanotum is present (15(1)), position: (0) directed to pronotum (DELTRAN) (Fig. S2Bb), (1) convergent (Fig. S2Db), (2) parallel to each other (Fig. S2Ba); ci=66, ri=83.

20. Male, thorax, metanotum, glandular area, if lateral projection of metanotum is present (15(1)), internal lateral borders (DELTRAN) (Fig. S2B): (0) not fused, (1) partially or completely fused; *ci*=20, *ri*=0.

21. Male, thorax, metanotum, glandular area, if lateral projection of metanotum is present (15(1)) and its internal lateral borders are not fused (20(0)), median projection (DELTRAN): (0) absent, (1) present (Figs S2C, D); *ci*=33, *ri*=66.

As discussed in character 15, the median projection is between the lateral projections.

22. Male, thorax, metanotum, glandular area if lateral projection of metanotum is present (15(1)), its internal lateral borders are not fused (20(0)), and the median projection is present (21(1)), development of median projection related to lateral projections: (0) same-sized or larger than lateral projections (DELTRAN) (Fig. S2D), (1) very reduced, visible only with SEM (Fig. S2C); *ci*=100, *ri*=100.

23. Male, thorax, metanotum, glandular area, glandular area, if lateral projection of metanotum is present 15(1), its internal lateral borders are not fused (20(0)), the median projection is present (21(1)), and median projection is not reduced (22(0)), shape (DELTRAN) (Fig. S2D): (0) elongated, (1) longitudinal crest.

24. Male, thorax, metanotum, glandular area, anterior border, median crest pronounced: (0) absent, (1) present (Fig. S1E); *ci*=100, *ri*=100.

The fold at the anterior border of metanotum contributes to the formation of this crest.

25. Male, thorax, metanotum, glandular area, anterior border, if median crest pronounced is present (24(1)), bristles: (0) present (Fig. S1Ea), (1) absent (Fig. S1Eb); *ci*=50, *ri*=0.

In many situations, we observed adhered secretions on these bristles. Along with the metanotal bristles (character 27), they apparently retain secretions released in the median crest.

26. Male, thorax, metanotum, glandular area, anterior border, if median crest pronounced is present (24(1)) and it has bristles 25(0), direction: (0) antero-posterior (Fig. S3A), (1) lateral (Fig. S1Ea); *ci*=33, *ri*=50.

This character was proposed by Prado & Fontanetti [5].

27. Male, thorax, metanotum, glandular area, bristles (ACCTRAN): (0) absent, (1) present (Fig. S2D); *ci*=14, *ri=*50.

The absence of these bristles could be associated with little or no use of the secretions liberated in the metanotal region, which do not require the retention of these secretions.

28. Male, thorax, metanotum, glandular area, if bristles are present (27(1)), quantity: (0) occupying the anterior half of metanotum, (1) occupying all the metanotum; *ci*=100, *ri*=100.

As in the previous character, this may be related to the bristles on metanotum and the amount of secretion released there. We supposed that with more bristles, the greater the retention and availability of substances for nuptial gifts for females during copulation. Thus, extending the time of sperm transference through spermatophore, and increasing their reproductive success.

**Abdomen**

29. Male, abdomen, dorsum, sagittal line: (0) absent, (1) present (Fig. S3B); *ci*=20, *ri*=69.

30. Male, abdomen, dorsum, if saggital line is present (29(1)), width: (0) not occupying more than half of dorsal region (DELTRAN), (1) occupying more than half of dorsal region (Fig. S3B); *ci*=100, *ri*=100.

31. Male, abdomen, supra anal plate, median constriction (Fig. S3C): (0) absent; (1) present; *ci*=100, *ri*=100.

32. Male, abdomen, supra anal plate, latero-posterior projections: (0) absent, (1) present (Fig. S3C); *ci*=100, *ri*=100.

33. Male, abdomen, supra anal plate, if latero-posterior projections are present (32(1)), length related to posterior border of the plate (Fig. S3C): (0) shorter, (1) same size or longer; *ci*=16, *ri*=28.

34. Male, abdomen, subgenital plate, posterior border (Fig. S3D): (0) bilobate, (1) straight; *ci*=20, *ri*=50.

**Legs**

35. Tibia I, external auditory tympanum: (0) present, (1) absent; *ci*=100, *ri*=100.

36. Tibia II, dorsal apical spurs, outer (ACCTRAN): (0) present (Fig. S3E), (1) absent; *ci*=33, *ri*=50.

37. Tibia II, dorsal apical spur, inner: (0) present (Fig. S3E), (1) absent; *ci*=50, *ri*=50.

38. Tibia III, inner apical spurs, length (ACCTRAN) (Fig. S3F): (0) dorsal longer than median, (1) median clearly longer than dorsal, (2) median and dorsal almost same-sized; *ci*=33, *ri*=55.

Although the outer apical spurs of tibia III are reduced in relation to the inner apical spurs, they have the same morphological pattern of size mentioned for this character in all the analyzed terminals herein. Therefore, we believe that the development of inner and outer spurs of tibia III are related and we decided not to propose another character for the outer apical spurs of tibia III. This character was proposed by Robillard & Desutter-Grandcolas [1].

**Female exclusive characters**

39. Female, thorax, wings (Fig. S4A): (0) visible, (1) under the posterior border of pronotum, very reduced; *ci*=100, *ri*=100.

The species that display state 1 were initially described without wings in females. However, wings are very reduced in females of *Eidmanacris* species [6], located under the posterior border of the pronotum. Except in *Melanotes ornata*, *Guabamima saiva,* and *Guabamima lordelloi*, the other species of subfamily Luzarinae analyzed in this study have the same condition.

40. Female, abdomen, subgenital plate, posterior border, central invagination: (0) present (Fig. S4B), (1) absent; *ci*=100, *ri*=100.

41. Female, abdomen, subgenital plate, posterior border, if central invagination is present (40(0)), relation to median region of the plate (Fig. S4B): (0) only at the posterior border, (1) close or reaching the median part; *ci*=100, *ri*=100.

42. Female, abdomen, ovipositor, apex in dorsal view (Fig. S4C): (0) pointed, arrow shaped, (1) straight, (2) curved; *ci*=66, *ri*=90.

43. Female, copulatory papilla, posterior aperture (Fig. S4D): (0) inconspicuous, (1) large; *ci*=100, *ri*=100.

44. Female, copulatory papilla, dorsal, median furrow (ACCTRAN): (0) absent, (1) present (Fig. S4E); *ci*=50, *ri*=50.

**Male genitalia**

45. Male, phallic complex, pseudepiphallus, rami: (0) present (Fig. S5A), (1) absent; *ci*=100, *ri*=100.

46. Male, phallic complex, pseudepiphallus, base of pseudepiphallic sclerite, median third related to lateral thirds (Fig. S5B): (0) same width; (1) narrower; *ci*=16, *ri*=66.

The condition 1 refers to the depression in the median part of the base of pseudepiphallic sclerite.

47. Male, phallic complex, pseudepiphallus, pseudepiphallic arm, position related to the base of pseudepiphallic sclerite, in lateral view (Fig. S5C): (0) slightly upcurved; (1) straight; (2) upcurved, forming a 90° angle; *ci*=50, *ri*=30.

48. Male, phallic complex, pseudepiphallus, pseudepiphallic arm, position related to pseudepiphallic parameres, in dorsal view (ACCTRAN): (0) ventral (Fig. S5A), (1) lateral (Fig. S5D), (2) dorsal (Fig. S5E); *ci*=100, *ri*=100.

49. Male, phallic complex, pseudepiphallus, pseudepiphallic arm, apex, curvature in dorsal view (Fig. S5D): (0) not curved inwards, (1) curved inwards; *ci*=100, *ri*=100.

The state 1, curved inwards, refers to the apex with an internal curvature of 90° or less.

50. Male, phallic complex, pseudepiphallic arm, apex, width related to the base of the arm, in lateral view (Fig. S5F): (0) narrow or same width, (1) wider; *ci*=25, *ri*=72.

51. Male, phallic complex, pseudepiphallus, pseudepiphallic arm, apex, bristles: (0) absent, (1) present; *ci*=33, *ri*=75.

52. Male, phallic complex, pseudepiphallic arm, apex, superior projection: (0) absent, (1) present (Fig. S6A); *ci*=100, *ri*=100.

The superior projection of the apex of pseudepiphallic arm is identified because of its characteristic bristles. Based on the location of this projection it is possible to determine the remaining projections (Figs. S10-S18). The superior projection is present in all studied taxa, except in *Melanotes ornata*, *Guabamima lordelloi,* and *Guabamima saiva*.

53. Male, phallic complex, pseudepiphallus, pseudepiphallic arm, apex, if superior projection is present (52(1)), shape (ACCTRAN): (0) not modified (Fig. S10A1), (1) reduced to a spine (Fig. S6A), (2) upcurved hook (Figs. S16A1-D1); *ci*=50, *ri*=75.

54. Male, phallic complex, pseudepiphallus, pseudepiphallic arm, apex, supero-internal projection (ACCTRAN): (0) absent, (1) present (Fig. S6A); *ci*=25, *ri*=62.

This is the projection of the apex of pseudepiphallic arm internal to the superior projection (character 52). We named this “supero-internal” because some taxa have two internal projections: supero-internal and infero-internal (character 56).

55. Male, phallic complex, pseudepiphallus, pseudepiphallic arm, apex, if supero-internal projection is present (54(1)), form: (0) not modified, (1) serrulated (Figs. S6Cb; S11A2, D2), (2) reduced to a spine (Fig. S6A); *ci*=66, *ri*=85.

56. Male, phallic complex, pseudepiphallus, pseudepiphallic arm, apex, infero-internal projection: (0) absent, (1) present (Fig. S6A); *ci*=100, *ri*=100.

This projection is located inferiorly to the supero-internal projection (character 54).

57. Male, phallic complex, pseudepiphallus, pseudepiphallic arm, apex, inferior projection: (0) absent, (1) present (Fig. S6A); *ci*=50, *ri*=83.

As the name suggests, this projection is inferior to the superior projection. It may be observed in lateral view.

58. Male, phallic complex, pseudepiphallus, pseudepiphallic arm, apex, if inferior projection is present (57(1)), curvature: (0) not curved, (1) upcurved hook (Fig. S6A); *ci*=100, *ri*=100.

59. Male, phallic complex, pseudepiphallus, pseudepiphallic arm, apex, If inferior projection is present (57(1)), position in ventral view: (0) straight, (1) curved inwards (Fig. S7A, B; S10H4); *ci*=100, *ri*=100.

60. Male, phallic complex, pseudepiphallus, pseudepiphallic arm, apex, ventral projection: (0) absent, (1) present (Fig. S6A); *ci*=50, *ri*=50.

This projection appears in three species of *Eidmanacris*: *E. corumbatai*, *E. gigas,* and *E. desutterae*. It is a characteristic structure anterior to the inferior projection (character 57) and curved internally. It may be better observed in the ventral view.

61. Male, phallic complex, pseudepiphallus, pseudepiphallic arms, apex, if ventral projection is present (60(1)), length in lateral view (DELTRAN): (0) almost the same width of pseudepiphallic arm (Fig. S15D5), (1) reaches median region of the phallic complex (Fig. S12H5).

Only *E. corumbatai*, *E. gigas,* and *E. desutterae* have this ventral projection, as commented in the previous character (character 60).

62. Male, phallic complex, pseudepiphallus, lateral projection of pseudepiphallic sclerite: (0) absent, (1) present (Fig. S5C); *ci*=100, *ri*=100.

This is a very characteristic structure, located in the delimitation between the base of pseudepiphallic sclerite and pseudepiphallic arm, and may be observed in lateral view. This character is one of the synapomorphies that supports clade N2.

63. Male, phallic complex, pseudepiphallus, If lateral projection of pseudepiphallic sclerite is present (62(1)), apex (Fig. S5C): (0) pointed, (1) rounded; *ci*=100, *ri*=100.

64. Male, phallic complex, pseudepiphallus, anterior projection of pseudepiphallic sclerite: (0) absent, (1) present (Figs. S5B, E); *ci*=50, *ri*=88.

Referred to as the “ventral projection of pseudepiphallic sclerite” by Souza-Dias [4], we considered this structure as “anterior”. In some situations, it is the first part of the pseudepiphallic sclerite. We did not consider this a ventral structure because in some species it is centralized in lateral view, as in *E. larvaeformis*. Even though they occupy a similar place as rami, these structures are not homologues. The anterior projections are integral structures of the pseudepiphallic sclerite. This character is one of the main synapomorphies of *Eidmanacris* that contributes to genus’ monophyly.

65. Male, phallic complex, pseudepiphallus, sclerite A, size related to pseudepiphallic arm in dorsal view (ACCTRAN): (0) shorter than half, (1) longer than half; *ci*=50, *ri*=75.

66. Male, phallic complex, pseudepiphallus, sclerite A, connection with dorsal lobe of pseudepiphallic paramere (PsP2) (ACCTRAN) (Fig. S6B): (0) not continuous, (1) continuous; *ci*=14, *ri*=57.

67. Male, phallic complex, pseudepiphallus, pseudepiphallic parameres, dorsal and ventral lobes (PsP2 and PsP1, respectively), connection: (0) sclerotized, (1) not sclerotized; *ci*=100, *ri*=100.

The pseudepiphallic parameres are highly variable structures, and the main clasping devices present in members of Grylloidea. In Phalangopsidae, mainly in Luzarinae, each pseudepiphallic paramere is divided in two lobes (or projections), one pair is usually ventrally visible (PsP1), while the other is apical and dorsally visible (PsP2).

In this study, only *Eidmanacris* species have a membranous connection between these two lobes. For more information about the pseudepiphallic parameres in Grylloidea and in Phalangopsidae please see Souza-Dias *et al.* [6] and references therein.

68. Male, phallic complex, pseudepiphallus, pseudepiphallic parameres, dorsal lobe (PsP2), shape (Fig. S6C): (0) rounded, (1) elongate; *ci*=100, *ri*=100.

The state “rounded” refers to the apical region of the pseudepiphallic paramere. This occurs in *Guabamima lordelloi*, *Guabamima saiva*, *Modestozara* sp. and *Melanotes ornata*.

69. Male, phallic complex, pseudepiphallus, pseudepiphallic parameres, dorsal lobe (PsP2), apex in lateral view: (0) not pointed, (1) pointed (Fig. S5C); *ci*=100, *ri*=100.

70. Male, phallic complex, pseudepiphallus, pseudepiphallic parameres, if apex of dorsal lobe (PsP2) is pointed in lateral view: (0) single (Fig. S5Bb), (1) bifid (Fig. S5Db); *ci*=50, *ri*=75.

71. Male, phallic complex, pseudepiphallus, pseudepiphallic parameres, dorsal lobe (PsP2), inner face, membranous spheres: (0) absent, (1) present (Fig. S6B); *ci*=50, *ri*=87.

These spheres are recognizable because are punctuated. They are located on the inner face of PsP2. According to De Mello & De Andrade [7], these spheres may act as claspers, holding the female copulatory papilla by hydraulic pressure.

72. Male, phallic complex, pseudepiphallus, pseudepiphallic parameres, ventral lobe (PsP1), length related to ectophallic apodeme (Fig. S6D): (0) shorter, (1) same size or longer; *ci*=100, *ri*=100.

73. Male, phallic complex, pseudepiphallus, pseudepiphallic parameres, ventral lobe (PsP1), accentuated sclerotization (Fig. S7A): (0) complete or almost complete, (1) only on the inner margin; *ci*=50, *ri*=75.

74. Male, phallic complex, pseudepiphallus, pseudepiphallic parameres, ventral lobe (PsP1), up curvature in lateral view (Fig. S7B): (0) forming an angle greater than 90°, (1) forming a 90° angle: *ci*=100, *ri*=100.

75. Male, phallic complex, ectophallic invagination, ectophallic arc, position in dorsal view (ACCTRAN) (Fig. S7C): (0) anterior or below the base of pseudepiphallic sclerite, (1) posterior to the base of pseudepiphallic sclerite; *ci*=20, *ri*=73.

This character was proposed and used in other phylogenetic studies of Grylloidea [1,2,4,8].

76. Male, phallic complex, ectophallic invagination, ectophallic arc, if the arc is posterior to the base pseudepiphallic sclerite in dorsal view (75(1)), (Fig. S7C): (0) not surpassing the anterior half of pseudepiphallic arm, (1) surpassing the anterior half of pseudepiphallic arm; *ci*=100, *ri*=100.

77. Male, phallic complex, ectophallic invagination, ectophallic arc, shape in dorsal view (Fig. S7C): (0) convex, (1) “V” shaped, (2) straight; ci=50, ri=87.

78. Male, phallic complex, ectophallic invagination, ectophallic apodeme, length related to pseudepiphallic arm in dorsal view (Fig. S7D): (0) shorter, (1) same size or longer; *ci*=50, *ri*=83.

79. Male, phallic complex, ectophallic invagination, ectophallic apodeme, shape: (0) not flattened (Fig. S7Da), (1) flattened (Fig. S7Db); *ci*=50, *ri*=83.

80. Male, phallic complex, ectophallic invagination, if ectophallic apodeme is flattened (79(1)), orientation: (0) not curved, (1) curved (Fig. S7Ca); *ci*=33, *ri*=33.

The state “curved” refers to the torsion of the apodeme. It is more visible in lateral view.

81.Male, phallic complex, ectophallic invagination, if ectophallic apodeme is flattened (79(1)), and it is not curved (80(0)), proximal region in dorsal view: (0) not enlarged, (1) enlarged (Fig. S8A); *ci*=50, *ri*=80.

82. Male, phallic complex, ectophallic invagination, ectophallic apodeme, apex orientation: (0) divergent (Fig. S7Da), (1) straight (Fig S7Db), (2) convergent (Fig. S7Ca); *ci*=50, *ri*=71.

83. Male, phallic complex, ectophallic invagination, ventral posterior projection, orientation (Fig. S8C): (0) straight, (1) curved externally, (2) curved internally; *ci*=50, *ri*=77.

By determining this structure, it is possible to locate the pseudepiphallic paramere, since they are closely related.

84. Male, phallic complex, ectophallic fold: (0) not entirely membranous, (1) membranous (Fig. S7Db); *ci*=100, *ri*=100.

85. Male, phallic complex, if ectophallic fold is membranous (84(1)), subapical margins laterally inflated: (0) absent, (1) present (Fig. S8B); *ci*= 100, *ri*=100.

86. Male, phallic complex, ectophallic invagination, sclerotized dorsal projection: (0) present (Fig. S8A), (1) absent; *ci*=33, *ri*=75.

This structure is posterior to the ectophallic arc. It is also found in *Modestozara* sp., but coded as absent for that species because here since it is membranous (autapomorphy for *Modestozara* sp.). This structure is found in other Luzarinae genera (*e.g.* *Endecous*).

87. Male, phallic complex, ectophallic invagination, if sclerotized dorsal projection is present (86(0)), size: (0) longer than wide, or as long as wide (Fig. S7Cc; S8Ab), (1) wider than long (Fig. S7Ca); *ci*=25, *ri*=25.

88. Male, phallic complex, ectophallic invagination, if sclerotized dorsal projection is present (86(0)), posterior border concavity (Fig. S8D): (0) present, (1) absent; *ci*=50, *ri*=0.

89. Male, phallic complex, ectophallic invagination, if sclerotized dorsal projection is present (86(0)),and it has a posterior border concavity (88(0)), concavity size (Fig. S8D): (0) only on posterior border, (1) reaching the median region of the structure; *ci*=33, *ri*=60.

90. Male, phallic complex, endophallus, endophallic apodeme, form: (0) lamellar (Fig. S9B), (1) crest (Fig. S9A); *ci*=100, *ri*=100.

Even though state 1 appears in our analysis as a synapomorphy for *Eidmanacris*, it is not exclusive to this genus. This character also appears in Eneopterinae taxa [1].

91. Male, phallic complex, endophallus, if endophallic apodeme is lamellar (90(0)), number: (0) paired (Fig. S9B), (1) single; *ci*=100, *ri*=100.

92. Male, phallic complex, endophallus, if endophallic apodeme is a crest (90(1)), shape: (0) flattened (Fig. S9A; C), (1) not flattened; *ci*=50, *ri*=75.

93. Male, phallic complex, endophallus, if endophallic apodeme is a crest (90(1)), size (ACCTRAN): (0) longer than wide (Fig. S9A), (1) as wide as long (Fig. S9C); *ci*=100, *ri*=100.

94. Male, phallic complex, endophallic sclerite, median posterior projection: (0) absent, (1) present (Fig. S9A); *ci*=100, *ri*=100.

95. Male, phallic complex, endophallic sclerite, if median posterior projection is present (94(1)), size: (0) short, not surpassing half of ectophallic fold (Fig. S6Db), (1) long, through all ectophallic fold (Fig. S9A); *ci*=100, *ri*=100.

State 1 is a characteristic of *Eidmanacris*. There is a hyper development of this structure along with the ectophallic fold in *Eidmanacris endophallica*. This character was proposed by Souza-Dias [4].

96. Male, phallic complex, endophallic sclerite, if median posterior projection is present (94(1)), and it is long(95(1)),lateral thickening: (0) absent (Fig. S9Da), (1) present (Fig. S9A, Db, Dc); *ci*=100, *ri*=100.

97. Male, phallic complex, endophallic sclerite, if median posterior projection is present (94(1)), is long(95(1)), and has a lateral thickening (96(1)), position of lateral thickening (ACCTRAN) (Fig. S9D): (0) anterior, (1) median; *ci*=100, *ri*=100.

98. Male, phallic complex, endophallic sclerite, latero-posterior lobes (Fig. S9A): (0) absent, (1) present; *ci*=100, *ri*=100.

**References**

1. Robillard T, Desutter-Grandcolas L. Phylogeny and the modalities of acoustic diversification in extant Eneopterinae (Insecta, Orthoptera, Grylloidea, Eneopteridae). Cladistics. 2004;20: 271–293. doi:10.1111/j.1096-0031.2004.00025.x

2. Nihei SS. O gênero *Eidmanacris* (Chopard, 1956): Novas espécies e um estudo do relacionamento filogenético (Orthoptera: Grylloidea: Phalangopsidae: Luzarinae). Universidade Estadual Paulista “Júlio de Mesquita Filho.” 1997.

3. Campos LD de, Souza-Dias PGB, Nihei SS. Taxonomic review of *Eidmanacris* Chopard, 1956 (Orthoptera: Grylloidea: Phalangopsidae). Zootaxa. 2017;4321: 1–93. doi:10.11646/zootaxa.4321.1.1

4. Souza-Dias PGB de. Análise cladística e morfologia do complexo fálico de Phalangopside, com ênfase em Luzarinae (Orthoptera, Ensifera, Grylloidea). Instituto de Biociências da Universiadade de São Paulo. 2015;1: 72.

5. Prado RA, Fontanetti CS. Metanotal gland of the genus *Eidmanacris* (Grylloidea, Phalangopsidae): taxonomic importance. Iheringia Série Zoologia. 2005;95: 83–87. doi:10.1590/S0073-47212005000100012

6. Souza-Dias PGB, Campos LD de, Nihei SS. Two New Species of Eidmanacris (Orthoptera: Grylloidea: Phalangopsidae) from the Atlantic Forest of São Paulo State, Brazil. Florida Entomologist. 2015;98: 547–555. doi:10.1653/024.098.0223

7. De Mello FAG, De Andrade MApBS. *Ottedana cercalis*: a new genus and species of phalangopsid cricket from the Mantiqueira Range of southeastern Brazil (Orthoptera: Grylloidea). Journal of Orthoptera Research. 2003;12: 141–148. doi:10.1665/1082-6467(2003)012[0141:OCANGA]2.0.CO;2

8. Desutter-Grandcolas L. *Luzarida* Hebard, 1928 et genres affines: genres nouveaux, phylogénie et scénarios (Orthoptera, Grylloidea, Phalangopsidae, Luzarinae). Revue française Entomologique. 1993;15: 169–182.
