# Supplementary material for: Colonization of different biomes drove the diversification of the Neotropical Eidmanacris crickets (Insecta: Orthoptera: Grylloidea: Phalangopsidae)
Source: PLoS One. 2021 Jan 15;16(1):e0245325. doi: 10.1371/journal.pone.0245325 (PMC7810296; doi:10.1371/journal.pone.0245325)
Supplement: S4 File — (DOCX) [file pone.0245325.s024.docx]

*Analysis with 38 terminals, including* **E. paramarmorata**

This analysis generated 11 most parsimonious trees, with 240 steps, *ci*=0.47 and *ri*=0.78. Then, a strict consensus tree was obtained, the ambiguous and unambiguous characters were optimized, and Bremer support was calculated (Fig. S20).

Our results showed *Eidmanacris* as monophyletic and sustained by seven exclusive synapomorphies (Table 2). The clade is relatively well supported (Bremer support = 11).

The species *E. paramarmorata* acted as a wildcard (Figs S20) in the clade N2, which is defined by the female character 43(1) “posterior aperture of copulatory papilla large”. This species appears in each resulting tree grouped with a different terminal, however, it does not group with terminals from other clades.
