# Supplementary material for: Colonization of different biomes drove the diversification of the Neotropical Eidmanacris crickets (Insecta: Orthoptera: Grylloidea: Phalangopsidae)
Source: PLoS One. 2021 Jan 15;16(1):e0245325. doi: 10.1371/journal.pone.0245325 (PMC7810296; doi:10.1371/journal.pone.0245325)
Supplement: S1 Table — (DOCX) [file pone.0245325.s025.docx]

Table S1. Material examined for the ingroup taxa included in the phylogenetic analysis. ANSP, Academy of Natural Sciences of Philadelphia; MNHN, Muséum national d’Histoire naturelle; MZSP, Museu de Zoologia da Universidade de São Paulo; MZUEFS, Museu de Zoologia da Universidade Federal de Feira de Santana; UBTU, Laboratório de Insetos do Departamento de Zoologia da UNESP de Botucatu.

| **Taxon** | **SEM**  **analysis** | **Analyzed material** | **Locality** |
| --- | --- | --- | --- |
| *Eidmanacris alboannulata* (Piza, 1960) | x | Type material MNHN and UBTU | BR, São Paulo, Itirapina, Fazenda da Toca |
| *Eidmanacris bernardii* Nihei & de Mello, 2015 |  | Type material MZSP | BR, Goiás, Serra da Mesa |
| *Eidmanacris bidentata* Sperber, 1998 | x | Type material MZSP | BR, Minas Gerais, Viçosa, Mata da Biologia, UFV |
| *Eidmanacris caipira* Souza-Dias, Campos & Nihei, 2015 | x | Type material MZSP | BR, São Paulo, Teodoro Sampaio, Parque Estadual Morro do Diabo |
| *Eidmanacris corumbatai* Garcia, 1998 | x | Type material MZSP | BR, São Paulo, Cerrado de Corumbatai |
| *Eidmanacris desutterae* Campos, 2017 |  | Type material MZSP | BR, Mato Grosso do Sul, Aquidauna, Fazenda experimental da UEMS |
| *Eidmanacris dissimilis* Desutter-Grandcolas, 1995 | x | Type material MNHN; topotypes MZUEFS | BR, Minas Gerais, Poços de Caldas, Morro do Ferro |
| *Eidmanacris eliethae* Nihei & de Mello, 2015 |  | Type material MZSP and UBTU | BR, Rio de Janeiro, Rio das Ostras |
| *Eidmanacris endophallica* (de Mello, 1990) | x | Type material MZSP and UBTU | BR, Rio de Janeiro, Teresópolis |
| *Eidmanacris fontanettiae* Campos, Nihei & de Mello, 2017 | x | Type material MZSP and UBTU | BR, Espírito Santo, Santa Teresa |
| *Eidmanacris fusca* Desutter-Grandcolas, 1995 | x | MZSP and UBTU | BR, Santa Catarina, Urubici; BR, Santa Catarina, São Bento do Sul |
| *Eidmanacris gigas* Campos, 2017 | x | Type material MZSP | BR, Goiás, Pirenópolis |
| *Eidmanacris larvaeformis* (Chopard, 1938) | x | Type material MNHN | BR, Rio de Janeiro, Mendes |
| *Eidmanacris marmorata* (Bruner, 1916) |  | Type material ANSP | Bolivia, La Paz, Prov. Sara |
| *Eidmanacris melloi* Campos, 2017 | x | Type material MZSP and UBTU | BR, São Paulo, São Luiz do Paraitinga |
| *Eidmanacris meridionalis* Desutter-Grandcolas, 1995 | x | Type material MNHN; topotypes MZSP | BR, Santa Catarina, Nova Teutônia |
| *Eidmanacris minuta* (de Mello, 1990) | x | Type material MZSP and UBTU | BR, Rio de Janeiro, Teresópolis |
| *Eidmanacris multispinosa* Desutter-Grandcolas, 1995 |  | Type material MNHN; topotypes MZSP | BR, Espírito Santo, Santa Teresa, Reserva Santa Lúcia |
| *Eidmanacris neomarmorata* Campos, 2017 | x | Type material MZSP | BR, Mato Grosso, Chapada dos Guimarães |
| *Eidmanacris papaveroi* Nihei & de Mello, 2015 | x | Type material MZSP and UBTU | BR, Espírito Santo, Santa Teresa, Reserva Santa Lúcia |
| *Eidmanacris putuhra* Campos, 2017 | x | Type material MZSP and UBTU | BR, Minas Gerais, Viçosa |
| *Eidmanacris scopula* Campos, 2017 |  | Type material MZSP | BR, Tocantins, Dianópolis |
| *Eidmanacris septentrionalis* Desutter-Grandcolas, 1995 | x | Type material MNHN; topotypes UBTU | BR, Espírito Santo, Linhares, Reserva Natural Vale |
| *Eidmanacris simoesi* Nihei & de Mello, 2015 | x | Type material MZSP and UBTU | BR, Rio de Janeiro, Rio das Ostras |
| *Eidmanacris speluncae* (Mello-Leitão, 1937) | x | UBTU | BR, Minas Gerais, Santa Barbara |
| *Eidmanacris suassunai* Souza-Dias, Campos & Nihei, 2015 | x | Type material MZSP | BR, São Paulo, Teodoro Sampaio, Parque Estadual Morro do Diabo |
| *Eidmanacris tridentata* Desutter-Grandcolas, 1995 | x | Type material MNHN; topotypes UBTU | BR, Espírito Santo, Santa Teresa, Reserva Santa Lúcia |
