# Supplementary material for: Colonization of different biomes drove the diversification of the Neotropical Eidmanacris crickets (Insecta: Orthoptera: Grylloidea: Phalangopsidae)
Source: PLoS One. 2021 Jan 15;16(1):e0245325. doi: 10.1371/journal.pone.0245325 (PMC7810296; doi:10.1371/journal.pone.0245325)
Supplement: S2 Table — (DOCX) [file pone.0245325.s026.docx]

Table S2. Material examined for outgroup taxa included in the phylogenetic analysis.

| **Taxon** | **SEM**  **analysis** | **Analyzed material** | **Locality** |
| --- | --- | --- | --- |
| *Melanotes ornata* Desutter-Grandcolas, 1993 |  | Type material MNHN and topotype MZSP | BR, Espírito Santo, Santa Teresa, Reserva Santa Lúcia. |
| *Guabamima lordelloi* de Mello, 1993 | x | Type material MZSP and UBTU | BR, Bahia, Porto Seguro, Mata da CEPLAC; BR, Bahia, Una, Reserva Biológica de Una |
| *Guabamima saiva* de Mello, 1993 |  | Type material MZSP | BR, Rio de Janeiro, Rezende, distrito de Penedo |
| *Modestozara* sp. |  | MNHN | Equador, Aguyacu; Equador, Fatima |
| *Adenopygus heikoi* Bolfarini & de Mello, 2012 | x | Type material MZSP | BR, São Paulo, São José dos Campos, dist. São Francisco Xavier |
| *Bambuina bambui* de Mello, Horta & Bolfarini, 2013 | x | Type material MZSP and UBTU | BR, Minas Gerais, Mariana, Gruta do Centenário |
| *Ottedana cercalis* de Mello & Andrade, 2003 | x | Type material MZSP and UBTU | BR, São Paulo, Campos do Jordão |
| *Strinatia brevipennis* Chopard, 1970 | x | Type material MNHN | BR, São Paulo, Iporanga, Gruta das Areias |
| *Strinatia teresopolis*, Mesa, 1999 | x | Type material MZSP | BR, Rio de Janeiro, Teresópolis |
