# Supplementary material for: Colonization of different biomes drove the diversification of the Neotropical Eidmanacris crickets (Insecta: Orthoptera: Grylloidea: Phalangopsidae)
Source: PLoS One. 2021 Jan 15;16(1):e0245325. doi: 10.1371/journal.pone.0245325 (PMC7810296; doi:10.1371/journal.pone.0245325)
Supplement: S3 Table — (DOCX) [file pone.0245325.s027.docx]

Table S3. Data matrix: terminals and characters, part 1.

| **Coluna1** | **1** | **2** | **3** | **4** | **5** | **6** | **7** | **8** | **9** | **10** | **11** | **12** | **13** | **14** | **15** | **16** | **17** | **18** | **19** | **20** | **21** | **22** | **23** | **24** | **25** | **26** | **27** | **28** | **29** | **30** | **31** | **32** | **33** |
| --- | --- | --- | --- | --- | --- | --- | --- | --- | --- | --- | --- | --- | --- | --- | --- | --- | --- | --- | --- | --- | --- | --- | --- | --- | --- | --- | --- | --- | --- | --- | --- | --- | --- |
| *Melanotes ornata* | 0 | 0 | 0 | 0 | 0 | 0 | 0 | 0 | 0 | - | 0 | 0 | 0 | 0 | 0 | - | - | - | - | - | 0 | - | - | 0 | - | - | 0 | - | 0 | - | 0 | 0 | - |
| *Guabamima lordelloi* | 0 | 0 | 1 | 0 | 0 | 1 | 1 | 0 | 0 | - | 0 | 0 | 1 | 0 | 0 | - | - | - | - | - | 0 | - | - | 0 | - | - | 0 | - | 0 | - | 0 | 0 | - |
| *Guabamima saiva* | 0 | 0 | 1 | 0 | 0 | 1 | 1 | 0 | 0 | - | 0 | 0 | 1 | 0 | 0 | - | - | - | - | - | 0 | - | - | 0 | - | - | 0 | - | 0 | - | 0 | 0 | - |
| *Modestozara* sp. | 1 | 0 | 0 | 1 | 0 | 1 | 0 | 1 | 1 | 0 | 1 | 1 | 0 | 1 | 0 | - | - | - | - | - | 0 | - | - | 0 | - | - | 0 | - | 0 | - | 0 | 0 | - |
| *Ottedana cercalis* | 1 | 0 | 0 | 1 | 0 | 1 | 1 | 1 | 1 | 0 | 0 | 1 | 1 | 1 | 1 | 0 | 0 | - | 0 | 1 | - | - | - | 0 | - | - | 1 | 0 | 0 | - | 0 | 0 | - |
| *Adenopygus heikoi* | 2 | 0 | 0 | 1 | 1 | 0 | 1 | 1 | 1 | 0 | 1 | 1 | 0 | 1 | 1 | 0 | 0 | - | 0 | 1 | - | - | - | 0 | - | - | 0 | - | 0 | - | 0 | 0 | - |
| *Bambuina bambui* | 2 | 0 | 0 | 0 | 1 | 0 | 1 | 1 | 1 | 1 | 0 | 1 | 1 | 1 | 1 | 0 | 0 | - | 1 | 0 | 0 | - | - | 0 | - | - | 0 | - | 0 | - | 0 | 0 | - |
| *Strinatia brevipennis* | 1 | 0 | 0 | 0 | 0 | 1 | 1 | 1 | 1 | 0 | 0 | 1 | 0 | 1 | 1 | 0 | 0 | - | 0 | 0 | 1 | 0 | 0 | 0 | - | - | 1 | 0 | 0 | - | 0 | 0 | - |
| *Strinatia teresopolis* | 1 | 0 | 0 | 0 | 0 | 1 | 0 | 1 | 1 | 0 | 0 | 1 | 0 | 1 | 1 | 0 | 0 | - | 0 | 1 | - | - | - | 0 | - | - | 0 | - | 0 | - | 0 | 0 | - |
| *Eidmanacris minuta* | 2 | 1 | 1 | 1 | 2 | 0 | 1 | 1 | 1 | 0 | 1 | 1 | 0 | 1 | 1 | 0 | 0 | - | 0 | 0 | 1 | 0 | 1 | 0 | - | - | 1 | 0 | 1 | 1 | 0 | 1 | 0 |
| *Eidmanacris endophallica* | 1 | 1 | 1 | 1 | 2 | 0 | 1 | 1 | 1 | 0 | 1 | 1 | 0 | 1 | 0 | - | - | - | - | - | - | - | - | 0 | - | - | 0 | - | 1 | 1 | 0 | 1 | 1 |
| *Eidmanacris tridentata* | 2 | 1 | 1 | 1 | 0 | 0 | 1 | 1 | 1 | 0 | 1 | 1 | 0 | 1 | 0 | - | - | - | - | - | - | - | - | - | - | - | 0 | - | 1 | 0 | 1 | 1 | 0 |
| *Eidmanacris simoesi* | 2 | 1 | 1 | 1 | 0 | 0 | 1 | 1 | 1 | 0 | 1 | 1 | 0 | 1 | 1 | 0 | 1 | 0 | 2 | 0 | 0 | - | - | 1 | 1 | - | 1 | 0 | 1 | 0 | 1 | 1 | 0 |
| *Eidmanacris eliethae* | 2 | 1 | 1 | 1 | 0 | 0 | 1 | 1 | 1 | 0 | 0 | 1 | 0 | 1 | 1 | ? | 1 | 0 | 2 | 0 | ? | ? | ? | 1 | 0 | 1 | 1 | 0 | 1 | 0 | 1 | 1 | 0 |
| *Eidmanacris papaveroi* | 2 | 1 | 1 | 1 | 0 | 0 | 1 | 1 | 1 | 2 | 1 | 1 | 1 | 1 | 1 | 0 | 1 | 0 | 2 | 0 | 0 | - | - | 1 | 1 | - | 1 | 0 | 0 | - | 1 | 1 | 0 |
| *Eidmanacris bidentata* | 1 | 1 | 1 | 1 | 0 | 0 | 0 | 1 | 1 | 0 | 1 | 1 | 0 | 1 | 1 | 0 | 0 | 0 | 2 | 1 | - | - | - | 1 | 0 | 0 | 0 | - | 1 | 0 | 1 | 1 | 1 |
| *Eidmanacris putuhra* | 1 | 1 | 1 | 1 | 0 | 0 | 1 | 1 | 1 | 0 | 1 | 1 | 0 | 1 | 1 | 0 | 1 | 0 | 2 | 0 | 0 | - | - | 1 | 0 | 0 | 1 | 0 | 0 | 0 | 1 | 1 | 0 |
| *Eidmanacris speluncae* | 2 | 1 | 1 | 2 | 0 | 0 | 0 | 1 | 1 | 0 | 1 | 1 | 1 | 1 | 1 | 1 | 1 | 0 | 2 | 0 | 0 | - | - | 1 | 0 | 1 | 1 | 0 | 0 | - | 1 | 1 | 0 |
| *Eidmanacris fontanettiae* | 2 | 1 | 1 | 1 | 0 | 0 | 0 | 1 | 1 | 2 | 1 | 1 | 1 | 1 | 1 | 0 | 1 | 0 | 2 | 0 | 0 | - | - | 1 | 0 | 1 | 1 | 0 | 0 | - | 1 | 1 | 0 |

Table S3. Data matrix: terminals and characters, part 2.

| **Coluna1** | **1** | **2** | **3** | **4** | **5** | **6** | **7** | **8** | **9** | **10** | **11** | **12** | **13** | **14** | **15** | **16** | **17** | **18** | **19** | **20** | **21** | **22** | **23** | **24** | **25** | **26** | **27** | **28** | **29** | **30** | **31** | **32** | **33** |
| --- | --- | --- | --- | --- | --- | --- | --- | --- | --- | --- | --- | --- | --- | --- | --- | --- | --- | --- | --- | --- | --- | --- | --- | --- | --- | --- | --- | --- | --- | --- | --- | --- | --- |
| *Eidmanacris larvaeformis* | 2 | 1 | 1 | 2 | 0 | 0 | 0 | 1 | 1 | 2 | 1 | 1 | 1 | 1 | 1 | 1 | 1 | 0 | 2 | 0 | 0 | - | - | 1 | 0 | 1 | 1 | 0 | 1 | 0 | 1 | 1 | 0 |
| *Eidmanacris septentrionalis* | 2 | 1 | 1 | 1 | 0 | 0 | 0 | 1 | 1 | 2 | 1 | 1 | 1 | 1 | 1 | 1 | 1 | 0 | 2 | 0 | 0 | - | - | 1 | 0 | 1 | 1 | 0 | 1 | 0 | 1 | 1 | 0 |
| *Eidmanacris multispinosa* | 1 | 1 | 1 | 2 | 0 | 0 | 0 | 1 | 1 | 2 | 0 | 1 | 1 | 1 | 1 | ? | 1 | 0 | 2 | 0 | ? | ? | ? | 1 | 0 | 1 | 1 | 0 | 1 | 0 | 1 | 1 | 0 |
| *Eidmanacris alboannulata* | 2 | 1 | 1 | 1 | 0 | 0 | 0 | 1 | 1 | 0 | 0 | 1 | 0 | 1 | 1 | 0 | 1 | 0 | 2 | 0 | 1 | 1 | - | 1 | 0 | 1 | 1 | 1 | 1 | 0 | 1 | 1 | 1 |
| *Eidmanacris suassunai* | 2 | 1 | 1 | 1 | 0 | 0 | 0 | 1 | 1 | 0 | 1 | 1 | 0 | 1 | 1 | 0 | 1 | 0 | 2 | 0 | 1 | 1 | - | 1 | 0 | 1 | 1 | 1 | 1 | 0 | 1 | 1 | 1 |
| *Eidmanacris dissimilis* | 2 | 1 | 1 | 1 | 0 | 0 | 0 | 1 | 1 | 0 | 0 | 2 | 1 | 1 | 1 | 0 | 1 | 0 | 2 | 0 | 1 | 1 | - | 1 | 0 | 1 | 1 | 1 | 1 | 0 | 1 | 1 | 0 |
| *Eidmanacris meridionalis* | 2 | 1 | 1 | 1 | 0 | 0 | 0 | 1 | 1 | 0 | 0 | 2 | 1 | 1 | 1 | 0 | 1 | 0 | 2 | 0 | 1 | 1 | - | 1 | 0 | 1 | 1 | 1 | 1 | 0 | 1 | 1 | 0 |
| *Eidmanacris marmorata* | 2 | 1 | 1 | 1 | 0 | 0 | 0 | 1 | 1 | 2 | 0 | 1 | ? | 1 | 1 | ? | 1 | 1 | 2 | 0 | ? | ? | ? | 1 | 0 | 1 | 1 | ? | 1 | 0 | 1 | 1 | 0 |
| *Eidmanacris longa* | ? | 1 | 1 | 1 | 0 | 0 | 0 | 1 | 1 | 2 | 0 | 1 | ? | 1 | 1 | ? | 1 | 1 | 2 | 0 | ? | ? | ? | 1 | 0 | 1 | 1 | ? | 1 | 0 | 1 | 1 | 0 |
| *Eidmanacris paramarmorata* | ? | ? | ? | ? | ? | ? | ? | ? | ? | ? | ? | ? | ? | ? | ? | ? | ? | ? | ? | ? | ? | ? | ? | ? | ? | ? | ? | ? | ? | ? | ? | ? | ? |
| *Eidmanacris corumbatai* | 2 | 1 | 1 | 1 | 0 | 0 | 0 | 1 | 1 | 2 | 0 | 1 | 1 | 1 | 1 | 0 | 1 | 1 | 2 | 0 | 0 | - | - | 1 | 0 | 1 | 1 | 1 | 1 | 0 | 1 | 1 | 1 |
| *Eidmanacris caipira* | 2 | 1 | 1 | 1 | 0 | 0 | 0 | 1 | 1 | 2 | 0 | 1 | 1 | 1 | 1 | 1 | 1 | 1 | 2 | 0 | 0 | - | - | 1 | 0 | 1 | 1 | 1 | 1 | 0 | 1 | 1 | 0 |
| *Eidmanacris bernardii* | 2 | 1 | 1 | 1 | 0 | 0 | 0 | 1 | 1 | 2 | 0 | 1 | 1 | 1 | 1 | ? | 1 | 1 | 2 | 0 | ? | ? | ? | 1 | 0 | 1 | 1 | 1 | 1 | 0 | 1 | 1 | 0 |
| *Eidmanacris scopula* | 2 | 1 | 1 | 1 | 0 | 0 | 0 | 1 | 1 | 2 | 0 | 1 | 1 | 1 | ? | ? | ? | ? | ? | ? | ? | ? | ? | 1 | 0 | 0 | 0 | - | 1 | 0 | 1 | 1 | 1 |
| *Eidmanacris desutterae* | 2 | 1 | 1 | 1 | 0 | 0 | 0 | 1 | 1 | 2 | 0 | 1 | 0 | 1 | 1 | ? | 0 | - | 0 | 1 | - | - | - | 1 | 0 | 0 | 0 | - | 1 | 0 | 1 | 1 | 1 |
| *Eidmanacris gigas* | 2 | 1 | 1 | 1 | 0 | 0 | 0 | 1 | 1 | 2 | 0 | 1 | 1 | 1 | 1 | 0 | 1 | 1 | 2 | 0 | 0 | - | - | 1 | 0 | 0 | 1 | 1 | 1 | 0 | 1 | 1 | 0 |
| *Eidmanacris neomarmorata* | 2 | 1 | 1 | 1 | 0 | 0 | 0 | 1 | 1 | 2 | 0 | 1 | 1 | 1 | 1 | 0 | 1 | 1 | 2 | 0 | 0 | - | - | 1 | 0 | 1 | 1 | 1 | 1 | 0 | 1 | 1 | 1 |
| *Eidmanacris fusca* | 1 | 0 | 0 | 1 | 2 | 0 | 0 | 1 | 1 | 0 | 1 | 1 | 0 | 1 | 0 | - | - | - | - | - | - | - | - | 0 | - | - | 0 | - | 0 | - | 0 | 1 | 0 |
| *Eidmanacris melloi* | 1 | 0 | 0 | 1 | 2 | 0 | 1 | 1 | 1 | 0 | 1 | 1 | 0 | 1 | 1 | 0 | 0 | - | 0 | 0 | 1 | 0 | 1 | 0 | - | - | 1 | 0 | 1 | 1 | 0 | 1 | 0 |

Table S3. Data matrix: terminals and characters, part 1 continuation.

| **Coluna1** | **34** | **35** | **36** | **37** | **38** | **39** | **40** | **41** | **42** | **43** | **44** | **45** | **46** | **47** | **48** | **49** | **50** | **51** | **52** | **53** | **54** | **55** | **56** | **57** | **58** | **59** | **60** | **61** | **62** | **63** | **64** | **65** | **66** |
| --- | --- | --- | --- | --- | --- | --- | --- | --- | --- | --- | --- | --- | --- | --- | --- | --- | --- | --- | --- | --- | --- | --- | --- | --- | --- | --- | --- | --- | --- | --- | --- | --- | --- |
| *Melanotes ornata* | 0 | 0 | 0 | 0 | 0 | 0 | 0 | 0 | 0 | 0 | 0 | 0 | 0 | 0 | 0 | 0 | 0 | 0 | 0 | - | 0 | - | 0 | 0 | - | - | 0 | - | 0 | - | 0 | 0 | 0 |
| *Guabamima lordelloi* | 0 | 0 | 0 | 0 | 1 | 0 | 0 | 0 | 1 | 0 | 0 | 0 | 0 | 0 | 1 | 0 | 0 | 0 | 0 | - | 0 | - | 0 | 0 | - | - | 0 | - | 0 | - | 0 | 0 | 0 |
| *Guabamima saiva* | 0 | 0 | 0 | 0 | 1 | 0 | 0 | 0 | 1 | 0 | 0 | 0 | 0 | 0 | 1 | 0 | 0 | 0 | 0 | - | 0 | - | 0 | 0 | - | - | 0 | - | 0 | - | 0 | 0 | 0 |
| *Modestozara* sp. | 0 | 1 | 1 | 1 | 0 | 1 | 0 | 0 | 0 | ? | ? | 1 | 0 | 0 | 1 | 0 | 0 | 0 | 0 | - | 0 | - | 0 | 0 | - | - | 0 | - | 0 | - | 0 | 0 | 0 |
| *Ottedana cercalis* | 0 | 1 | 0 | 0 | 0 | 1 | 0 | 0 | 0 | 0 | 1 | 1 | 0 | 1 | 1 | 0 | 0 | 1 | 1 | 0 | 0 | - | 0 | 1 | 0 | 0 | 0 | - | 0 | - | 0 | 0 | 1 |
| *Adenopygus heikoi* | 0 | 1 | 0 | 0 | 0 | 1 | 0 | 0 | 0 | 0 | 0 | 1 | 0 | 0 | 2 | 0 | 0 | 1 | 1 | 0 | 1 | 0 | 0 | 0 | - | - | 0 | - | 0 | - | 0 | 1 | 0 |
| *Bambuina bambui* | 0 | 1 | 1 | 0 | 1 | 1 | 0 | 0 | 0 | 0 | 0 | 1 | 0 | 0 | 2 | 0 | 0 | 1 | 1 | 0 | 1 | 0 | 0 | 0 | - | - | 0 | - | 0 | - | 0 | 1 | 0 |
| *Strinatia brevipennis* | 0 | 1 | 1 | 1 | 1 | 1 | 0 | 0 | 1 | 0 | 1 | 1 | 0 | 1 | 1 | 0 | 0 | 0 | 1 | 0 | 0 | - | 0 | 1 | 0 | 0 | 0 | - | 0 | - | 0 | 1 | 1 |
| *Strinatia teresopolis* | 0 | 1 | 1 | 1 | 1 | 1 | 0 | 0 | 1 | 0 | 1 | 1 | 0 | 0 | 1 | 0 | 0 | 1 | 1 | 0 | 0 | - | 0 | 1 | 0 | 0 | 0 | - | 0 | - | 0 | 1 | 0 |
| *Eidmanacris minuta* | 0 | 1 | 1 | 0 | 0 | 1 | 1 | - | 1 | 0 | 0 | 1 | 1 | 0 | 1 | 1 | 1 | 0 | 1 | 0 | 1 | 0 | 0 | 1 | 0 | 0 | 0 | - | 0 | - | 1 | 1 | 1 |
| *Eidmanacris endophallica* | 0 | 1 | 1 | 0 | 0 | 1 | 1 | - | 1 | 0 | 0 | 1 | 1 | 0 | 1 | 1 | 1 | 0 | 1 | 0 | 0 | - | 0 | 1 | 0 | 0 | 0 | - | 0 | - | 1 | 1 | 1 |
| *Eidmanacris tridentata* | 0 | 1 | 1 | 0 | 1 | 1 | 0 | 0 | 2 | 0 | 0 | 1 | 1 | 0 | 1 | 0 | 1 | 1 | 1 | 0 | 1 | 0 | 0 | 1 | 0 | 0 | 0 | - | 0 | - | 1 | 1 | 1 |
| *Eidmanacris simoesi* | 0 | 1 | 1 | 0 | 1 | 1 | 0 | 0 | 2 | 0 | 0 | 1 | 1 | 0 | 1 | 0 | 1 | 1 | 1 | 0 | 1 | 0 | 0 | 1 | 0 | 0 | 0 | - | 0 | - | 1 | 1 | 1 |
| *Eidmanacris eliethae* | 0 | 1 | 1 | 0 | 1 | 1 | 0 | 0 | 2 | 0 | 0 | 1 | 1 | 0 | 1 | 0 | 1 | 1 | 1 | 0 | 1 | 0 | 0 | 1 | 0 | 0 | 0 | - | 0 | - | 1 | 1 | 0 |
| *Eidmanacris papaveroi* | 0 | 1 | 1 | 0 | 1 | 1 | 0 | 0 | 2 | 0 | 0 | 1 | 1 | 0 | 1 | 0 | 0 | 1 | 1 | 0 | 1 | 1 | 0 | 1 | 0 | 0 | 0 | - | 0 | - | 1 | 1 | 1 |
| *Eidmanacris bidentata* | 0 | 1 | 1 | 0 | 1 | 1 | 0 | 0 | 2 | 0 | 0 | 1 | 1 | 0 | 1 | 0 | 0 | 1 | 1 | 0 | 0 | - | 0 | 1 | 0 | 0 | 0 | - | 0 | - | 1 | 1 | 0 |
| *Eidmanacris putuhra* | 0 | 1 | 1 | 0 | 1 | 1 | 0 | 0 | 2 | 0 | 0 | 1 | 0 | 0 | 1 | 0 | 0 | 1 | 1 | 0 | 1 | 0 | 0 | 1 | 0 | 0 | 0 | - | 0 | - | 1 | 1 | 1 |
| *Eidmanacris speluncae* | 0 | 1 | 1 | 0 | 1 | 1 | 0 | 0 | 2 | 0 | 0 | 1 | 1 | 0 | 1 | 0 | 0 | 1 | 1 | 0 | 1 | 0 | 0 | 1 | 0 | 0 | 0 | - | 0 | - | 1 | 1 | 0 |
| *Eidmanacris fontanettiae* | 0 | 1 | 1 | 0 | 1 | 1 | 0 | 0 | 2 | 0 | 0 | 1 | 1 | 0 | 1 | 0 | 0 | 1 | 1 | 0 | 1 | 0 | 0 | 0 | - | - | 0 | - | 0 | - | 0 | 1 | 1 |

Table S3. Data matrix: terminals and characters, part 2 continuation.

| **Coluna1** | **34** | **35** | **36** | **37** | **38** | **39** | **40** | **41** | **42** | **43** | **44** | **45** | **46** | **47** | **48** | **49** | **50** | **51** | **52** | **53** | **54** | **55** | **56** | **57** | **58** | **59** | **60** | **61** | **62** | **63** | **64** | **65** | **66** |
| --- | --- | --- | --- | --- | --- | --- | --- | --- | --- | --- | --- | --- | --- | --- | --- | --- | --- | --- | --- | --- | --- | --- | --- | --- | --- | --- | --- | --- | --- | --- | --- | --- | --- |
| *Eidmanacris larvaeformis* | 1 | 1 | 1 | 0 | 1 | 1 | 0 | 0 | 2 | 0 | 0 | 1 | 0 | 0 | 1 | 0 | 0 | 1 | 1 | 0 | 1 | 0 | 0 | 1 | 0 | 0 | 0 | - | 0 | - | 1 | 1 | 1 |
| *Eidmanacris septentrionalis* | 1 | 1 | 1 | 0 | 1 | 1 | 0 | 0 | 2 | 0 | 0 | 1 | 0 | 0 | 1 | 0 | 0 | 1 | 1 | 0 | 1 | 0 | 0 | 1 | 0 | 0 | 0 | - | 0 | - | 1 | 1 | 0 |
| *Eidmanacris multispinosa* | 0 | 1 | 1 | 0 | 1 | 1 | 0 | 0 | 2 | 0 | 0 | 1 | 0 | 0 | 1 | 0 | 1 | 1 | 1 | 0 | 1 | 1 | 0 | 1 | 0 | 0 | 0 | - | 0 | - | 1 | 1 | 0 |
| *Eidmanacris alboannulata* | 1 | 1 | 1 | 0 | 2 | 1 | 0 | 0 | 2 | 0 | 0 | 1 | 0 | 0 | 1 | 0 | 1 | 1 | 1 | 1 | 1 | 0 | 0 | 1 | 0 | 1 | 0 | - | 0 | - | 1 | 1 | 0 |
| *Eidmanacris suassunai* | 1 | 1 | 1 | 0 | 2 | 1 | 0 | 0 | 2 | 0 | 0 | 1 | 0 | 0 | 1 | 0 | 1 | 1 | 1 | 1 | 1 | 0 | 0 | 1 | 0 | 1 | 0 | - | 0 | - | 1 | 1 | 0 |
| *Eidmanacris dissimilis* | 0 | 1 | 1 | 0 | 2 | 1 | 0 | 0 | 2 | 0 | 0 | 1 | 1 | 0 | 1 | 0 | 1 | 1 | 1 | 0 | 1 | 0 | 0 | 1 | 0 | 0 | 0 | - | 0 | - | 1 | 1 | 0 |
| *Eidmanacris meridionalis* | 0 | 1 | 1 | 0 | 2 | 1 | 0 | 0 | 2 | 0 | 0 | 1 | 1 | 0 | 1 | 0 | 1 | 1 | 1 | 0 | 1 | 0 | 0 | 1 | 0 | 0 | 0 | - | 0 | - | 1 | 1 | 1 |
| *Eidmanacris marmorata* | ? | 1 | 1 | 0 | 1 | 1 | 0 | 1 | ? | 1 | 0 | 1 | 1 | 0 | 1 | 0 | 0 | 1 | 1 | 2 | 1 | 2 | 1 | 1 | 1 | 0 | 0 | - | 1 | 0 | 1 | 1 | 1 |
| *Eidmanacris longa* | ? | 1 | ? | 0 | 1 | 1 | 0 | 1 | 2 | 1 | 0 | 1 | 1 | 0 | 1 | 0 | 0 | 1 | 1 | 2 | 1 | 2 | 1 | 1 | 1 | 0 | 0 | - | 1 | 0 | 1 | 1 | 1 |
| *Eidmanacris paramarmorata* | ? | ? | ? | ? | ? | ? | 0 | 1 | 2 | 1 | 0 | ? | ? | ? | ? | ? | ? | ? | ? | ? | ? | ? | ? | ? | ? | ? | ? | ? | ? | ? | ? | ? | ? |
| *Eidmanacris corumbatai* | 1 | 1 | 1 | 0 | 1 | 1 | 0 | 1 | 2 | 1 | 0 | 1 | 1 | 2 | 1 | 0 | 0 | 1 | 1 | 1 | 1 | 2 | 1 | 1 | 1 | 0 | 1 | 1 | 1 | 0 | 1 | 1 | 1 |
| *Eidmanacris caipira* | 1 | 1 | 1 | 0 | 1 | 1 | 0 | 1 | 2 | 1 | 0 | 1 | 1 | 0 | 1 | 0 | 0 | 1 | 1 | 1 | 1 | 2 | 1 | 1 | 1 | 0 | 0 | - | 1 | 1 | 1 | 1 | 1 |
| *Eidmanacris bernardii* | 1 | 1 | 1 | 0 | 1 | 1 | ? | ? | ? | ? | ? | 1 | 1 | 0 | 1 | 0 | 0 | 1 | 1 | 1 | 1 | 2 | 1 | 1 | 1 | 0 | 0 | - | 1 | 1 | 1 | 1 | 1 |
| *Eidmanacris scopula* | 1 | 1 | 1 | 0 | 1 | 1 | 0 | 1 | 2 | 1 | 0 | 1 | 1 | 0 | 1 | 0 | 0 | 1 | 1 | 1 | 1 | 0 | 0 | 1 | 1 | 0 | 0 | - | 1 | 0 | 1 | 1 | 1 |
| *Eidmanacris desutterae* | 0 | 1 | 1 | 0 | 1 | 1 | 0 | 1 | 2 | 1 | 0 | 1 | 1 | 2 | 1 | 0 | 0 | 1 | 1 | 0 | 1 | 0 | 0 | 1 | 1 | 0 | 1 | 0 | 1 | 0 | 1 | 1 | 1 |
| *Eidmanacris gigas* | 0 | 1 | 1 | 0 | 1 | 1 | 0 | 1 | 2 | 1 | 0 | 1 | 0 | 2 | 1 | 0 | 0 | 1 | 1 | 1 | 1 | 2 | 1 | 1 | 1 | 0 | 1 | 1 | 1 | 0 | 1 | 1 | 1 |
| *Eidmanacris neomarmorata* | 1 | 1 | 1 | 0 | 1 | 1 | 0 | 1 | 2 | 1 | 0 | 1 | 1 | 0 | 1 | 0 | 0 | 1 | 1 | 2 | 1 | 2 | 1 | 1 | 1 | 0 | 0 | - | 1 | 0 | 1 | 1 | 1 |
| *Eidmanacris fusca* | 0 | 1 | 1 | 0 | 0 | 1 | 0 | 0 | 1 | 0 | 0 | 1 | 1 | 0 | 1 | 1 | 1 | 0 | 1 | 0 | 1 | 0 | 0 | 1 | 0 | 0 | 0 | - | 0 | - | 1 | 1 | 1 |
| *Eidmanacris melloi* | 0 | 1 | 1 | 0 | 1 | 1 | 0 | 0 | 1 | 0 | 0 | 1 | 1 | 0 | 1 | 1 | 1 | 0 | 1 | 0 | 1 | 0 | 0 | 1 | 0 | 0 | 0 | - | 0 | - | 1 | 1 | 1 |

Table S3. Data matrix: terminals and characters, part 1 continuation.

| **Coluna1** | **67** | **68** | **69** | **70** | **71** | **72** | **73** | **74** | **75** | **76** | **77** | **78** | **79** | **80** | **81** | **82** | **83** | **84** | **85** | **86** | **87** | **88** | **89** | **90** | **91** | **92** | **93** | **94** | **95** | **96** | **97** | **98** |
| --- | --- | --- | --- | --- | --- | --- | --- | --- | --- | --- | --- | --- | --- | --- | --- | --- | --- | --- | --- | --- | --- | --- | --- | --- | --- | --- | --- | --- | --- | --- | --- | --- |
| *Melanotes ornata* | 0 | 0 | 0 | - | 0 | 0 | 0 | 0 | 0 | - | 0 | 0 | 0 | - | - | 0 | 0 | 0 | - | 0 | 0 | 0 | 0 | 0 | 0 | - | - | 0 | - | - | - | 0 |
| *Guabamima lordelloi* | 0 | 0 | 0 | - | 0 | 0 | 0 | 0 | 0 | - | 0 | 1 | 0 | - | - | 1 | 0 | 0 | - | 1 | - | - | - | 0 | 0 | - | - | 0 | - | - | - | 0 |
| *Guabamima saiva* | 0 | 0 | 0 | - | 0 | 0 | 0 | 0 | 0 | - | 0 | 1 | 0 | - | - | 1 | 0 | 0 | - | 1 | - | - | - | 0 | 0 | - | - | 0 | - | - | - | 0 |
| *Modestozara* sp. | 0 | 0 | 0 | - | 0 | 0 | 0 | 0 | 1 | 0 | 0 | 0 | 0 | - | - | 0 | 0 | 0 | - | 1 | - | - | - | 0 | 1 | - | - | 1 | 0 | - | - | 0 |
| *Ottedana cercalis* | 0 | 1 | 0 | - | 1 | 0 | 0 | 0 | 0 | - | 0 | 0 | 1 | 0 | 0 | 0 | 0 | 0 | - | 1 | - | - | - | 0 | 1 | - | - | 1 | 0 | - | - | 0 |
| *Adenopygus heikoi* | 0 | 1 | 0 | - | 0 | 1 | 0 | 0 | 0 | - | 0 | 0 | 0 | - | - | 0 | 0 | 0 | - | 1 | - | - | - | 0 | 1 | - | - | 1 | 0 | - | - | 0 |
| *Bambuina bambui* | 0 | 1 | 0 | - | 0 | 1 | 0 | 0 | 0 | - | 0 | 0 | 0 | - | - | 0 | 0 | 0 | - | 1 | - | - | - | 0 | 1 | - | - | 1 | 0 | - | - | 0 |
| *Strinatia brevipennis* | 0 | 1 | 1 | 0 | 1 | 0 | 0 | 0 | 0 | - | 0 | 0 | 1 | 0 | 0 | 0 | 0 | 0 | - | 1 | - | - | - | 0 | 1 | - | - | 1 | 0 | - | - | 0 |
| *Strinatia teresopolis* | 0 | 1 | 1 | 0 | 1 | 0 | 0 | 0 | 0 | - | 0 | 0 | 0 | - | - | 0 | 0 | 0 | - | 1 | - | - | - | 0 | 1 | - | - | 1 | 0 | - | - | 0 |
| *Eidmanacris minuta* | 1 | 1 | 1 | 0 | 0 | 0 | 0 | 0 | 1 | 0 | 0 | 1 | 1 | 0 | 0 | 1 | 0 | 1 | 1 | 0 | 1 | 0 | 0 | 1 | - | 0 | 1 | 1 | 1 | 0 | - | 1 |
| *Eidmanacris endophallica* | 1 | 1 | 1 | 0 | 0 | 0 | 0 | 0 | 1 | 0 | 0 | 1 | 1 | 0 | 0 | 1 | 0 | 1 | 1 | 1 | - | - | - | 1 | - | 0 | 1 | 1 | 1 | 0 | - | 1 |
| *Eidmanacris tridentata* | 1 | 1 | 1 | 1 | 1 | 0 | 0 | 0 | 1 | 0 | 2 | 1 | 1 | 0 | 0 | 1 | 0 | 1 | 0 | 0 | 0 | - | - | 1 | - | 1 | 0 | 1 | 1 | 0 | - | 1 |
| *Eidmanacris simoesi* | 1 | 1 | 1 | 1 | 1 | 0 | 0 | 0 | 1 | 1 | 2 | 1 | 1 | 0 | 0 | 1 | 0 | 1 | 0 | 0 | 0 | 0 | 1 | 1 | - | 1 | 0 | 1 | 1 | 0 | - | 1 |
| *Eidmanacris eliethae* | 1 | 1 | 1 | 1 | 1 | 0 | 0 | 0 | 1 | 1 | 2 | 1 | 1 | 0 | 0 | 1 | 0 | 1 | 0 | 0 | 0 | 0 | 1 | 1 | - | 1 | 0 | 1 | 1 | 0 | - | 1 |
| *Eidmanacris papaveroi* | 1 | 1 | 1 | 1 | 1 | 0 | 0 | 0 | 1 | 0 | 0 | 1 | 1 | 1 | - | 1 | 1 | 1 | 0 | 0 | 1 | 0 | 1 | 1 | - | 1 | 0 | 1 | 1 | 0 | - | 1 |
| *Eidmanacris bidentata* | 1 | 1 | 1 | 0 | 1 | 0 | 0 | 0 | 1 | 0 | 0 | 1 | 1 | 0 | 0 | 1 | 0 | 1 | 0 | 0 | 1 | 0 | 0 | 1 | - | 0 | 0 | 1 | 1 | 0 | - | 1 |
| *Eidmanacris putuhra* | 1 | 1 | 1 | 1 | 1 | 0 | 0 | 0 | 1 | 0 | 0 | 1 | 1 | 1 | - | 2 | 0 | 1 | 0 | 0 | 0 | 0 | 1 | 1 | - | 0 | 0 | 1 | 1 | 0 | - | 1 |
| *Eidmanacris speluncae* | 1 | 1 | 1 | 0 | 1 | 0 | 0 | 0 | 1 | 0 | 0 | 1 | 1 | 1 | - | 2 | 1 | 1 | 0 | 0 | 0 | 0 | 1 | 1 | - | 0 | 0 | 1 | 1 | 0 | - | 1 |
| *Eidmanacris fontanettiae* | 1 | 1 | 1 | 0 | 1 | 0 | 0 | 0 | 1 | 0 | 0 | 1 | 1 | 0 | 0 | 1 | 0 | 1 | 0 | 0 | 0 | 0 | 1 | 1 | - | 1 | 0 | 1 | 1 | 0 | - | 1 |

Table S3. Data matrix: terminals and characters, part 2 continuation.

| **Coluna1** | **67** | **68** | **69** | **70** | **71** | **72** | **73** | **74** | **75** | **76** | **77** | **78** | **79** | **80** | **81** | **82** | **83** | **84** | **85** | **86** | **87** | **88** | **89** | **90** | **91** | **92** | **93** | **94** | **95** | **96** | **97** | **98** |
| --- | --- | --- | --- | --- | --- | --- | --- | --- | --- | --- | --- | --- | --- | --- | --- | --- | --- | --- | --- | --- | --- | --- | --- | --- | --- | --- | --- | --- | --- | --- | --- | --- |
| *Eidmanacris larvaeformis* | 1 | 1 | 1 | 0 | 1 | 0 | 1 | 0 | 1 | 0 | 2 | 1 | 1 | 0 | 0 | 1 | 0 | 1 | 0 | 0 | 1 | 0 | 0 | 1 | - | 0 | 0 | 1 | 1 | 0 | - | 1 |
| *Eidmanacris septentrionalis* | 1 | 1 | 1 | 0 | 1 | 0 | 0 | 0 | 1 | 0 | 0 | 1 | 1 | 0 | 0 | 1 | 1 | 1 | 0 | 0 | 0 | 0 | 0 | 1 | - | 0 | 0 | 1 | 1 | 0 | - | 1 |
| *Eidmanacris multispinosa* | 1 | 1 | 1 | 0 | 1 | 0 | 0 | 0 | 1 | 0 | 0 | 1 | 1 | 1 | - | 1 | 1 | 1 | 0 | 0 | 0 | 0 | 0 | 1 | - | 0 | 0 | 1 | 1 | 0 | - | 1 |
| *Eidmanacris alboannulata* | 1 | 1 | 1 | 0 | 1 | 0 | 1 | 0 | 0 | - | 1 | 1 | 1 | 0 | 0 | 1 | 0 | 1 | 0 | 0 | 0 | 0 | 0 | 1 | - | 0 | 0 | 1 | 1 | 1 | 0 | 1 |
| *Eidmanacris suassunai* | 1 | 1 | 1 | 0 | 1 | 0 | 1 | 0 | 0 | - | 1 | 1 | 1 | 0 | 0 | 1 | 0 | 1 | 0 | 0 | 0 | 0 | 0 | ? | - | 0 | 0 | 1 | 1 | 1 | 0 | 1 |
| *Eidmanacris dissimilis* | 1 | 1 | 1 | 0 | 1 | 0 | 1 | 0 | 0 | - | 1 | 1 | 1 | 0 | 0 | 1 | 0 | 1 | 0 | 0 | 0 | 0 | 0 | 1 | - | 0 | 0 | 1 | 1 | 1 | 0 | 1 |
| *Eidmanacris meridionalis* | 1 | 1 | 1 | 0 | 1 | 0 | 1 | 0 | 1 | 0 | 1 | 1 | 1 | 0 | 0 | 1 | 0 | 1 | 0 | 0 | 0 | 0 | 0 | 1 | - | 0 | 0 | 1 | 1 | 1 | 0 | 1 |
| *Eidmanacris marmorata* | 1 | 1 | 1 | 0 | 1 | 0 | 0 | 1 | 0 | - | 1 | 1 | 1 | 0 | 1 | 1 | ? | 1 | 0 | 0 | 0 | 0 | 0 | 1 | - | 0 | 0 | 1 | 1 | 1 | 1 | 1 |
| *Eidmanacris longa* | 1 | 1 | 1 | 0 | 1 | 0 | 0 | 1 | 0 | - | 1 | 1 | 1 | 0 | 1 | 1 | 2 | 1 | 0 | 0 | 0 | 0 | 0 | 1 | - | 0 | 0 | 1 | 1 | 1 | 1 | 1 |
| *Eidmanacris paramarmorata* | ? | ? | ? | ? | ? | ? | ? | ? | ? | ? | ? | ? | ? | ? | ? | ? | ? | ? | ? | ? | ? | ? | ? | ? | ? | ? | ? | ? | ? | ? | ? | ? |
| *Eidmanacris corumbatai* | 1 | 1 | 1 | 0 | 1 | 0 | 0 | 1 | 0 | - | 1 | 1 | 1 | 0 | 0 | 1 | 2 | 1 | 0 | 0 | 0 | 1 | - | 1 | - | 0 | 0 | 1 | 1 | 1 | 1 | 1 |
| *Eidmanacris caipira* | 1 | 1 | 1 | 0 | 1 | 0 | 0 | 1 | 0 | - | 1 | 1 | 1 | 0 | 1 | 1 | 2 | 1 | 0 | 0 | 0 | 0 | 0 | 1 | - | 0 | 0 | 1 | 1 | 1 | 1 | 1 |
| *Eidmanacris bernardii* | 1 | 1 | 1 | 0 | 1 | 0 | 0 | 1 | 0 | - | 1 | 1 | 1 | 0 | 1 | 1 | 0 | 1 | 0 | 0 | 0 | 1 | - | 1 | - | 0 | 0 | 1 | 1 | 1 | 1 | 1 |
| *Eidmanacris scopula* | 1 | 1 | 1 | 0 | 1 | 0 | 0 | 1 | 0 | - | 1 | 1 | 1 | 0 | 1 | 1 | 2 | 1 | 0 | 0 | 0 | 0 | 0 | 1 | - | 0 | 0 | 1 | 1 | 1 | 1 | 1 |
| *Eidmanacris desutterae* | 1 | 1 | 1 | 0 | 1 | 0 | 0 | 1 | 0 | - | 1 | 1 | 1 | 0 | 0 | 1 | 2 | 1 | 0 | 0 | 0 | 0 | 0 | 1 | - | 0 | 0 | 1 | 1 | 1 | 1 | 1 |
| *Eidmanacris gigas* | 1 | 1 | 1 | 0 | 1 | 0 | 0 | 1 | 0 | - | 1 | 1 | 1 | 0 | 0 | 1 | 2 | 1 | 0 | 0 | 0 | 0 | 0 | 1 | - | 0 | 0 | 1 | 1 | 1 | 1 | 1 |
| *Eidmanacris neomarmorata* | 1 | 1 | 1 | 0 | 1 | 0 | 0 | 1 | 0 | - | 1 | 1 | 1 | 0 | 1 | 1 | 2 | 1 | 0 | 0 | 0 | 0 | 0 | 1 | - | 0 | 0 | 1 | 1 | 1 | 1 | 1 |
| *Eidmanacris fusca* | 1 | 1 | 1 | 0 | 1 | 0 | 0 | 0 | 0 | - | 1 | 1 | 1 | 0 | 0 | 1 | 0 | 1 | 0 | 0 | 0 | 0 | 0 | 1 | - | 0 | 1 | 1 | 1 | 0 | - | 1 |
| *Eidmanacris melloi* | 1 | 1 | 1 | 0 | 0 | 0 | 0 | 0 | 1 | 0 | 0 | 1 | 1 | 0 | 0 | 1 | 0 | 1 | 1 | 0 | 1 | 0 | 0 | 1 | - | 0 | 1 | 1 | 1 | 0 | - | 1 |
