# Supplementary material for: Colonization of different biomes drove the diversification of the Neotropical Eidmanacris crickets (Insecta: Orthoptera: Grylloidea: Phalangopsidae)
Source: PLoS One. 2021 Jan 15;16(1):e0245325. doi: 10.1371/journal.pone.0245325 (PMC7810296; doi:10.1371/journal.pone.0245325)
Supplement: S4 Table — (DOCX) [file pone.0245325.s028.docx]

Table S4. Homoplastic synapomorphies of Eidmanacris.

| **Character/State** | **Optimization** | **State of character description** |
| --- | --- | --- |
| 6(0) | ACCTRAN | Sensillas region of maxillary palpus rounded |
| 11(1) | Not ambiguous | Apex of forewings not reaching posterior border of metanotum |
| 44(1) | ACCTRAN | Dorsal median furrow of copulatory papilla absent |
| 46(1) | Not ambiguous | Median third of base of pseudepiphallic sclerite narrower than lateral thirds in dorsal view |
| 54(1) | ACCTRAN | Presence of supero-internal projection of apex of pseudepiphallic arm |
| 75(1) | ACCTRAN | Ectophallic arc posterior to the base of pseudepiphallic sclerite |
| 78(1) | Not ambiguous | Ectophallic apodeme with same size or longer than pseudepiphallic arm in dorsal view |
| 82(1) | Not ambiguous | Apex of ectophallic apodeme straight |
| 86(0) | Not ambiguous | Presence of dorsal projection of ectophallic invagination |
