# Supplementary material for: Colonization of different biomes drove the diversification of the Neotropical Eidmanacris crickets (Insecta: Orthoptera: Grylloidea: Phalangopsidae)
Source: PLoS One. 2021 Jan 15;16(1):e0245325. doi: 10.1371/journal.pone.0245325 (PMC7810296; doi:10.1371/journal.pone.0245325)
Supplement: S5 Table — (DOCX) [file pone.0245325.s029.docx]

Table S5. Exclusive synapomorphies of the clades A to N of Eidmanacris.

| **Clade** | **Character/State** | | | **State of character description** |  |
| --- | --- | --- | --- | --- | --- |
| A | 5 (2) | | | Maxillary palpi sharply curved |  |
|  | 49 (1) | | | Apex of pseudepiphallic arms curved inwards |  |
|  | 93 (1) | | | Endophallic apodeme as long as wide |  |
| B | 19 (2) | | | Metanotum lateral projections parallel to each other |  |
|  | 24 (1) | | | Presence of median pronounced crest on anterior border of metanotum |  |
|  | 31 (1) | | | Male supra anal plate constricted medially |  |
|  | 42 (2) | | | Apex of ovipositor in dorsal view curved |  |
| D | 17 (1) | | | Metanotum lateral projections as long as wide |  |
|  | 70 (1) | | | Bifid apex of dorsal lobe of pseudepiphallic paramere |  |
| F | 26 (1) | | | Bristles of median crest of metanotum pointed laterally |  |
|  | 92 (1) | | | Endophallic apodeme flattened |  |
| H | 10 (2) | | | Triangular forewings in dorsal view |  |
| L | 34 (1) | | | Posterior border of supra anal plate straight |  |
| M | 4 (2) | | | Posterior half of antenna unpigmented |  |
| N | 28 (1) | | | Bristles occupying all dorsum of metanotum |  |
|  | 53 (1) | | | Superior projection of the apex of pseudepiphallic arm reduced to a spine |  |
|  | 96 (1) | | | Median posterior projection of endophallic sclerite with a lateral thickening on median region |  |
| N1 | | 22 (1) | Median projection of metanotum very reduced, visible only with SEM | |  |
|  |  | 38 (2) | Dorsal and median inner apical spurs of tibia III same-sized | |  |
| N2 | | 41 (1) | Presence of central invagination reaching the median part the subgenital plate of female | |  |
|  |  | 43 (1) | Posterior aperture of copulatory papilla large | |  |
|  |  | 58 (1) | Inferior projection of the apex of pseudepiphallic arm like an upcurved hook | |  |
|  |  | 62 (1) | Presence of lateral projection of pseudepiphallic sclerite | |  |
|  |  | 74 (1) | Ventral lobe of pseudepiphallic paramere up curved forming a 90° angle in lateral view | |  |
|  |  | 83 (2) | Ventral posterior projection of ectophallic invagination curved internally | |  |
|  |  | 97 (1) | Median posterior projection of endophallic sclerite with a lateral thickening on median region | |  |
